# A global mitochondrial genomic atlas illuminates freshwater microeukaryote diversity

**DOI:** 10.1038/s41467-026-78160-1

**Published:** 2026-09-25

**Authors:** Lucas Serra Moncadas, Hans-Joachim Ruscheweyh, Jason Woodhouse, Michaela M. Salcher, Yusuke Okazaki, David Kamanda Ngugi, Michael Pester, Thomas Posch, Rohit Ghai, Hans-Peter Grossart, Shinichi Sunagawa, Jakob Pernthaler, Adrian-Stefan Andrei

**Affiliations:** 1 Limnological Station, Department of Plant and Microbial Biology, University of Zurich, Kilchberg, Switzerland; 2 Institute of Microbiology, Department of Biology, ETH Zurich, Zurich, Switzerland; 3 Swiss Institute of Bioinformatics, Lausanne, Switzerland; 4 Department of Experimental Limnology, Leibniz Institute for Freshwater Ecology and Inland Fisheries, Stechlin, Germany; 5 Department of Aquatic Microbial Ecology, Institute of Hydrobiology, Biology Centre of the Czech Academy of Sciences, Ceské Budejovice, Czech Republic; 6 Bioinformatics Center, Institute for Chemical Research, Kyoto University, Uji, Japan; 7 GEOMAR Helmholtz Centre for Ocean Research Kiel, Kiel, Germany; 8 Chair of Microbial Physiology, Technical University of Munich, Freising, Germany; 9 Institute of Biochemistry and Biology, Potsdam University, Potsdam, Germany; 10 Berlin-Brandenburg Institute of Advanced Biodiversity Research, Free University, Berlin, Germany

**Keywords:** Water microbiology, Biodiversity, Freshwater ecology, Microbial ecology, Metagenomics

## Abstract

Environmental genomics has transformed our view of the microbial world, yet progress remains largely prokaryote-centric. Among microbial eukaryotes, inconsistent marker choice and limited species-level resolution blunt diversity estimates and hinder cross-study synthesis. Consequently, regional‑to‑global gradients in richness, turnover, and phylogenetic structure remain poorly resolved. Here we establish a global mitogenome-based framework that converts shotgun metagenomes into species‑resolved, phylogeny‑anchored inventories. From 3,400 metagenomes spanning 362 lakes on seven continents, we reconstruct ~20,000 mitochondrial metagenome-assembled genomes (mitoMAGs), densely populating previously sparse branches of the eukaryotic tree—notably SAR (Stramenopiles–Alveolata–Rhizaria), Cryptophyceae, and Haptista. An empirically calibrated 98.1% mitoMAG‑identity threshold delineates species and tethers community profiles to an evolutionary backbone. On this backbone, coverage‑standardized inventories recover canonical ecological patterns: mesotrophic richness peaks, surface-layer expansion of phylogenetic breadth, and spring maxima with the strongest surface–deep coupling at vernal mixing; supporting the framework. Treating mitoMAGs as primer‑free barcodes yields a single, species‑level, phylogeny‑aware currency that renders richness, evenness, and phylogenetic breadth directly comparable across datasets, depths, seasons, and regions. By placing microbial eukaryotes on an analytical footing comparable to prokaryotes, the resulting atlas brings their diversity into reach and lays the groundwork for future high-throughput, species-aware monitoring and ecological forecasting.

## Abstract (web summary)

Environmental genomics has focused mainly on prokaryotes, leaving microbial eukaryote diversity poorly understood. Using ~20,000 mitochondrial genomes from 3,400 lake metagenomes worldwide, this study creates a species-level framework that reveals global biodiversity patterns and enables scalable ecological monitoring.

## Introduction

Eukaryotes are masters of form across scales. Over eight orders of magnitude—from the ~1 µm picoalga *Ostreococcus tauri* to the >100 m coastal redwood *Sequoia sempervirens*—they sculpt matter into precise architectures: siliceous diatom frustules, Golgi-templated calcite coccoliths, fractal Romanesco inflorescences, pentaradial echinoderm arms. Pigmentary and structural colors—chlorophyll greens, carotenoid golds, melanin blacks—reinforce these signatures, which have long underpinned classification from the pre-Linnaean *scala naturae* to modern fieldkeys^1^.

Beneath this visible richness lies most eukaryotic diversity: in the microbial realm^2,3^. Microbial eukaryotes (microeukaryotes)—environmentally ubiquitous^4^ and largely unicellular—encompass most of the domain’s phylogenetic^5^ and metabolic breadth^6^. In the open ocean^7,8^ they generate more than half of the marine primary production^9^ and help regulate the nitrogen, phosphorus, and silica inventories^10–13^. In freshwater and marine ecosystems, heterotrophic flagellates, ciliates, and amoebae remove a significant fraction of daily bacterial production^14–16^, mobilize trace metals^17,18^, and, under conditions of nutrient imbalance, seed neuro- or hepatotoxic blooms^19,20^. Taken together, these roles place microeukaryotes at the core of aquatic food webs, channeling energy and elements from microbes to higher trophic levels.

At microbial scales, morphology has limited resolving power: many taxa are visually nondescript, and the few characters available are often homoplastic, with convergent architectures recurring across distant lineages and obscuring relatedness^21,22^. This limited resolution has concrete consequences: pathogenic *Entamoeba histolytica* is indistinguishable from the commensal *E. dispar*^23^, while saxitoxin-producing *Alexandrium catenella* hides among benign congeners^24^. Stripped of stable traits, morphology-based identification often stalls at coarse ranks, decoupling identity from function—with notable exceptions in ornate lineages^25,26^ that remain readily diagnosable by specialists.

Genomics should bridge this gap, yet it often falters in environmental samples. Microeukaryotic DNA is typically rare relative to prokaryotic one. Nuclear architectures compound the problem: dinoflagellates carry >200 Gb genomes in histone‑poor liquid‑crystal chromosomes^27,28^; ciliates split heredity between a repeat‑rich germline and tens of thousands of somatic nanochromosomes^29,30^. Even in milder cases, tandem repeats, intron‑dense genes, and pervasive heterozygosity fracture assemblies and confound binning^31^. Metabarcoding helps, yet plateaus: 18S rRNA is frequently genus‑bound^32,33^, COI is not fully universal and is prone to nuclear pseudogenisation^34,35^, and ITS is used mainly in fungi^36^. Primer bias^37,38^ and copy‑number variation persist^39^. Shotgun metagenomics^40^ dispenses with primers, but sequencing libraries are often dominated by prokaryotic and viral reads^41–44^. In practice, we need a marker that is universal across eukaryotes, carries a species-level signal, and is short enough to be retrieved reliably when eukaryotic DNA is scarce^41,43,44^.

A useful precedent stems from metazoan studies, where mitochondrial metagenomics^45^ has been deployed as a PCR-free complement to single-locus barcoding. Current implementations fall into two main modes: voucher-linked workflows, which shotgun-sequence identified specimens and anchor assembled mitogenomes via in silico bait sequences (for example, COI, nad5, rrnL), and bulk-community workflows, which assemble from mixed biomass and infer taxonomic context post hoc through barcode matching and phylogenetic placement. These strategies can perform well when specimen links and dense reference databases are available^46^. Here, we adapt this concept from specimen-centric to environment-centric profiling, emphasizing de novo recovery and stringent post-assembly curation directly from untargeted shotgun data, where eukaryotic DNA is sparse and references incomplete.

We use mitochondrial genomic data to convert standard shotgun sequences into species-level, primer-free inventories, bringing microeukaryote diversity into focus. We scale the approach to whole-ecosystem profiling by (re)assembling 3400 metagenomes from 362 lakes and reservoirs spanning major climatic zones and trophic states across seven continents, curating an atlas of ~20,000 metagenome-assembled genomes (mitoMAGs) resolved into ~3662 species-level representatives. In terms of mitoMAGs counts, the atlas is ~12% larger than RefSeq^47^ r226 (mitochondrial version) and shifts its taxonomic center of gravity: Stramenopiles–Alveolata–Rhizaria (SAR), Cryptophyceae, and Haptista contribute 13,049 entries—a 6.4-fold species increase over their previous species total—with parallel expansions in within-clade breadth (SAR × 6.3, Cryptophyceae × 3.5, Haptista × 1.7).

## Results

### A freshwater mitogenome atlas

**Fig. 1:**
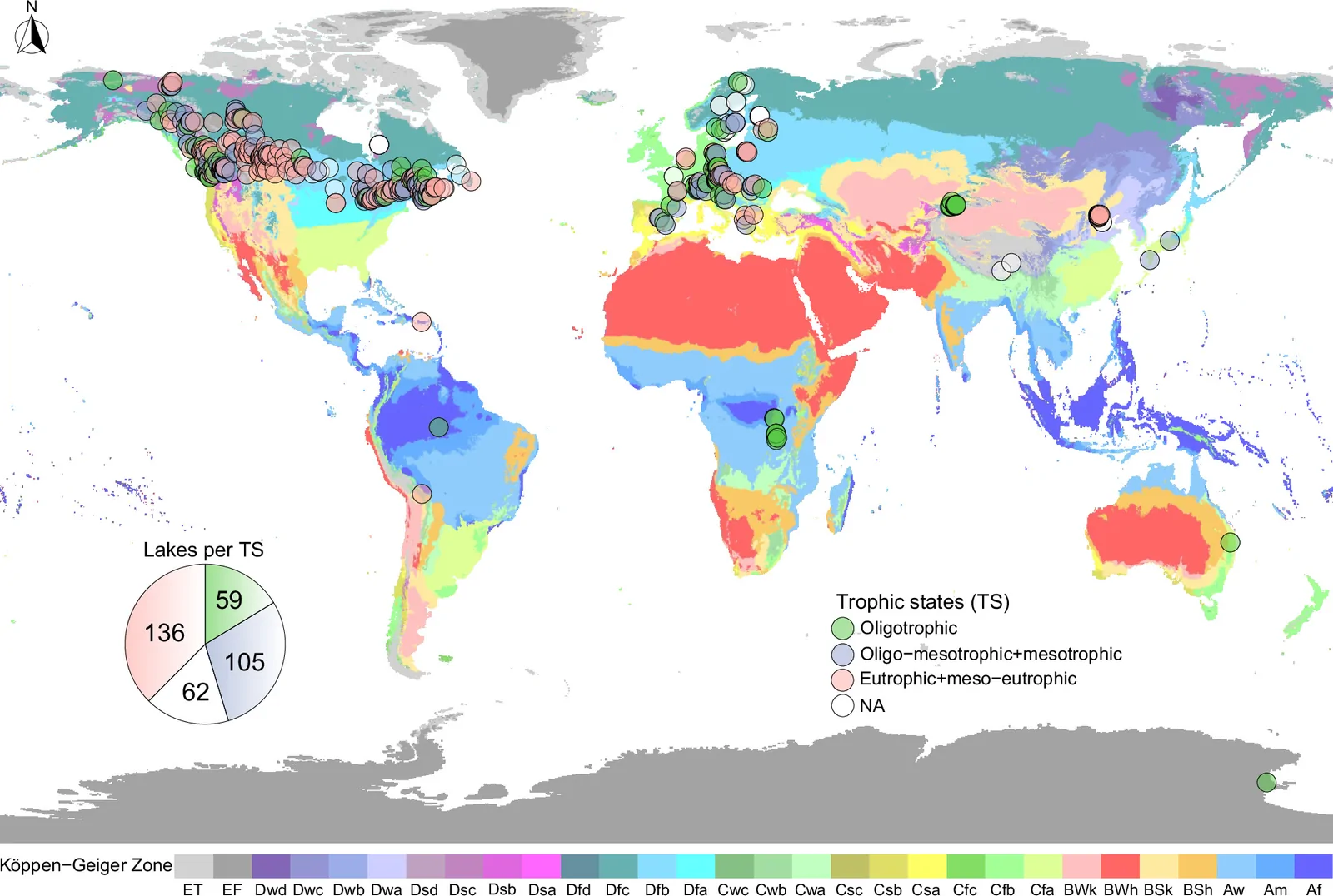
Coverage of the Freshwater Mitochondrial Atlas across Köppen–Geiger climate zones. Locations of lakes and reservoirs contributing mitogenomes are plotted over the Köppen–Geiger climate map (zone legend below). Point fill denotes trophic status (TS): green for oligotrophic; blue for oligo-mesotrophic and mesotrophic; pink for meso-eutrophic and eutrophic; and white for unassigned. The left inset summarizes the number of lakes associated with a specific TS. Zone legend: Af (Tropical rainforest), Am (Tropical monsoon), Aw (Tropical savannah), BWh (Arid desert hot), BWk (Arid desert cold), BSh (Arid steppe hot), BSk (Arid steppe cold), Csa (Temperate dry summer hot summer), Csb (Temperate dry summer warm summer), Csc (Temperate dry summer cold summer), Cwa (Temperate dry winter hot summer), Cwb (Temperate dry winter warm summer), Cwc (Temperate dry winter cold summer), Cfa (Temperate no dry season hot summer), Cfb (Temperate no dry season warm summer), Cfc (Temperate no dry season cold summer), Dsa (Cold dry summer hot summer), Dsb (Cold dry summer warm summer), Dsc (Cold dry summer cold summer), Dsd (Cold dry summer very cold winter), Dwa (Cold dry winter hot summer), Dwb (Cold dry winter warm summer), Dwc (Cold dry winter cold summer), Dwd (Cold dry winter very cold winter), Dfa (Cold no dry season hot summer), Dfb (Cold no dry season warm summer), Dfc (Cold no dry season cold summer), Dfd (Cold no dry season very cold winter), ET (Polar tundra), and EF (Polar frost). Map attribution: The Köppen–Geiger climate-classification dataset of Beck et al. (2023)^112^ (https://www.gloh2o.org/koppen/) is published under a Creative Commons Attribution 4.0 International licence (CC BY 4.0; https://creativecommons.org/licenses/by/4.0/deed.en). Changes were made in preparing this figure: the raster was visualized in R v4.2.2 using the ggplot2, readxl, raster, sf and ggspatial packages; sampling locations, a north arrow, the trophic-status legend and the lake-count inset were added; and the color palette and map annotations were adjusted. Final figure assembly was performed in CorelDRAW 2020. Raw data is provided as a Source Data file.

Drawing on public and newly generated datasets, we assembled a geographically and climatically broad compendium of freshwater shotgun metagenomes. The corpus centers on large lake surveys and is supplemented with site-specific libraries chosen to capture most major climate regimes (Fig. 1). In total, 3400 shotgun libraries from 362 lakes and reservoirs in 28 countries on seven continents were (re)assembled de novo from raw short- and long-read data (Supplementary Data 1 and 2). The set spans oligotrophic to eutrophic productivity states and includes surface and deep layers, different filtration fractions (0.22–5-µm and > 5-µm), and both single-event and multi-season time series (Fig. 1). All assemblies were merged into a contig catalog (~1 Tbp) for uniform downstream analysis. Organellar sequences were retrieved using Tiara, a two-stage deep-learning k-mer-based frequency classifier^48^. Retrained on a large set of prokaryotic, organellar, and nuclear references (“Methods”), the model achieved 99.8% overall accuracy and ~95% recall for mitochondrial contigs in stratified hold-out tests, yielding 80,983 candidate genomic fragments for subsequent downstream curation.

**Fig. 2:**
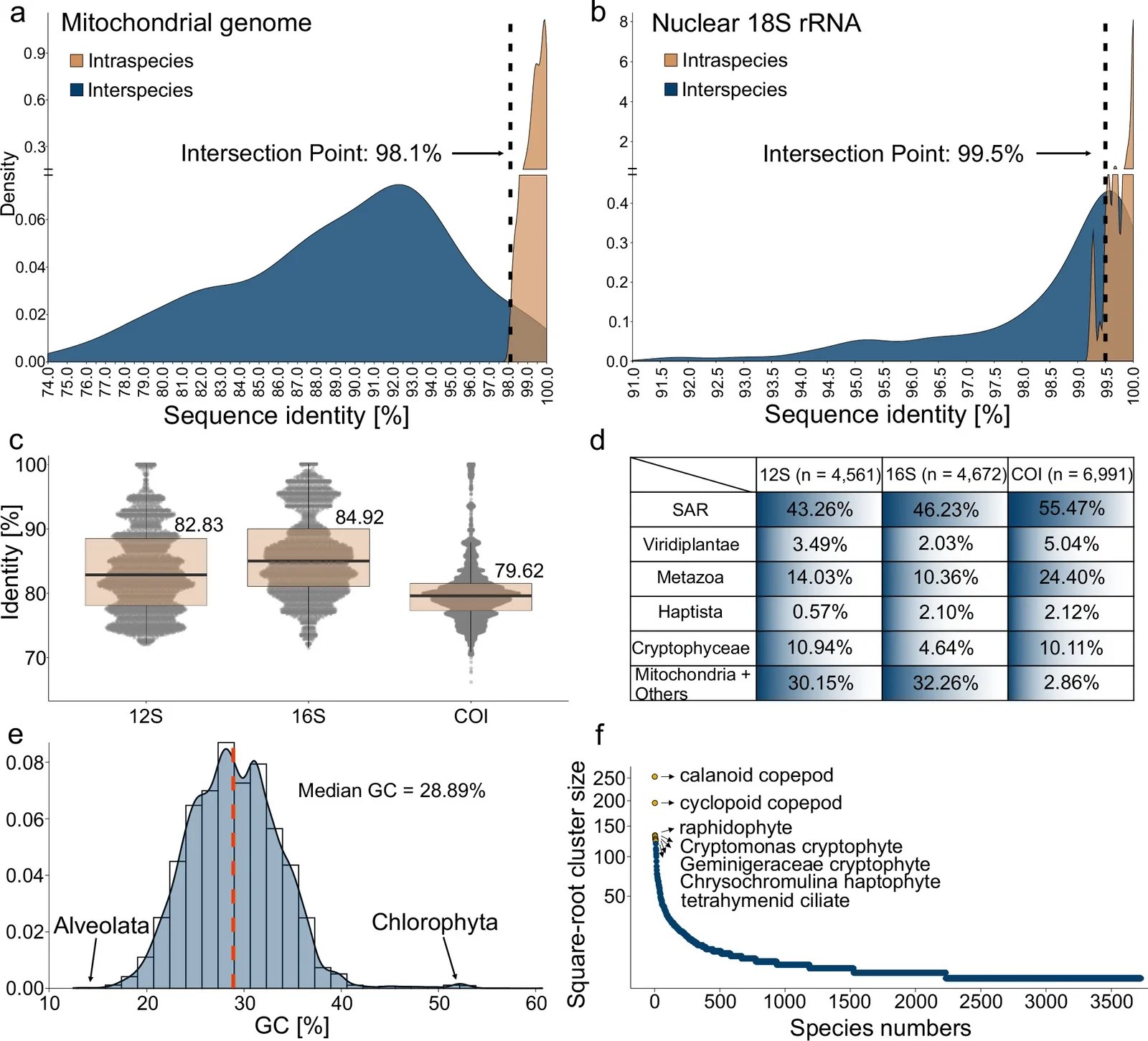
Empirical species-level cutoffs of mitochondrial genomes and FMA properties. **a** Pairwise sequence identity among reference mitochondrial genomes (RefSeq r226). Orange denotes intraspecific pairs (*n* = 91 species); blue denotes interspecific pairs (*n* = 313 genera). The vertical dashed line marks the intersection point of the two distributions at 98.1%, used as the species boundary for mitochondrial clustering. **b** The same analysis for nuclear 18S rRNA (intraspecific *n* = 157 species; interspecific *n* = 104 genera). The intersection point occurs at 99.5%, defining the species-level cutoff for 18S genes. **c** Distribution of identities between FMA-recovered query genes (12S rRNA *n* = 4561; 16S rRNA *n* = 4672; COI *n* = 6991) and reference databases (SILVA v138.1for rRNA and eKOI v1 for COI genes). The central line across the boxplots identifies the median, marking the dataset’s midpoint. The box itself demarcates the interquartile range, extending from the first quartile to the third quartile, encapsulating the central 50% of the data. The whiskers project from the box to the furthest data points not categorized as outliers and show the spread of the main body of the dataset. **d** Taxonomic recovery across the three markers. Bars indicate the proportion of taxa recovered per marker category across the full taxonomic range. **e** GC-content distribution across the 19,925 FMA mitoMAGs. The dashed red line marks the median GC content. **f** Cluster-size distribution relative to species counts. Square-root–transformed cluster sizes plotted against species counts. Taxonomic identities of some of the most abundant clusters are highlighted with arrows. Raw data is provided as a Source Data file.

To set an operational cutoff for species-level clustering, we leveraged the expert-curated taxonomy in the mitochondrial RefSeq^47^ r226. We quantified average nucleotide identity (ANI) in two complementary reference sets. For the between-species distribution, we selected all genera with ≥7 named species (*n* = 313 genera; *n* = 4349 mitogenomes; median = 10 species per genus) present in the nonredundant RefSeq r226 species-representative mitogenomes to compute all within-genus (i.e., between-species) pairwise ANIs (“Methods”, Suppl. Fig. S5). The within-species distribution drew on taxa represented by ≥7 mitogenomes per species in the NCBI Genome database (*n* = 91 species; *n* = 2205 mitogenomes; median = 11 mitogenomes per species), from which we computed all within-species pairwise ANIs (Suppl. Fig. S5). Using the overlap of the two distributions (smoothed using kernel density estimation), we identified the crossover point between within- and between-species similarities at 98.1% ANI (Fig. 2a). Robustness was high: in 30 random subsamplings that retained 99% down to 70% of pairwise comparisons in 1% decrements, the intersection varied minimally (mean 98.1% ± 0.08%). Applied to the entirety of mitochondrial RefSeq r226, this threshold yielded ~92.4% accuracy with extant taxonomy (see Suppl. Information). To evaluate transferability, we further applied the threshold to a microeukaryote-enriched RefSeq r226 subset (482 species). In this subset, 97.9% of mitogenomes remained separated as species-level clusters, supporting the threshold for the microeukaryote diversity currently represented in mitochondrial reference databases. We therefore adopt 98.1% as a taxonomy-anchored, empirical genomic identity boundary for clustering the 80,983 lake-derived candidate mitochondrial genomic fragments at the species level. Pairwise mitogenome identity did not differ between macro- and microeukaryotes, either within (Wilcoxon rank-sum test, Bonferroni correction; *P* = 0.07) or between species (Wilcoxon rank-sum test, Bonferroni correction; *P* = 0.65).

As a reference for a widely used metabarcoding marker, we applied the same density-crossover decision rule to full-length SSU 18S rRNA sequences from the Ribosomal Operon Database (104 genera, 1815 sequences; 157 species, 4634 sequences). The within-species and between-species identity distributions intersected at 99.5%, yielding ~63% species-level concordance across the database (Fig. 2b). We report this value to contextualize our used species clustering cutoff (98.1%).

Applying a 98.1% sequence identity cutoff to the 80,983 candidate contigs yielded 49,894 preliminary species-level clusters, which were further subjected to a conservative, multi-layer curation. First, each cluster was vetted via its representative: protein-coding genes had to be detectable (custom gene-prediction with assigned mitochondrial codes), and representatives lacking detectable gene content were excluded. Second, we identified SSU/LSU rRNA genes with barrnap v0.9 and classified them against a custom SILVA^49^ v138.1 + RefSeq r226 mitochondrial rRNA database, removing representatives whose best matches indicated origin other than mitochondria. Third, we deployed an orthogonal multi-tool classifier panel: viral detection (checkV^50^, VIBRANT^51^), taxonomic profilers (Kaiju^52^, Metabuli^53^), and a deep neural classifier (DeepMicroClass^54^). Any representative called non-mitochondrial (viral, bacterial, plastid/nuclear eukaryote) by ≥2 tools was discarded. This filtering retained 4331 species-level representatives comprising 21,971 members.

Finally, we performed annotation-based taxonomy curation: the proteomes of the cluster representatives were searched with MMseqs2^55^ against a custom mitochondria + bacteria genome taxonomy-centered database (see “Methods”), and labels were assigned by a majority-rule LCA (ties left unclassified). After this step, likely co-fragmented representatives were consolidated through *post hoc* binning and consistency checks, yielding a final resource of 3662 species-level representatives spanning 19,925 mitoMAGs. Of these, 412 showed terminal overlap consistent with circularization, defined as exact overlapping ends of at least 50 bp. Given the diversity of eukaryotic mitochondrial architectures and the variable completeness of metagenomic assemblies, we use mitoMAGs as an operational term for metagenome-derived mitochondrial genome reconstructions, encompassing both complete and, more often, partial records. This high-confidence catalog constitutes the Freshwater Mitochondrial Atlas (FMA) and was used for all downstream analyses.

### A freshwater mitochondrial atlas broadens taxonomic and phylogenetic coverage

Across the 19,925 curated mitoMAGs, recovery spanned the full trophic gradient: 9468 records (47.5%) derived from eutrophic and meso-eutrophic systems, 4415 (22.2%) from oligo-mesotrophic and mesotrophic systems, 5420 (27.2%) from oligotrophic systems, and 622 (3.1%) from lakes lacking a formal trophic assignment (Fig. 1). Collectively, these mitoMAGs represent 3662 species-level clusters and are typically compact, AT-rich, and gene-dense, with a median length of 9.1 kb, median GC content of 28.9%, and median coding density of 69.9%. The catalog contains 265,367 predicted genes, including 4561 12S rRNA, 4672 16S rRNA, and 6991 COI loci. Importantly, these length statistics describe recovered mitochondrial genome-associated reconstructions rather than necessarily complete mitochondrial genomes. At the species-representative level, mitoMAGs ranged from 5.0 to 100.95 kbp, with a median of 15.09 kbp and a mean of 19.15 kbp.

Genome properties differed among major groups and, where comparable RefSeq r226 records were available, provided a reference context for interpreting contiguity and gene recovery. For example, arthropod representatives (*n* = 111) were close to RefSeq r226 arthropod mitogenomes (*n* = 5,956) in median length and protein-coding gene number (15.08 vs 15.59 kbp; 11 vs 12 protein-coding genes), whereas ciliophore representatives (*n* = 571) were shorter and contained fewer predicted protein-coding genes than available RefSeq r226 ciliophore mitogenomes (*n* = 20; 16.28 vs 47.14 kbp; 15 vs 41 protein-coding genes). This contrast indicates that some FMA representatives correspond to compact, near-reference-length mitochondrial genomes, whereas others capture shorter gene-bearing portions of larger or structurally complex mitochondrial genomes. Full group-level summaries of size, GC content, coding density, gene content, and RefSeq comparisons are provided in Supplementary Table S1.

**Fig. 3:**
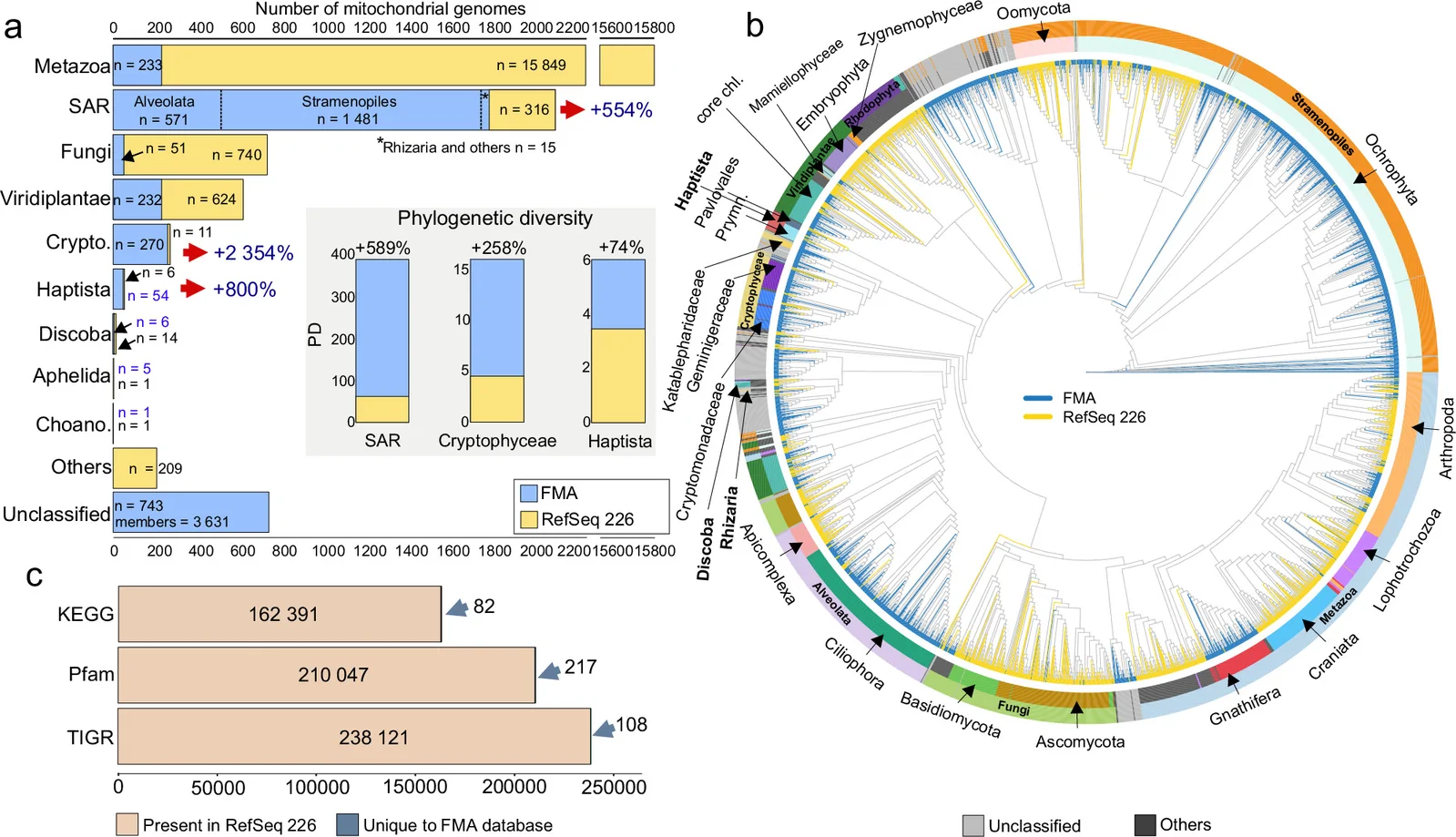
Phylogenetic, taxonomic, and functional census of the Freshwater Mitochondrial Atlas (FMA). **a** Species-representative mitoMAGs recovered in this study (*n* = 3662; blue) versus RefSeq r226 (yellow), grouped by major clades (counts shown within bars). FMA expands taxonomic breadth markedly (e.g., SAR + 554%, Cryptophyceae +2354%, Haptista +800% in species representatives). Inset, Faith’s phylogenetic diversity (PD) on the species tree shows parallel gains (SAR + 589%, Cryptophyceae +258%, Haptista +74%). **b** Maximum-likelihood phylogeny of mitochondrial COI genes (IQ-TREE, LG + C10 + F + R10), comprising RefSeq r226 mitogenomes (yellow; *n* = 1411) and FMA mitoMAGs (blue; *n* = 1826). Concentric rings (outer → inner) show taxonomy. Taxonomic ranks with low representation were grouped into a single category (gray), and unclassified mitoMAGs are shown in dark green. RefSeq r226 Metazoa were subsampled to improve visualization. **c** Functional annotation summary for FMA across KEGG, Pfam, and TIGRFAMs. Orange segments indicate annotations also present in RefSeq r226; blue arrows mark FMA-unique annotations. Raw data is provided as a Source Data file.

Annotation-based classification of species-level clusters—using proteome annotation, triangulated with rRNA and COI assignments—showed substantial taxonomic gains over RefSeq r226, particularly in Cryptophyceae (+2382%), Haptista (+800%), and SAR (+571%) (Fig. 3a). At the same time, lower-rank taxonomic resolution remained limited. High-identity searches of recovered 12S rRNA, 16S rRNA, and COI genes against mitochondrial marker databases linked 855 of 19,925 mitoMAGs to named genera at ≥98% identity, corresponding to 4.3% of the atlas. Notably, 748 FMA clusters could not be confidently assigned to any taxonomic rank. Together, these patterns show that the FMA expands coverage most strongly in portions of microeukaryotic diversity that remain poorly represented in current mitochondrial reference databases.

The added genomic breadth translated into deeper within-clade structure: clade-specific phylogenies (see “Methods”) revealed large increases in Faith’s phylogenetic diversity (PD) for SAR ( + 529%), Cryptophyceae (+254%), and Haptista (+74%) relative to RefSeq r226 (Fig. 3a). To further visualize phylogenetic and taxonomic gains relative to existing resources, we inferred a maximum-likelihood phylogeny from COI genes for FMA species representatives (*n* = 1826 mitoMAGs) along with a balanced subset of RefSeq r226 (*n* = 1411 mitogenomes; “Methods”), under a IQ-TREE, LG + C10 + F + R10 model to accommodate empirical amino-acid frequencies, across-site compositional heterogeneity (C10 mixture profiles), and among-site rate heterogeneity modeled via a 10-category FreeRate model (Fig. 3b). To complement this analysis, we additionally inferred a phylogeny from concatenated 12S and 16S rRNA genes for FMA species representatives (*n* = 1542 mitoMAGs) and RefSeq r226 (*n* = 6924 mitogenomes) (Suppl. Fig. S1). Inclusion of FMA representatives markedly increased coverage at deeper nodes—particularly in undersampled microeukaryote lineages such as SAR and Cryptophyceae—filling conspicuous gaps in current mitochondrial references (Fig. 3b). Thus, the FMA contributes not only more taxa from undersampled groups, but also substantially greater evolutionary spread within them.

As an independent check on database fidelity, we compared genome-wide annotation profiles between FMA and RefSeq r226 across TIGR^56^, KEGG^57^, and Pfam^58^ annotations. Nearly all markers present in FMA were also present in RefSeq r226 (TIGR; 99.95%; Pfam: 99.89%; KEGG: 99.94%), with only a marginal FMA-unique fraction (TIGR: 0.05%; Pfam: 0.11%; KEGG: 0.06%). Dominant annotations mapped to canonical mitochondrial *OXPHOS* genes (e.g., *NADH* dehydrogenase and cytochrome-c oxidase subunits; Suppl. Fig. S2a), with higher annotation rates for TIGRFAMs and moderate for KEGG (TIGR: 89.8% annotated; KEGG: 61.6% annotated; Suppl. Fig. S2b). These concordances indicate that FMA recapitulates the established mitochondrial gene complement with no systematic evidence of non-mitochondrial carryover, supporting mitochondrial origin and resource robustness. Among the rare FMA-unique signals, we identified a recurrent candidate non-canonical mitochondrial locus in Geminigeraceae, a cryptophyte family represented in the atlas by 1079 mitochondrial fragments and 96 species-level representatives. The locus, present in 14 curated mitoMAGs, encoded a type I restriction–modification methyltransferase domain fused to an HsdS target-recognition domain, potentially coupling DNA methylation with sequence-specific recognition, and was adjacent to a transposase-like ORF. Its absence from current mitochondrial references and mobile-element-like architecture make it a representative example of how the FMA can reveal rare mitochondrial gene-content novelties, beyond expanding taxonomic and phylogenetic coverage.

### Unclassified mitoMAGs occupy undersampled branches of the eukaryotic tree

**Fig. 4:**
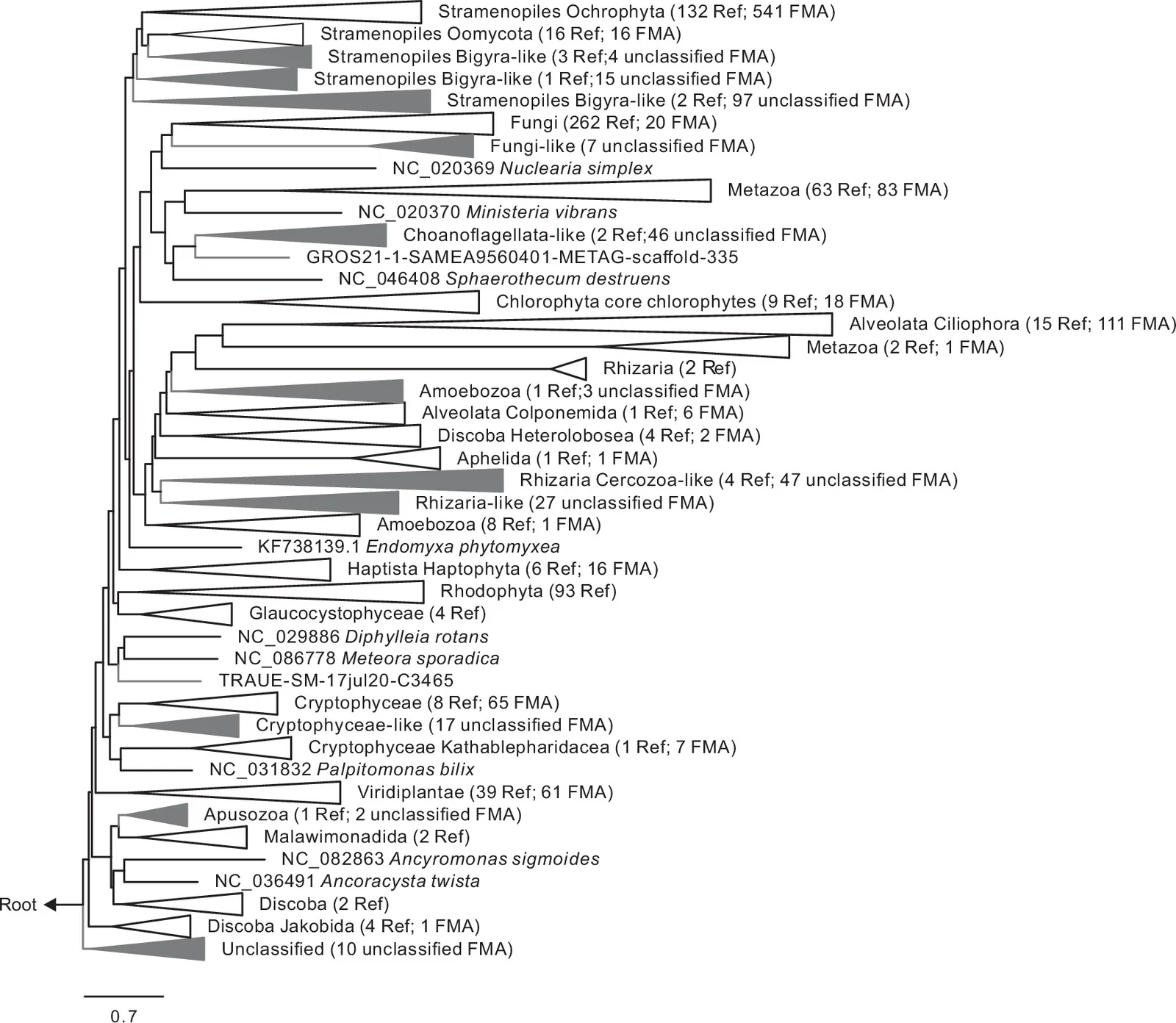
Phylogenomic placement of unclassified FMA mitoMAGs. Maximum-likelihood mitochondrial phylogeny inferred from a concatenated amino-acid alignment of 24 conserved mitochondrial marker genes. The dataset comprised dereplicated FMA mitoMAG representatives and a genus-dereplicated reference collection including RefSeq r226, additional SAR mitogenomes from NCBI, and selected published protist mitogenomes. Genomes represented by fewer than seven recovered mitochondrial markers were excluded. Alignments were refined, trimmed, filtered to remove the fastest-evolving sites, and analysed in IQ-TREE using a PMSF approximation with the LG + C60 + F + G model. Bacterial genomes were included as outgroups for rooting. Collapsed wedges denote major clades; labels indicate the number of reference genomes and FMA representatives in each clade. Gray wedges and labels mark clades containing FMA representatives that remained unclassified after annotation-based taxonomic classification. Scale bar, amino-acid substitutions per site.

To further resolve the unclassified component of the atlas, we placed dereplicated FMA representatives and genus-dereplicated references in a phylogeny inferred from conserved mitochondrial markers (Fig. 4). Unclassified mitoMAGs were not distributed as a diffuse tail of the catalog. Instead, they fell into recurrent phylogenetic neighborhoods adjacent to reference-poor branches, with prominent placements near Bigyra-like stramenopiles, Rhizaria/Cercozoa-like branches, Cryptophyceae-like clades, and choanoflagellate- or fungal-like lineages. Smaller components were associated with Amoebozoa- and Apusozoa-like clades. We interpret these placements conservatively as phylogenetic affinities rather than formal taxonomic assignments, and therefore retain these records as unclassified in the atlas. Nevertheless, the analysis shows that much of the unclassified FMA fraction is evolutionarily structured and anchored to recognizable, undersampled regions of eukaryotic mitochondrial diversity. Thus, the FMA expands reference space in two complementary ways: by densifying named freshwater clades, and by exposing coherent mitochondrial lineages for which current databases still lack sufficient taxonomic resolution.

### Assessing diversity through the mitochondrial lens

**Fig. 5:**
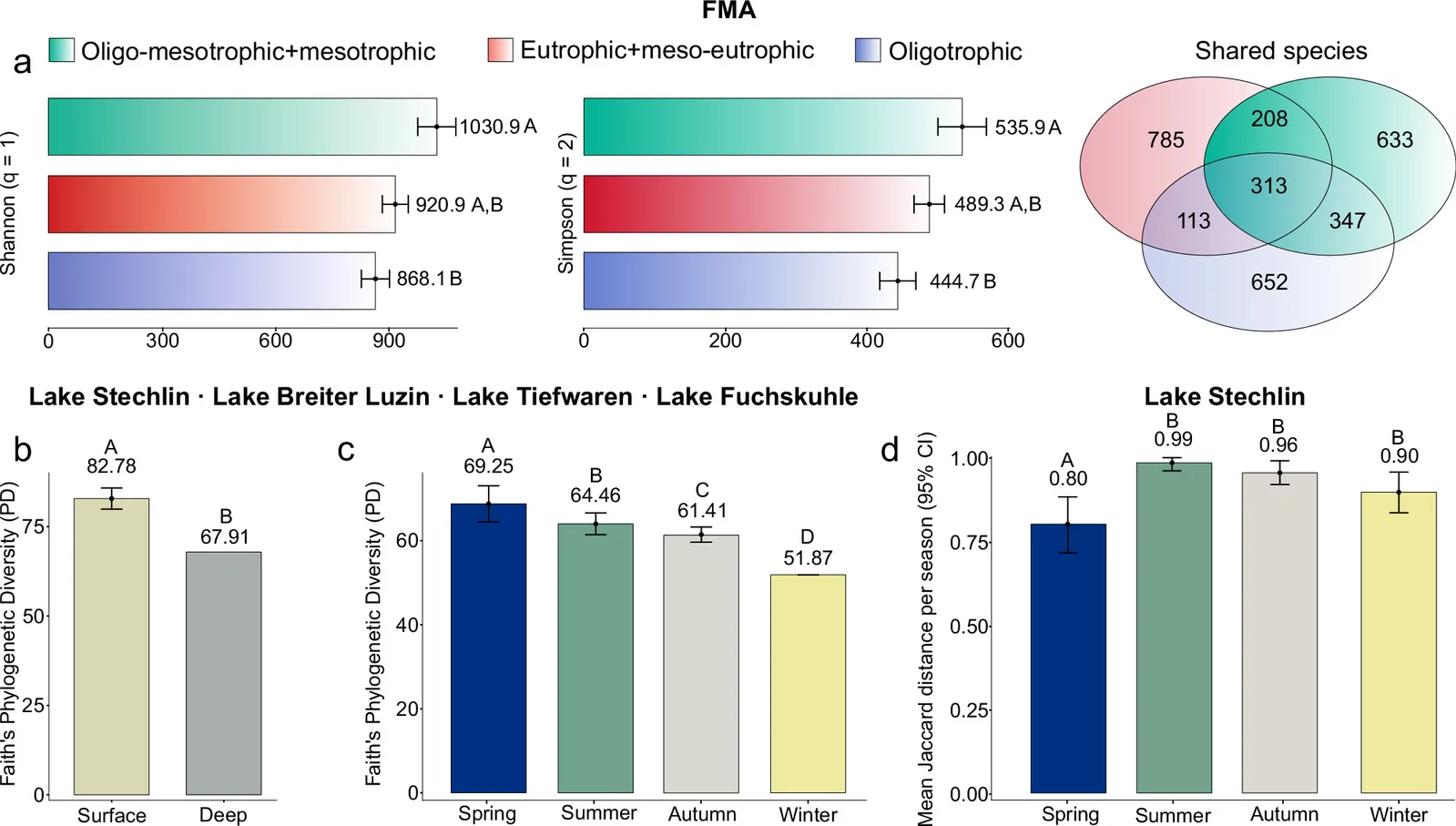
MitoMAG-informed diversity across trophic status, seasons and depths. **a** Coverage-standardized Hill diversity at a common sample coverage of 75.8% across the FMA trophic classes: combined oligo-mesotrophic and mesotrophic (green), combined eutrophic and meso-eutrophic (red), and oligotrophic (blue). Shannon diversity is expressed as the effective number of species ((*q* = 1); left), and inverse Simpson diversity as the effective number of species ((*q* = 2); center). Bars indicate coverage-standardized point estimates calculated using iNEXT; error bars show 95% confidence intervals obtained from 1000 bootstrap resamples. The Venn diagram (right) shows the overlap in species representatives among trophic classes; a representative was assigned to a class when at least one member of mitoMAG was recovered from that class. Differences among trophic classes were evaluated using Kruskal–Wallis rank-sum tests (Shannon-Kruskal–Wallis (H = 249.44), d.f. = 2, (*P* < 2.2e-16); Simpson-Kruskal–Wallis (H = 222.77), d.f. = 2, (P < 2.2e-16)) followed by two-sided Dunn’s pairwise tests with Bonferroni adjustment for multiple comparisons. **b**, **c** Rarefied Faith’s phylogenetic diversity (PD) of metazoan-free microeukaryote communities from four German lakes. **b** Comparison between surface and deep-water communities, rarefied to 944 genomes (Kruskal–Wallis (H = 1713.4), d.f. = 1, (*P* < 2.2e-16)). **c** Comparison among spring, summer, autumn and winter communities, rarefied to 580 genomes (Kruskal–Wallis (H = 3198.3)), d.f. = 3, (*P* < 2.2e-16). PD was calculated on fixed species trees inferred from 64 mitochondrial markers. Bars show mean PD across 1000 rarefaction draws; error bars show the corresponding 95% quantile intervals, defined by the 2.5th and 97.5th percentiles. **d** Mean Jaccard distance between epilimnion and hypolimnion communities by season for paired sampling dates in Lake Stechlin. Bars show seasonal means for spring (*n* = 31), summer (*n* = 12), autumn (*n* = 24) and winter (*n* = 10); error bars show 95% bootstrap confidence intervals obtained from 1000 resamples of the date-level distances. Lower values indicate greater similarity between depth layers. Seasonal differences were detected using a Kruskal–Wallis rank-sum test ((H = 13.46), d.f. = 3, (*P* = 0.003)), followed by two-sided Dunn’s pairwise tests with Bonferroni adjustment for multiple comparisons. Kruskal–Wallis tests are omnibus tests; therefore, one-versus two-sided specification does not apply. Capital letters indicate the results of Dunn’s tests; groups not sharing a letter differ significantly at adjusted (*P* < 0.05). Test statistics and exact pairwise adjusted (*P*) values are reported in Supplementary Data 10. Source data are provided with this paper.

To test whether mitochondrial inventories recover the classical productivity–diversity pattern in lakes, we grouped 19,303 of 19,925 mitoMAGs with trophic metadata into oligotrophic, intermediate (oligo-mesotrophic + mesotrophic), and nutrient-rich (meso-eutrophic + eutrophic) classes. Within each class, genomes were clustered at the empirically derived 98.1% genome identity cutoff; cluster counts define species numbers in that class, and cluster membership (mitoMAGs per cluster) serves as a proxy for their incidence. To offset uneven sampling effort across classes, we used coverage-based rarefaction (iNEXT^59^) to a common 75.8% sample coverage and report Hill numbers as effective species. At shared coverage, lakes at intermediate trophic level were most diverse by both indices: Shannon (*q* = 1) effective species 1031 versus 921 (nutrient-rich) and 868 (oligotrophic); Simpson-Gini (*q* = 2) 536 versus 489 and 445, respectively (Fig. 5a). Bootstrap 95% CIs from 1000 replicates support this ranking, with partial overlap for the two productivity richer classes (Fig. 5a). Filter-size effects were negligible: diversity at shared coverage showed only very weak correlations with filter size (relationship was quantified by computing Pearson correlations between filter size, as a numeric variable, and coverage-standardized Shannon and Simpson-Gini; Shannon *r* = −0.088, *P* = 9.2 × 10^−11^ *n* = 5385; Simpson-Gini *r* = −0.077, *P* = 1.4 × 10^−8^, *n* = 5385), indicating that patterns are robust across the ≥5 µm and 0.22–5 µm fractions. Together, these results point towards a mesotrophic peak in diversity.

Having established the across-lake pattern, we next asked whether mitoMAGs' inventories capture phenology within a shared climatic setting. 843 metagenomes from four German lakes—Breiter Luzin (*n* = 108), Fuchskuhle (*n* = 381), Stechlin (*n* = 233), and Tiefwaren (*n* = 121) were selected for long time series, common regional climate, and contrasting characteristics. Together they contributed 4615 mitoMAGs (23.16% of FMA) and provided paired surface–deep sampling across size fractions ( ≥ 5 µm, 0.22–5 µm) and seasons. The set spans a pronounced trophic gradient, from oligo-mesotrophic Stechlin (*n* = 1019 mitogenomes), through intermediate Breiter Luzin (*n* = 457 mitogenomes) and Tiefwaren (n*n* = 639 mitogenomes), to nutrient-rich, dystrophic Fuchskuhle (*n* = 2500 mitogenomes). Unless noted, from here on, the analyses used a metazoan-free subset (see “Methods”); species units followed the 98.1% genome identity definition, with one representative per cluster placed on a fixed microeukaryote tree (64 mitochondrial markers; Methods). Seasonal diversity did not differ by trophic state at standardized coverage (*P* = 1.00; “Methods”). The lake selection enabled matched, like-for-like comparisons of phenology and depth across trophic states.

Because PD is defined on species sets, we equalized effort by sample-size rarefaction to the smallest mitoMAGs count (seasons: 580; depths: 944). We drew 1000 rarefied sets for each stratum, collapsed to unique species, mapped the set onto the fixed tree, and recorded Faith’s PD (sum of branch lengths). To separate phylogenetic structure from richness, we computed the standardized effect size of PD (SES-PD) using richness-matched randomizations from the lake-wide microeukaryote pool on the same tree (1000 null draws per stratum); negative SES-PD indicates phylogenetic clustering (Supplementary Data 10). We observed parallel gradients in time and depth. Seasonality: PD declined from spring → summer → autumn → winter (68.85 → 63.99 → 61.34 → 51.87; spring–winter Δ = +16.98, +32.7%), while SES-PD was negative throughout and became more negative toward winter ( − 0.38, −0.38, −0.70, −3.38). Depth: PD was higher in the surface than in the deep layers (82.88 vs 67.91; Δ = +14.97, +22.0%), with more negative SES-PD at depth (−1.10 vs. −1.63). Thus, at equal richness, spring and the surface layer span a broader portion of the evolutionary tree, whereas winter and the deep layer draw from a narrower span (Fig. 5b, c).

In a single lake ecosystem—Lake Stechlin, the deepest (~69.5 m) and largest (4.3 km^2^) of the four, we quantified surface–deep layers connectivity using a metazoan-free time series (2012–2020; 204 metagenomes: 137 surface, 67 deep; 102 at 0.22 µm and 102 at ≥5 µm). Mapping shotgun reads to species-representative mitoMAGs, we considered a species present when coverage was ≥2× and breadth ≥50% of its genome (“Methods”), yielding detection–abundance tables for 241 species-level clusters (defined at 98.1% genomic identity). For between-layer comparisons, we used paired dates within each season—on the same day when available, otherwise the nearest date within a month—and computed Jaccard distance based on presence–absence for each pair (*n* = 77 seasonal pairs: spring 31, summer 12, autumn 24, winter 10; covering 1382 mitogenome detections). Surface–deep distances were lowest in spring and highest in summer (spring 0.802, 95% CI 0.716–0.883; summer 0.985, 0.961–1.000; autumn 0.955, 0.920–0.991; winter 0.897, 0.836–0.957; bootstrap CIs) (Fig. 5d). Overlap counts agreed: the fraction of shared species was 44.5% in spring (77/173), 32.3% in winter (30/93), 14.6% in autumn (15/103) and 5.4% in summer (7/129). Thus, within this lake, the surface and deep layers’ microeukaryote communities are most similar in spring and most distinct in summer.

## Discussion

Microeukaryote ecology lacks a species-resolved, phylogenetically explicit scaffold that is marker-agnostic (18S, COI, ITS, etc.) and reports biodiversity in common, standardized units, making results comparable within and across surveys^32,33,35,37,38^. Microscopy, though foundational, undercounts and under-resolves species^60^; single-locus barcodes transformed detection, biogeography^4^ and biotic-interaction mapping (predator–prey, host–parasite, symbioses), yet comparability breaks down because different microeukaryote lineages and studies rely on different loci and regions (18S V4 vs V9; COI fragments; ITS1 vs ITS2)^37^, different primer sets, and different similarity thresholds^34^ and pipelines^61^—yielding estimates in incompatible currencies^37^.

MitoMAGs can provide a primer-free scaffold for species-resolved, evolution-aware ecology^45,62,63^. Multigene signal yields species-level units and places them on a common phylogeny—a shared coordinate system for community data. This makes inventories portable and interpretable: not only how many taxa occur, but where they sit on the tree (clustering vs overdispersion) and how lineage breadth shifts across space, time, and ecosystem state^64^. In this framework, phylogenetic clustering and overdispersion describe the architecture of diversity on the tree. Clustering indicates that species-level units are concentrated within closely related lineages, whereas overdispersion indicates that they span more distant branches. This matters because richness can remain unchanged while the evolutionary composition of a community is reorganized: turnover may occur among near relatives within the same lineage pool, or through replacement and recruitment across deeper regions of the eukaryotic tree. The latter represents a deeper form of community change, because it alters not only how many species-level units are present, but which parts of eukaryotic evolutionary history are represented. The phylogenetic scaffold, therefore, allows the FMA to detect phylogenetic homogenization, expansion, or turnover that would be invisible from species counts alone.

Because barcodes (OTUs/ASVs) and image-based units are typically defined in a study-specific way (shaped by sampling design, analytical pipeline, and observer bias), they do not themselves constitute interoperable identifiers across datasets^37^, even when mapped to shared reference databases. By contrast, individual mitoMAGs are concrete, globally addressable genomic objects that can anchor comparisons across methods, studies, and habitats. Amplicons remain the workhorse for breadth and speed^4^, while mitoMAGs can supply the stable reference that aligns studies and links composition to mechanism. In microeukaryotes, rich in phylogenetic^63^ and functional diversity^6^ yet under-resolved at the species level, each added mitoMAGs converts a sample-bound label into a reusable species entity on the same tree, materially improving comparability and inference without dependence on primer choice or observer criteria.

Mitochondrial metagenomics has a strong foundation in metazoan biodiversity^45,65^ research, but its environmental application remains comparatively recent. Most studies have recovered mitochondrial reads or genomes from shotgun sequencing of bulk organismal assemblages^46,65,66^, taxon-enriched mixtures^67^ or specimen-associated material^68^, rather than from untargeted environmental metagenomes. The metazoan representatives recovered here therefore fall within this emerging field, but arise as non-target recoveries from freshwater community DNA, consistent with contributions from small aquatic animals, planktonic life stages, eggs, tissue fragments or extracellular DNA retained during sampling. For microeukaryotes, by contrast, mitochondrial recovery has largely advanced through single-cell genomics^69,70^, which has demonstrated feasibility but not broad ecosystem coverage. The present study connects these trajectories by applying de novo mitochondrial genome recovery to total freshwater community DNA, with microeukaryotic diversity as the primary focus.

The Freshwater Mitochondrial Atlas extends organellar recovery to the ecosystem scale: across 362 lakes and ~20,000 mitoMAGs, it densifies precisely the thinnest portion of reference space (SAR, Cryptophyceae, Haptista), enabling species calls and placements to be compared across datasets without study-specific tuning. Species boundaries follow an empirical, taxonomy-anchored rule (98.1% genome identity; see Suppl. Information) derived from within- versus between-species mitogenome identity; applying an analogous decision rule to full-length 18S rRNA genes yields a crossover near 99.5% but only ~63% species-level concordance—consistent with known limits of single-locus barcodes^32^ and the finer granularity of multigene mitogenomes^63^. Phylogenetic breadth increases commensurately, converting a sparse scaffold into a workable common backbone.

Read on this backbone, freshwater diversity patterns at the species level broadly align with coexistence theory^71^. Across the trophic gradient, diversity tends to be highest at intermediate productivity, with the clearest differences in Shannon rather than Simpson-Gini diversity. This indicates that mesotrophic communities support a richer and more even assemblage of non-dominant taxa, whereas oligotrophic and eutrophic systems are comparatively more dominance-skewed. These trends are qualitatively consistent with productivity–diversity, and intermediate-disturbance frameworks^72,73^, in which low-resource systems are constrained by strong environmental filtering and high-resource systems by the paradox of enrichment and competitive exclusion by bloom-formers. The overall pattern echoes reports for microeukaryotes and phytoplankton, where intermediate trophic states frequently—but not invariably—maximize diversity^74–77^.

Within a shared climatic regime, season and depth follow canonical assembly dynamics^78^. In spring, turnover, rising light, temperature, and re-oxygenation reopen niches, and surface water microeukaryote communities span more of the phylogeny (higher PD; weaker clustering). In winter, and in the deeper waters, assemblages comprise closer relatives under stronger environmental filtering (more negative SES-PD). Metacommunity theory^79^ interprets the spring convergence of surface and deep layers as a mass-effects phase under high vertical connectivity, followed by summer divergence driven by species sorting along light–temperature–oxygen gradients and physical isolation^80^. Convergent evidence from an independent, lake-centered, high-frequency time series in a temperate reservoir recapitulates this vernal/estival sequence^81^.

From eKOI COI gene classifications, only ~11.2% of sequences exceeded 85% identity to reference databases (Fig. 2c), indicating that most COI-bearing freshwater mitoMAGs are distant from currently represented mitochondrial diversity. The phylogenomic placement of unclassified representatives sharpened this view. Rather than forming a diffuse residual category, many unclassified mitoMAGs occupied recurrent neighborhoods adjacent to reference-poor branches of the eukaryotic tree, including Bigyra-like stramenopiles, Rhizaria/Cercozoa-like branches, Cryptophyceae-like clades and choanoflagellate- or fungal-like lineages. We interpret these placements as phylogenetic affinities rather than formal taxonomic assignments, but they show that lack of a taxonomic name often reflects insufficient reference density rather than absence of evolutionary signal. Together with classified representatives assigned to ochrophytes, ciliates, rhizarians, cryptophytes, haptists, fungi, heterotrophic flagellates, rotifers and microcrustaceans, these lineages show that the FMA expands mitochondrial representation across multiple compartments of freshwater food webs, from phototrophs and mixotrophs to grazers, parasites, saprotrophs and zooplankton. Their divergence from named references likely reflects limited mitochondrial sampling of freshwater microeukaryotes, scarce cultivated or single-cell genomic representation for many planktonic taxa, and rapid sequence evolution of eukaryotic mitochondrial genomes. Thus, the FMA broadens mitochondrial coverage in two complementary ways: it densifies named freshwater clades and anchors previously unclassified environmental mitoMAGs to undersampled regions of eukaryotic history.

Nevertheless, this annotated fraction was sufficient to reconstruct a clade-level, mitogenome-resolved view of lake ecosystem structure. Across continental freshwater lakes, mitogenomes resolve a trophic web organized around a recurrent core of primary producers, mixotrophs, and consumers. Cosmopolitan green algae in Chlorellaceae and Pleurochloridaceae, together with cryptophytes and related flagellates in Cryptomonadaceae and Geminigeraceae, form a planktonic photosynthetic scaffold that recurs across a broad Northern Hemisphere longitudinal gradient (Asia–Europe–North America). This basal layer is complemented by diverse ochrophyte (photosynthetic stramenopile) primary producers and mixotrophs, together with haptophytes and cryptophytes, including chrysophyte relatives in Dinobryaceae and haptophyte-like Chrysochromulinaceae, as well as pennate and centric diatoms in Fragilariaceae, Skeletonemataceae, and Thalassiosiraceae. Mitogenomic affinities show that these families co-occur across continents and climatic zones, consistent with a globally conserved “core phytoplankton module” that underpins lake productivity. Superimposed on this phototrophic matrix are heterotrophic and phagotrophic lineages that likely couple primary production with higher trophic levels via grazing-mediated carbon transfer. Ciliate lineages (phylum Ciliophora), recovered across a pan-Northern Hemisphere lake gradient, constitute a pervasive microzooplankton compartment of the pelagic food web, channeling bacterial and nanophytoplankton production into protozoan biomass and regenerating dissolved nutrients that sustain primary producers. Alongside these, apoikiacean flagellates (Apoikiospumella-like) likely exploit bacterial and picoalgal prey, while rotifers in Synchaetidae integrate nanoplankton, microalgae, and protozoa into higher trophic levels. Oomycete parasites in Pythiaceae and Saprolegniaceae overlay this network with a cross-kingdom infection web that can re-route carbon from living phytoplankton, ciliates, and metazoans into detrital pathways.

Taken together, the atlas brings microeukaryote diversity into operational focus: a species-resolved inventory anchored to a shared phylogeny, yielding comparable, transferable metrics across datasets. Sensitive to community structure and phenology yet scalable from single lakes to continents, it recovers canonical macroecological patterns, surfaces tractable mechanistic leads (e.g., a candidate domesticated restriction–modification module), and broadens the portion of eukaryotic history accessible to environmental genomics. The result is a primer-free, tree-aware accounting of composition and lineage breadth that can support cross-study inference and long-term ecological surveillance^64^; it will strengthen iteratively as sampling, references, and joint organellar–nuclear analyses expand.

## Limitations

Several caveats warrant consideration. Mitochondrial genomes provide a powerful organellar window onto microeukaryotic diversity, but they do not capture all dimensions of eukaryotic biology. Mitochondrial and nuclear histories can diverge; mitochondrial copy number, genome size, and architecture vary among clades; and some anaerobic protists harbor mitochondrion-related organelles^82^, such as hydrogenosomes or mitosomes or have secondarily lost canonical mitochondria^83–85^, and may therefore be missed by standard mitochondrial recovery. Atypical architectures, including kinetoplastid minicircles, diplonemid multipartite genomes, and lice-like minichromosomes^86^, are also likely to be under-recovered by the assembly and filtering strategy used here. Geographic coverage remains uneven, reflecting both the availability of deeply sequenced freshwater metagenomes and the underlying distribution of lake systems. Global lake inventories show a strong Northern Hemisphere concentration, especially across formerly glaciated regions of North America and Eurasia^87^. The FMA should therefore be viewed as a global-scale but non-exhaustive resource: it includes tropical and Southern Hemisphere systems, while making clear that these regions remain priorities for future expansion.

In our framework, cluster size should be viewed as an assembly-conditioned measure of recoverable mitochondrial signal, rather than as a direct estimate of cell abundance, biomass, mitochondrial copy number, or nuclear genome abundance. Several biological and technical processes can decouple mitochondrial recovery from organismal abundance: copy number can vary among taxa and physiological states; genome size, structure, and fragmentation can influence read recruitment and assembly; rare taxa may not reach the coverage required for reliable recovery; and, as in genome-resolved metagenomics more broadly, assembled sequences represent population-level consensus genome fragments rather than individual clonal lineages. Individual-level mitochondrial variation represents an additional caveat. Heteroplasmy, microchimerism, within-population polymorphism and the co-occurrence of closely related lineages can all introduce mitochondrial sequence heterogeneity into environmental assemblies. In the FMA, these signals are partly absorbed by consensus assembly and species-level clustering, and are therefore expected to affect mainly fine-scale haplotype structure rather than broad species-level or phylogenetic patterns. They remain relevant for interpreting rare high-divergence profiles and local sequence variation, but are unlikely to explain the large reference divergence or the major diversity gradients reported here.

Consequently, low-abundance species are likely to be under-recovered, and recovered mitogenomes may be partial, particularly for lineages with unusual organellar architectures or limited representation in mitochondrial reference databases. Potential over-splitting of non-overlapping mitochondrial fragments was minimized in the FMA before downstream analyses through *post hoc* curation based on tetranucleotide composition, differential coverage, taxonomic consistency, and non-redundant mitochondrial gene content. This procedure captures the most detectable cases of fragmentation-driven over-splitting, although residual cases may remain for rare or low-coverage lineages that cannot be robustly evaluated by coverage-based binning. Such cases could lead to a slight overestimation of species counts, but are unlikely to drive the broad diversity patterns reported here. Fragmentation may also affect absolute phylogenetic diversity estimates: shorter fragments can retain sufficient signal for phylogenetic placement, but may capture fewer lineage-specific divergent sites than longer fragments, making branch-length and PD estimates potentially conservative in some cases.

Completeness is best interpreted in relation to the inference being drawn. Complete mitogenome assemblies remain necessary for resolving genome architecture, gene order, and full organellar gene repertoires. The FMA analyses, however, were designed around curated mitoMAGs signal rather than complete mitochondrial genomes in every case. Species-level units were defined from fragments with sufficient mitochondrial overlap to satisfy the 98.1% identity and coverage criteria, and were retained only after mitochondrial gene-content validation, contamination filtering, and *post hoc* checks for fragmentation-driven over-splitting. Phylogenetic analyses used representatives carrying the relevant marker genes, whereas ecological analyses relied on standardized recovery of curated species-level units across trophic states, seasons, and depths. Under this framework, partial but well-curated mitoMAGs can support species-level clustering, phylogenetic placement, and comparative ecological inference, while complete assemblies remain essential for questions concerning mitochondrial architecture, genome organization, and full gene-content evolution.

We interpret cluster size and absolute PD values with caution, placing emphasis on standardized comparisons across trophic states, seasons, and depths rather than on the absolute number of recovered fragments. Coverage-based rarefaction, bootstrap resampling, sample-size rarefaction, and null models reduce the influence of uneven recovery effort, but they cannot eliminate lineage-specific differences in mitochondrial architecture or assembly probability. Future long-read metagenomics, organelle-enrichment strategies, and targeted sampling of underrepresented taxa and atypical lineages should improve contiguity and expand the range of mitochondrial architectures recovered. As reference coverage grows, mitogenome-based ecology should increasingly shift from an assembly-intensive discovery phase toward a more routine read-mapping framework, in which shotgun reads from new samples can be interpreted against curated mitochondrial atlases to quantify species-level composition, turnover, and phylogenetic structure.

## Methods

### Samples collection

A total of 3400 freshwater shotgun metagenomes were generated by authors or retrieved from publicly available repositories (data freeze 2025.01.01; see Supplementary Data 1 and 2), encompassing 362 freshwater bodies across 28 countries (median of 4 lakes/country; 16 metagenomes/country), seven continents (i.e., Europe, Africa, Antarctica, Asia, North America, South America, and Oceania) and major climatic zones, although sampling density remained uneven among regions. The dataset integrates large-scale environmental sequencing efforts, including habitat-focused studies, which comprise time-series samples from surface and deep layers of four German lakes (LIMNOS dataset), alongside additional initiatives (e.g., PELAGICS, LakePulse), spanning a wide range of climatic and trophic contexts^88,89^.

The metagenomes represent freshwater systems across five trophic categories—eutrophic, meso-eutrophic, mesotrophic, oligo-mesotrophic, and oligotrophic—capturing broad ecological and geographical gradients of inland freshwater lakes and reservoirs. Samples included different filtration schemes depending on the original study design, with pore sizes and sampled fractions reported in Supplementary Data 1 and 2. Where both fractions were collected, 0.22-µm filters corresponded to the 0.22–5 µm fraction, whereas 5-µm filters corresponded to the >5 µm fraction. We therefore interpret the recovered mitoMAGs as the mitochondrial signal retained by the sampled filtration fractions, rather than as uniform coverage of all planktonic size classes. The assemblies were derived from either short-read or long-read sequencing technologies, as detailed alongside the computational tools and pipelines used for assembly in Supplementary Data 1. Briefly, reads were quality-filtered and trimmed using dataset-specific workflows, including Cutadapt v2.5, fastp v0.20.0, BBMap/BBNorm, Trimmomatic, PRINSEQ and sickle v1.33. Processed reads were assembled with short- or long-read metagenomic assemblers depending on the sequencing platform and original dataset. Short-read assemblies used metaSPAdes v3.8.0–3.15 with k-mer sizes of 21–127, or MEGAHIT v1.1.3–1.2.9 with k-mer sizes of 29–149. Long-read assemblies used Flye v2.8 with --plasmids and --meta, or Raven v1.5.0. Where applicable, assemblies were polished with Racon v1.4.13, Medaka v1.0.3 and/or Pilon v1.23 after read mapping with Minimap2 v2.17, Bowtie2 v2.3.5.1 or BWA-MEM v0.7.12. Unless otherwise stated in Supplementary Data 1, default parameters were used. All assemblies were uniformly processed to ensure consistency in downstream analyses.

### Contig length filtering

To enrich for contigs likely representing mitochondrial genomes, the assemblies were filtered by sequence length, retaining only contigs between 5 Kbp and 122 Kbp. These thresholds correspond to the 2.5th and 97.5th percentiles of mitochondrial genome lengths in the NCBI Reference Sequence Database^47^ (mitochondrial RefSeq r226), as determined from the size distribution of curated mitochondrial genomes. The lower bound was selected to remove very short contigs and extremely reduced mitochondrial fragments while retaining small mitochondrial genomes near the lower tail of RefSeq, including apicomplexan-like genomes of approximately 6 kbp^90^. Conversely, the upper bound (122 Kbp) was applied to exclude land plants mitochondrial genomes, whose sizes can exceed several hundred kilobases up to over 11 Mbp (e.g., *Silene conica* ~ 11.3), and are also unlikely to originate from aquatic samples. Together, these criteria were designed to maximize recovery of bona fide mitochondrial contigs while minimizing inclusion of non-mitochondrial or contaminant sequences, ensuring a robust dataset for downstream analyses. Importantly, the 5 minimum also ensures sufficient sequence complexity to support accurate k-mer–based processing steps. However, while the filter improves specificity, it may exclude very small mitochondrial genomes, minicircle-like molecules, or short architecture-specific fragments below 5 kbp.

### Machine learning-based identification of mitochondrial genomes/contigs

Mitochondrial candidates were identified using a custom-trained version of the Tiara v1.0.3^48^ software (default options). Tiara employs a deep neural network classifier trained on k-mer–based TF-IDF (Term Frequency-Inverse Document Frequency) features. All model hyperparameters were left at their default values to maintain comparability with the original implementation. The classifier was retrained using Tiara’s *train_tfidf.py* and *train_models.py* scripts (default settings) to improve discrimination between mitochondrial and non-mitochondrial sequences.

To enhance model generality and minimize overfitting, we tried to: (i) reduce redundancy and prevent dominance of overrepresented taxa and (ii) include sequences from a broad range of phylogenetic clades across all domains of life to ensure taxonomic diversity. These steps collectively ensured that the retrained classifier captured generalizable sequence signatures of mitochondrial origin rather than dataset-specific artifacts.

The bacterial model was trained on 80% of the total bacterial data from GTDB^91^ r95, and bacterial metagenome-assembled genomes (MAGs) from our previously published freshwater-centric pdCEL^92^ database (*n* = 3,519,318 sequences; approx. 30,238 species). The mitochondrial model was trained on 80% of the RefSeq r226 mitochondrial dataset (*n* = 14,139 sequences; n species = 14,139). Similarly, 80% of the archaeal data from GTDB r95 and the pdCEL database were used to train the archaeal model (*n* = 123,004 sequences; approx. 1672 species), 80% of the available plastid sequences available in NCBI (RefSeq r226) for the plastid model (*n* = 14,525 sequences), and finally, the default eukaryotic sequences provided by Tiara for the eukaryotic model (*n* = 1217 sequences). This multi-domain training strategy was designed to ensure robust boundary discrimination between mitochondrial and other genomic sequences while avoiding taxonomic bias or overfitting.

To evaluate model performance, all training datasets were pooled into a comprehensive benchmark set comprising 4,586,623 sequences. This dataset was then randomly subsampled using reformat.sh (BBMap—Bushnell B.—sourceforge.net/projects/bbmap/) script with the samplereadstarget = n option to generate paired subsets at 20% (n1 = 917,325 sequences; n2 = 917,325 sequences), 40% (n1 = 1,834,649 sequences; n2 = 1,834,649 sequences), 60% (n1 = 2,751,974; n2 = 2,751,974 sequences) and 80% levels (n1 = 3,669,298 sequences; n2 = 3,669,298 sequences). The newly trained Tiara (default options) software was then applied to each subset, and accuracy and precision were calculated (Supplementary Data 3). For cross-validation, the remaining 20% of each original training dataset—withheld during model construction—was independently tested using the retrained classifier. Accuracy and precision metrics were again recorded, confirming the high predictive performance and generalization capacity of the custom-trained Tiara model.

### Identity threshold for species-level clustering

As no universally accepted genomic species boundary exists for eukaryotes, we asked whether mitochondrial genomes exhibit a sequence-identity discontinuity—analogous to the bacterial ANI gap—between conspecific and congeneric taxa. We used RefSeq r226, which provides non-redundant, taxonomically vetted species (albeit skewed toward macro-eukaryotes), retaining only genera represented by ≥7 species (one mitogenome per species). This yielded 4349 genomes across 313 genera (median 10 per genus). To capture within-species variation, we added GenBank species with ≥7 conspecific mitogenomes, contributing 2205 genomes from 91 species (median 11 per species). Genomes were assigned to genus and species bins using NCBI Taxonomy identifiers, producing a hierarchical dataset with sufficient replication to characterize intra- versus interspecific ANI distributions. This framework revealed a natural inflection at 98.1% between the two distributions, from which we derived an empirically grounded ANI threshold for species-level clustering of mitogenomes. To validate the similarity threshold at which intra- and interspecific comparisons begin to overlap, we estimated the intersection of their empirical similarity distributions using a resampling-based kernel density approach. We considered 30 subsampling fractions, retaining between 99% and 70% of the available pairwise comparisons in 1% decrements. At each fraction, we randomly sampled (without replacement) the corresponding proportion of intraspecific (species-level) and interspecific (genus-level) pairwise similarities and estimated kernel density functions for each distribution over a restricted similarity window (90–100%). This window was chosen because overlap between intra- and interspecific comparisons is expected to occur only at high similarity values, and restricting the range avoids spurious intersections (artificial intersections) driven by low-density tails at lower similarities. For each subsample, we identified the similarity value at which the two density curves intersected by locating sign changes in the difference between the densities and linearly interpolating the crossing point. When multiple crossings were present, we retained the crossing associated with the highest mean density (i.e., the main region of overlap). Intersection values obtained across all subsampling fractions were summarized as mean ± s.d., which we report as the empirical species–genus similarity threshold (mean IS: 98.13 ± 0.08%). Applied to the full RefSeq r226 set, this threshold achieved ~92.4% accuracy and ~87.13% recall. Additional validations and results are provided in the Suppl. Information.

ANI values were computed following a method inspired by Goris et al. (2007)^93^. Briefly, the mitogenomes were fragmented into 1020 bp segments and subjected to reciprocal nucleotide BLAST (blastn) comparisons (minimum genome coverage of 80%). Alignment parameters included a maximum gap allowance of 150 bp and deactivation of low-complexity filtering (-F F) to ensure accurate detection of reciprocal best hits. These pairwise ANI distributions were then used to estimate the species-level identity cutoff applicable to the obtained mitogenomes clustering.

For comparison, a reference set of 11,797 small subunit (SSU) 18S rRNA sequences was obtained by using barrnap v0.9 (default settings) from the Ribosomal Operon Database (ROD)^94^. These sequences were assigned taxonomic labels based on the NCBI taxonomy and grouped into the same hierarchical categories—genus (*n* = 104, *n* = 1815 sequences; median = 10 species per genus) and species (*n* = 157, *n* = 4634 sequences, median = 12 sequences per species)—with a minimum of seven sequences per category, following the same criteria applied to mitochondrial genomes. The sequences were aligned using PRANK^95^ v.170427 with default parameters, and alignment identity scores were calculated from the obtained alignments. The median identity within each category served to define the 18S-based taxonomic thresholds.

To test whether mitochondrial genomic identity differs between macro- and microeukaryotes at the intra- versus interspecific levels, we controlled for unequal sampling with iterative rarefaction. In each of 1000 iterations, the larger group was randomly downsampled to the size of the smaller group, after which we computed two-sided Wilcoxon rank-sum p-values for (i) within-species comparisons and (ii) between-species comparisons. We then summarized the results by averaging p-values across iterations to assess overall significance.

### Clustering of the mitogenomes

We delineated species-level clusters with MMseqs2^55^ v16.747c6 (easy-cluster) using an empirically derived DNA identity threshold of 98.1% and query-side coverage ≥70%: mmseqs easy-cluster --min-seq-id 0.981 --cov-mode 1 -c 0.7 -s 7.5. We set --cov-mode 1 to enforce coverage on the query, which tolerates fragmented assemblies while guarding against spurious merges driven by short local overlaps. Clustering followed MMseqs2’s greedy set-cover procedure: the longest sequence in each cluster was chosen as the representative, and sequences were assigned to that cluster only if they met both the 0.981 identity and 0.70 coverage criteria. A high prefilter sensitivity (-s 7.5) ensured robust candidate detection prior to alignment. MMseqs2 performs strand-aware nucleotide alignments; we confirmed that reverse-complement orientation neither altered cluster membership nor changed representative selection.

### Cluster validation I: genetic code assignment and curation

To validate species-level mitochondrial clusters, we extracted the cluster representatives (*n* = 49,894) as the working dataset for building a high-quality mitochondrial reference atlas. Because codon reassignments are common in mitochondrial genomes, we first inferred the genetic code of each representative with Codetta^96^ v2.0.16 (confidence parameter *r* = 0.8). Predicted codon tables were then mapped to the 13 mitochondrial genetic codes registered by NCBI by scoring agreement between Codetta’s assignments and each reference code; the highest-scoring code was selected. When predictions were dominated by ambiguous calls (i.e., “?”), indicating an inconclusive inference, we conservatively assigned the standard genetic code (NCBI code 1). Using the assigned codes, we performed gene prediction with Prodigal^97^ v2.6.3 (custom code option) and translated the resulting open reading frames to derive proteomes. These proteomes were screened with HMMER^98^ (hmmscan v3.1b2) against TIGRFAMs^56^ v15.0, retaining hits with E-value ≤ 1 × 10^−3^ and both profile and HMM coverage ≥40%. Representatives yielding two or fewer annotated genes were removed due to insufficient signal for further reliable curation.

To further limit contamination and isolate outliers, we reclustered the remaining representatives at a relaxed nucleotide identity of 83.55% while retaining a ≥ 70% query-coverage requirement. Given the breadth of our atlas—spanning ~3400 freshwater datasets—we do not expect genuinely divergent mitochondrial lineages to appear only once. Accordingly, sequences that remained singletons at this coarse identity threshold were deemed likely contaminants and excluded.

To identify potential contaminants, we extracted mitochondrial rRNA genes (12S and 16S) from cluster representatives using barrnap v0.9 (mitochondrial mode; --kingdom mito --lencutoff 0.8 –reject 0.1 options) and classified them with blastn v2.9.0 against custom composite reference sets (E-value ≤ 1 × 10^−5^). The small-subunit and large-subunit sets comprised SILVA^49^ 138.1 SSURef NR99 (*n* = 452,421; ~65,743 species) and SILVA 138.1 LSURef NR99 (*n* = 95,286; ~25,819 species) together with RefSeq r226 mitochondrial rRNAs (12S, *n* = 17,108; 16S, *n* = 17,052) and chloroplast 12S (*n* = 28,130), yielding SSU *n* = 497,659 and LSU *n* = 112,338 total sequences. Representatives whose rRNAs matched bacteria or chloroplasts were further vetted by manual inspection of their predicted proteomes using the NCBI BLASTp web interface. Sequences for which both rRNA taxonomy and proteome matches supported bacterial or plastid origin were removed. We then re-clustered these curated representatives together with their underlying member sequences using MMseqs2 at 98.1% nucleotide identity and ≥70% query coverage, producing a set of 9192 representatives encompassing 38,081 clustered mitogenome sequences.

### Cluster validation II: multi-tool contig classification

To further assess and ensure whether the representative contigs corresponded to authentic mitochondrial sequences, a multi-tool validation pipeline integrating complementary classification approaches was employed. This framework combined viral detection, taxonomic classification, and deep-learning–based sequence composition analysis to conservatively exclude non-mitochondrial contigs and strengthen dataset reliability.

Potential viral contaminants were first screened using checkV^50^ v1.0.3 (default settings), VIBRANT^51^ v1.2.0 (options -f nucl) and Kaiju^52^ v1.10.1 (with viral database). CheckV estimates genome completeness and contamination based on the presence of viral hallmark genes and genome termini features, while VIBRANT identifies lytic and lysogenic viral genomes through machine-learning–based motif and protein-domain detection. Kaiju complements these tools by performing protein-level taxonomic classification via translated alignment against reference viral proteins. Contigs identified as viral by at least two of the three tools were conservatively excluded to prevent inclusion of integrated or co-extracted viral elements.

To further refine the dataset and remove non-viral but non-mitochondrial sequences, Kaiju v1.10.1 (RefSeq database, default parameters) and Metabuli^53^ v1.1.1 (default settings) were applied. Kaiju provides sensitive protein-level taxonomic assignment, enabling the detection of bacterial, archaeal, or plastid contamination, whereas Metabuli uses an alignment-free machine-learning classifier trained across major taxonomic domains, offering complementary detection of non-mitochondrial genomes. Finally, DeepMicroClass^54^ v1.0.3 (default settings) was used as an independent deep-learning classifier, capturing compositional and structural sequence signals. Genomes classified as non-mitochondrial by two or more tools across all methods were likewise removed from the dataset. After this multi-layer validation and conservative filtering, the dataset comprised 4331 validated mitochondrial cluster representatives, encompassing a total of 21,971 mitogenome members.

### Cluster validation III: annotation-based taxonomic classification

We assigned taxonomy to recovered mitogenomes and compared them against RefSeq r226 mitochondrial annotations. A custom MMseqs2 database (mmseqs createdb, default settings) was built by combining all RefSeq r226 mitochondrial proteomes (274,298 proteins from 18,084 species) with a large set of bacterial proteomes (75,274,782 proteins from ~47,499 species); each entry carried its full NCBI taxonomic lineage in the sequence header. This hybrid reference improves discrimination between mitochondrial and bacterial matches during classification.

Predicted mitochondrial proteins from each mitogenome were searched against this database using the MMseqs2 search workflow (alignment output enabled, -a). The obtained results were further converted into a BLAST-tab formatted file (using mmseqs convertalis) from which individual top hits (cut-offs: E-value 1 × 10^−5^, identity 20%, coverage 20%) were extracted, and their taxonomic labels were used to annotate the queried proteomes. Mitogenome taxonomy was then inferred with a consensus LCA procedure implemented in a custom Perl script (lca10.pl). For each predicted protein, all significant hits were retained; at each rank (species → genus → family → …), we assigned the most frequent label when it exceeded a majority (>50%). If no majority was achieved, we moved to the next higher rank. When no rank reached consensus because of ties or inconsistent classifications, the mitogenome was reported as “uncharacterized eukaryote.”

To prevent the inclusion of bacterial contaminants, mitogenomes in which a majority of predicted genes (>50%) were assigned bacterial taxonomy were excluded. After this stringent taxonomic validation and filtering, the dataset comprised a final set of 3662 representative mitoMAGs, encompassing 19,925 total cluster members, which formed the basis of the Freshwater Mitochondrial Atlas.

### Post hoc validation of mitochondrial fragmentation by binning

To assess whether distinct FMA clusters representatives could correspond to non-overlapping fragments of the same mitochondrial genome, we performed a post hoc binning validation using 429 metagenomes. Multi-sample contig coverage profiles were generated with Fairy v0.5.8^99^ using the sketch and coverage workflows with default settings. FMA species representatives were then binned with MetaBAT2 v2.2.17^100^ using default settings. Multi-fragment bins were considered candidate fragmentation events and were further evaluated using two consistency criteria: contigs within a bin were required to show congruent lowest common ancestor taxonomic assignments and non-redundant mitochondrial gene repertoires. Bins satisfying both criteria were interpreted as cases in which multiple recovered representatives likely corresponded to fragments of the same species-level mitochondrial unit. In total, 63 bins involving 114 FMA representatives fulfilled these criteria. These representatives were collapsed into 63 revised species-level units and used in downstream species-centric analyses.

### Cytochrome c oxidase subunit 1-based taxonomic classification

To further validate the taxonomic assignments of the recovered mitochondrial genomes (*n* = 19,925), cytochrome C oxidase subunit 1 (COI) genes were identified from the FMA mitochondrial genomes using TIGRFAM-based functional annotations. The extracted COI sequences (*n* = 7146) were then compared against a curated, publicly available reference COI sequence database (eKOI v1.0.0)^101^. Briefly, eKOI sequences (*n* = 15,947) were formatted for local similarity searches using makeblastdb v2.9.0+ (-dbtype nucl options). The recovered FMA COI gene sequences (*n* = 7146) were then queried against the eKOI reference by using blastn v2.9.0+ (-evalue 1e-5 -max_target_seqs 1 -max_hsps 1 -outfmt ‘6 qseqid sseqid pident length evalue bitscore’ options). Resulting hits were filtered to retain only alignments ≥400 bp, yielding 6991 high-confidence COI FMA matches. The taxonomic assignments derived from the COI alignment were subsequently compared with those inferred from LSU, SSU (see Suppl. Information), and LCA-based approaches to corroborate and refine the final taxonomy of each mitochondrial genome.

### Freshwater Mitochondrial Atlas: evolutionary history

To capture the evolutionary breadth of the environmentally recovered mitogenomes (*n* = 19,925), we analysed mitochondrial COI genes from FMA species-cluster representatives (*n* = 3662) together with sequences recovered from RefSeq r226. To focus the analysis on environmentally relevant eukaryotic diversity, we retained all sequences represented within the FMA (*n* = 1826) and supplemented them with RefSeq data that excluded the bulk of Metazoa and Viridiplantae. This curation yielded a refined, environment-focused RefSeq r226 subset comprised of 1411 COI sequences.

Sequences were aligned with MAFFT v7.453^102^ (default options), and further refined using ReAlign-P^103^ v1 (-m mafft option). The final polished alignment comprised 3237 sequences (FMA *n* = 1826; RefSeq r226 *n* = 1411) with an average identity of 60%. Maximum-likelihood phylogenies were inferred with IQ-TREE^104^ v3.0.1 under the LG + C10 + F + R10 model. Node support was assessed using 1000 ultrafast bootstrap replicates (-bb 1000) and 1000 SH-aLRT replicates (-alrt 1000). Trees were inferred as unrooted, with no outgroup designated.

### Freshwater Mitochondrial Atlas: diversity

To quantify taxonomic enrichment, the total number of mitogenomes assigned with taxonomy in the FMA dataset was compared to the corresponding species counts in RefSeq r226. Enrichment was expressed as the percentage increase in representation relative to RefSeq r226 and calculated as the relative increase in the number of mitogenomes recovered in the FMA dataset compared to those available in RefSeq r226. Specifically, for each taxonomic group, the difference between the FMA and RefSeq r226 counts was divided by the RefSeq r226 count and multiplied by 100, providing the percentage increase in representation.

To evaluate the phylogenetic contribution of recovered mitogenomes, we analysed the three microeukaryote lineages with the greatest species recovery—SAR, Cryptophyceae, and Haptista—combining FMA cluster representatives (SAR *n* = 2012; Cryptophyceae *n* = 266; Haptista *n* = 54) with RefSeq representatives (SAR *n* = 261; Cryptophyceae *n* = 11; Haptista *n* = 6).

Protein-coding genes were predicted using Prodigal^97^ v2.6.3, applying lineage-specific genetic codes (see “Methods” section; Cluster validation I). The resulting proteomes were functionally annotated using HMMER hmmscan v3.1b2 against the TIGRFAMs v15.0 database (E-value ≤ 1 × 10^−5^; -prcov 40; -hmcov 40). Marker genes conserved within each lineage were identified based on TIGRFAM accession numbers (SAR *n* = 40; Cryptophyceae *n* = 40; Haptista *n* = 19), and for each marker, phylogeny-aware multiple sequence alignments (MSAs) were constructed employing FAMSA^105^ v1.2 CPU (default settings). The obtained MSAs were then concatenated into a supermatrix (SAR aligned sites = 47,918; Cryptophyceae aligned sites = 20,691; Haptista aligned sites = 10,258) and used to construct a maximum-likelihood phylogeny with IQ-TREE v2.6.3 (-m LG + F + R4 -bb 1000 -alrt 1000). Node support was assessed using ultrafast bootstrapping with 1000 replicates and the SH-aLRT with 1000 replicates.

Phylogenetic diversity (PD) was computed as the sum of all branch distances in each subtree. The gain in PD introduced by the FMA was then calculated as follows: PDtotal – PDReference genomes = PDgain. All calculations were carried out within R v.4.2.2 with the ape^106^ v.5.3 package.

### Freshwater Mitochondrial Atlas: function

To evaluate the functional profile of the newly generated Freshwater Mitochondrial Atlas, the predicted proteomes of all recovered mitogenomes (*n* = 19,925 genomes; *n* = 265,367 proteins; median = 10 proteins/genome) were functionally annotated and compared to reference mitochondrial proteomes from RefSeq r226 (*n* = 17,771 genomes; *n* = 274,298 proteins; median = 16 proteins/genome). This comparative framework enabled an assessment of the FMA’s representativeness relative to established mitochondrial diversity.

For TIGRFAM, functional annotations were carried out using HMMER (*n* = 238,229 annotations for FMA; *n* = 180,596 for RefSeq r226) as previously described (see “Methods” section; *Cluster validation I*). All TIGR identifiers were extracted from RefSeq r226 and compiled into a non-redundant marker set (*n* = 213). This marker set served as a reference to query the FMA proteomes, allowing the identification of both conserved and potentially novel TIGR domains. Marker abundance was quantified by scanning each mitogenome for the presence of the complete marker set and summing their occurrences; the same approach was performed for the absences (i.e., FMA new annotations).

An analogous analysis was conducted for KO identifiers from the KEGG database^57^. Both proteomes (i.e., RefSeq r226 and FMA database) were annotated with BlastKOALA^107^ (*n* = 162,473 annotations for FMA; *n* = 120,652 annotations for RefSeq r226) and a non-redundant set of KEGG IDs (*n* = 200) was extracted from RefSeq r226 to serve as a reference. Marker abundances were calculated following the same procedure as for the TIGRFAM-based analysis.

Pfam^58^ domain annotations were obtained using pfam_scan.pl v1.6^17^ script (*n* = 210,264 annotations for FMA; *n* = 254,788 annotations for RefSeq r226) using Pfam release 38.0. A non-redundant Pfam marker set (*n* = 398) from RefSeq r226 was employed for comparison. As above, the presence, absence, and abundance of domains were quantified across both datasets.

### Freshwater Mitochondrial Atlas: phylogenomics

To illustrate the diversity recovered by the Freshwater Mitochondrial Atlas (FMA; *n* = 19,925), we constructed a phylogenomic tree using FMA species-cluster representatives (*n* = 3662), together with reference mitogenomes from RefSeq r226 (*n* = 17,771), publicly available SAR mitochondrial genomes retrieved from NCBI (*n* = 2196), and additional mitochondrial genomes reported by Wideman et al.^69^ (*n* = 7). To focus on the recovered microeukaryotic diversity, both RefSeq226 and the SAR dataset were dereplicated by retaining a single representative mitogenome per genus, yielding a reduced reference collection of 821 genomes.

To reduce redundancy and facilitate downstream phylogenetic analyses, FMA mitochondrial genomes were clustered with MMseqs2^55^ v16.747c6 (easy-cluster) using a minimum sequence identity threshold of 70% and query-side coverage ≥80% (--min-seq-id 0.7 --cov-mode 1 -c 0.5 -s 7.5). This procedure generated a non-redundant set comprising 2160 FMA representative mitogenomes.

The combination of the dereplicated FMA and reference datasets resulted in a master dataset of 2981 mitochondrial genomes used for downstream phylogenetic analyses.

A custom mitochondrial marker-gene database (24 genes) was constructed using MitoCOGs^108^. Briefly, the MitoCOGs protein dataset was downloaded from the NCBI FTP repository (https://ftp.ncbi.nih.gov/pub/koonin/MitoCOGs/). Sequences affiliated with 24 conserved mitochondrial orthologous groups were extracted and clustered with MMseqs2 v16.747c6 (easy-cluster --min-seq-id 0.85 -c 0.8 --cov-mode 0). Protein sequences within each cluster were independently aligned with MAFFT^102^ L-INS-i v7.471, and Hidden Markov Models (HMMs) were generated from the resulting alignments using HMMER v3.3 (hmmbuild). Marker-specific e-value thresholds were empirically determined by contrasting scores obtained for target and non-target matches. The marker set included NADH dehydrogenase subunits 1, 2, 4, 5, 6, 7, 8, 9 and 11; cytochrome c oxidase subunits 1, 2 and 3; cytochrome b; ATP synthase subunits 1, 3 and 6; cytochrome c maturation protein ccmFN; and mitochondrial ribosomal proteins L2, L6, L11, L16, S4, S12 and S14

All mitochondrial genomes (*n* = 2’981) were screened against the custom HMM collection using hmmsearch v3.3. Genomes containing fewer than seven recovered markers were excluded from subsequent analyses. Recovered marker genes were independently aligned with MAFFT v7.453 (--auto option) and subsequently refined with ReAlign-P^103^ using default parameters. The resulting alignments were then gently trimmed using BMGE^109^ v1.12 (-t AA -g 0.98 -h 0.98 -m BLOSUM30 options). To mitigate the influence of rapidly evolving positions, single-marker maximum-likelihood phylogenies were inferred with IQ-TREE^104^ v3.1.2 under the LG + F + R10 model and used to estimate site-specific evolutionary rates (-n 0 --rate). The fastest-evolving 10% of sites were subsequently removed with siteStripper.pl (-r rates -rf iqtree -f 0.90). Final alignments were then concatenated into a supermatrix comprising 1936 mitochondrial genomes with an average pairwise amino acid identity of 35%. A guide phylogeny was subsequently inferred with IQ-TREE v3.1.2 under the LG + F + R10 model and used as a fixed starting tree for a PMSF approximation analysis employing the LG + C60 + F + G model. To root the mitochondrial phylogeny, ten bacterial genomes spanning Alphaproteobacteria (*n* = 3), Betaproteobacteria (*n* = 3), and Gammaproteobacteria (*n* = 4) were included as outgroups.

### Diversity estimation across trophic statuses

To evaluate and compare mitochondrial diversity across trophic statuses, the complete set of 19,925 mitogenomes recovered in this study was analyzed. Each mitogenome was previously assigned a trophic classification based on its freshwater origin as follows: eutrophic+meso-eutrophic (*n* = 9469), oligo-mesotrophic+mesotrophic (*n* = 4414), or oligotrophic (*n* = 5420). Mitogenomes lacking this information (*n* = 622) were excluded from the analysis. Within each trophic group, genomes were clustered at the species level using the same ANI threshold (98.1%) and MMseqs2 workflow described previously (*see* *“Methods”: Clustering of the Mitogenome*), yielding 2637, 1773, and 1868 species-level representatives for the eutrophic/meso-eutrophic, oligo-mesotrophic/mesotrophic, and oligotrophic categories, respectively. To approximate species incidence, the number of genomes within each cluster (i.e., cluster size) was used as a surrogate measure. Cluster size was used as a recovery-based representation of each species-level mitochondrial unit within a trophic category. We use this metric as an incidence-like measure of repeated mitochondrial recovery, rather than as a direct estimate of organismal abundance, biomass or mitochondrial copy number. Incidence data were compiled into a trophic group–by–species matrix and reformatted into a list suitable for rarefaction analysis.

To account for unequal sampling effort among trophic categories, coverage-based rarefaction and extrapolation were applied using the iNEXT^59^ v2.0.22 R package. Rarefaction curves were generated for diversity orders *q* = 1 (Shannon diversity), and 2 (Simpson-Gini diversity) using iNEXT() function. A common sample coverage level (75.8%) was determined from the intersection of the rarefaction curves across groups. Shannon (*q* = ) and Simpson-Gini (*q* = ) diversity indices were then estimated at this shared coverage using the estimateD() function. Bootstrap support from 1000 replicates was used to calculate 95% confidence intervals, ensuring statistical robustness. To assess differences among trophic categories, pairwise contrasts were obtained from the bootstrap distributions of diversity and evenness estimates, reporting the mean difference, 95% bootstrap confidence intervals, and a two-sided bootstrap tail probability quantifying the strength of evidence for divergence between groups (see Supplementary Data 10). Because bootstrap replicates reflect uncertainty in a single assemblage rather than independent samples, these tail probabilities are interpreted as measures of support rather than formal hypothesis-test *P* values.

### Diversity estimation across time and space

To assess phylogenetic diversity gain and quantify the seasonal and vertical structuring of freshwater mitochondrial lineages, rarefied Faith’s phylogenetic diversity (PD) and the standardized effect size (SES-PD) were computed for mitochondrial genomes species recovered from four nutrient-distinct German lakes—Breiter Luzin, Fuchskuhle, Stechlin, and Tiefwaren—monthly sampled across four seasons—winter, spring, summer, and autumn—and from years 2012 to 2020. Briefly, mitochondrial genomes originating from these four water bodies’ metagenomic samples (n total metagenomes = 843; 108 Breiter Luzin, 381 Fuchskuhle, 233 Stechlin, 121 Tiefwaren) were grouped according to the season in which each sample was collected. Sampling months were assigned as follows: December–February to winter, March–May to spring, June–August to summer, and September–November to autumn. This classification resulted in 580 mitochondrial genomes for winter, 1589 for spring, 937 for summer, and 1087 for autumn.

Within each season, genomes were clustered at the species level using the MMseqs2 easy-cluster workflow (*see* *“Methods”: Species threshold; Clustering of the Mitogenome*), yielding 305 species clusters for winter, 685 for spring, 426 for summer, and 454 for autumn. Cluster size served as a proxy for species incidence in all subsequent analyses. Phylogenetic analyses were performed for each seasonal dataset. Protein-coding genes were predicted and annotated following the same procedure as described previously (see “Methods” section; Phylogenetic diversity *SAR*, *Cryptophyceae* and *Haptista*). A set of 64 conserved marker genes was identified for all mitogenomes based on TIGRFAM accession numbers. Multiple sequence alignments (MSAs) were generated with FAMSA v1.2 CPU (default settings) and subsequently trimmed using BMGE^109^ v1.12 (-t AA -g 0.7). The obtained MSAs were concatenated into a supermatrix (aligned sites = 12,703), and a maximum-likelihood phylogeny was inferred using IQ-TREE v3.0.1 (-m LG + C10 + F + R). Two alphaproteobacterial genomes (GCA_002291925 and GCA_002937625) were used as outgroup taxa to root the tree.

The same analytical workflow was applied to evaluate vertical diversity structure within lakes. In this case, mitochondrial genomes were divided into surface (*n* = 3341) and deep layer (*n* = 944) categories according to their sampling depth. Species clustering was performed independently for each layer using MMseqs2 easy-cluster, yielding 1168 species clusters for the surface layer and 374 for the deep layer. Subsequent phylogenetic reconstruction followed the same pipeline described above: protein-coding gene prediction and annotation, identification of 64 conserved TIGRFAM marker genes, multiple sequence alignment with FAMSA v1.2 CPU, trimming with BMGE v1.12 (-t AA -g 0.7), and concatenation into a supermatrix of 12,729 aligned amino acid positions. A maximum-likelihood tree was inferred in IQ-TREE v3.0.1 using the LG + C10 + F + R substitution model.

Mitochondrial genome abundance data were imported and aggregated by season to construct a Season × ID abundance matrix. To account for uneven sequencing depth among seasons, equal-sample-size rarefaction was applied to the minimum total abundance across seasons using the vegan::rrarefy function^110^ (1000 replicates). For each rarefied community, Faith’s PD was computed with picante::pd^111^. This procedure yielded a distribution of PD values per season, summarized as the mean, standard deviation, and 95% confidence quantiles. To evaluate deviations from random phylogenetic structure, the standardized effect size of PD (SES-PD) was calculated using picante::ses.pd with the *taxa.labels* null model, which randomizes tip labels while preserving species richness. Each rarefied community was randomized 1000 times, and the entire SES-PD estimation was repeated 1000 times to incorporate both sampling and null-model variability. The same procedure was performed on the vertical lake strata data. All analyses were conducted in R using the packages ape v5.3, vegan v2.6.4, picante v1.8.2, iNEXT v2.0.22, and tidyverse v2.0.0 libraries for data handling.

### Diversity and strata connectivity

To evaluate vertical structuring in mitochondrial community composition across seasons, we calculated pairwise Jaccard distances between the surface and deep layer samples using 299 metagenomes collected from Lake Stechlin, the deepest and largest among the four previously mentioned German lakes. A total of 1172 mitochondrial genomes likely to be detected across these samples were de novo clustered at the species level (see *Clustering of the Mitogenome*), yielding 398 representative mitochondrial genomes. Quality-filtered Illumina shotgun reads (~1.30 Tb total) were mapped against these representatives using BBMap, and the resulting alignments (sorted BAM files) were used to extract per-mitogenome coverage and breadth. To ensure robust presence–absence detection, only non-metazoan mitogenomes with ≥ 2 × coverage and ≥ 50% genome breadth were retained. This filtering resulted in a final dataset of 241 mitogenomes and 204 linked-metagenomes.

Between-layer comparisons were conducted on paired surface–deep layer samples within the same season, including only those sampled within 28 days of each other. If both compartments were sampled on the same day, that date was used; otherwise, the closest available date within the 28-day window was selected. Valid pair profiles were compared using Jaccard distances. A total of 77 seasonal surface–deep pairs met the criteria (spring = 31, summer = 12, autumn = 24, winter = 10). To estimate uncertainty around the seasonal means, we generated 1000 bootstrap resamples (sampling with replacement) of the Jaccard distances for each season and computed the mean for each resample. Final results are presented as seasonal mean Jaccard distances with 95% confidence intervals, derived from the 2.5th and 97.5th percentiles of the bootstrap distributions. All Jaccard distance calculations and statistical analyses were performed in R v4.2.2.

### Statistics

All statistical analyses were conducted in R v4.2.2 using RStudio v3.1.446 (Apricot Nasturtium). Data normality was assessed using the Shapiro–Wilk test and by visual inspection of residual distributions. Pairwise comparisons were performed using Student’s t-test for normally distributed data and the Wilcoxon rank-sum test for non-normal data. Differences among trophic states were tested using the Kruskal–Wallis test, followed by pairwise Wilcoxon rank-sum tests. Correlations for non-normally distributed variables were evaluated using Spearman’s rank correlation. All tests were implemented using functions from the stats package (v4.1.3), a core component of R.

The sampling map was generated in R using the ggplot2, readxl, raster, sf, and ggspatial packages. Sample metadata, including geographic coordinates and trophic status, were imported from an Excel spreadsheet using the readxl package. Sampling locations sharing identical coordinates were grouped, and the number of genomes at each location was summarized. A high-resolution Köppen–Geiger climate classification raster^112^ was imported into R using the raster package and converted into a data frame for visualization. Raster values were matched to the corresponding Köppen–Geiger climate classes and their associated color scheme. The climate raster was displayed as the map background, and sampling locations were overlaid as colored points according to trophic status using ggplot2. The legend, color palette, and map annotations (including the north arrow) were manually adjusted to improve figure readability.

No statistical method was used to predetermine sample size. No data were excluded from the analyses. The experiments were not randomized. The Investigators were not blinded to allocation during experiments and outcome assessment.

### Reporting summary

Further information on research design is available in the Nature Portfolio Reporting Summary linked to this article.

## Supplementary information

Supplementary Information

Description of Additional Supplementary Files

Supplementary Data 1–12

Reporting Summary

Transparent Peer Review file

## Source data

Source Data

## Data Availability

All mitogenomes generated in this study have been deposited in the European Nucleotide Archive (ENA) under project accession PRJEB95915 and in Zenodo (https://doi.org/10.5281/zenodo.20827346). Assembly and sample accession information for the 3400 input assemblies is provided in Supplementary Data 1. Identifiers, ENA analysis accessions and associated metadata for all 19,925 mitogenomes are provided in Supplementary Data 7. Custom scripts used in this study are available at https://github.com/MiELevog/A-Global-Mitogenomic-Atlas-Illuminates-Freshwater-Microeukaryote-Diversity. Source data are provided with this paper.
