# Supplementary material for: A global mitochondrial genomic atlas illuminates freshwater microeukaryote diversity
Source: Nat Commun. 2026 Sep 25;17:11139. doi: 10.1038/s41467-026-78160-1 (PMC13710963; doi:10.1038/s41467-026-78160-1)
Supplement: Supplementary file 1 [file 41467_2026_78160_MOESM1_ESM.pdf]

Supplementary Information

A Global Mitogenomic Atlas Illuminates Freshwater Microeukaryote Diversity

Lucas Serra Moncadas[1], Hans-Joachim Ruscheweyh[2,3], Jason Woodhouse[4], Michaela M. Salcher[5], Yusuke Okazaki[6], David Kamanda Ngugi[7], Michael Pester[8], Thomas Posch[1], Rohit Ghai[5], Hans-Peter Grossart[4,9,10], Shinichi Sunagawa[2,3], Jakob Pernthaler[1], Adrian-Stefan Andrei[1,*]

[1]Limnological Station, Department of Plant and Microbial Biology, University of Zurich, 8802 Kilchberg, Switzerland
[2]Institute of Microbiology, Department of Biology, ETH Zurich, 8093 Zurich, Switzerland
[3]Swiss Institute of Bioinformatics, 1015 Lausanne, Switzerland
[4]Department of Experimental Limnology, Leibniz Institute for Freshwater Ecology and Inland Fisheries, D-12587 Stechlin, Germany
[5]Department of Aquatic Microbial Ecology, Institute of Hydrobiology, Biology Centre of the Czech Academy of Sciences, 37005, Ceské Budejovice, Czech Republic
[6]Bioinformatics Center, Institute for Chemical Research, Kyoto University, 611-0011Uji, Japan
[7]GEOMAR Helmholtz Centre for Ocean Research Kiel, 24148 Kiel, Germany
[8]Chair of Microbial Physiology, Technical University of Munich, D-85354 Freising, Germany
[9]Institute of Biochemistry and Biology, Potsdam University, D-14469 Potsdam, Germany
[10]Berlin-Brandenburg Institute of Advanced Biodiversity Research, Free University, D-14195 Berlin, Germany

*Corresponding author. Email: stefan.andrei@limnol.uzh.ch

Species boundaries on a mitochondrial axis

Species are delimited by many kinds of evidence—morphology, ecology, reproductive isolation, genomes—and concordance among these axes varies by clade. To anchor a portable operational rule, we contrasted within- versus between-species mitogenome similarity in genera with ample sampling (≥7 species/genus; ≥7 mitogenomes/species) and took their intersection (98.1% genome identity) as a pragmatic, taxonomy-aware boundary for clustering. Across RefSeq r226 (17,771 named species), this threshold achieved ~92.4% precision and ~87.1% recall against current names, preserving most recognised species while avoiding any claim of universality. We envision the 98.1% boundary as a workable common currency, while recognising that species are biological entities rather than fixed DNA-identity cut-offs.

Where names and mitochondria disagree, the pattern is often informative rather than erroneous. High interspecific mtDNA identity can reflect (i) recent divergence and ongoing gene flow, (ii) incomplete lineage sorting, (iii) introgressive hybridization with mitochondrial capture, or (iv) lineage-specific features of mtDNA evolution (e.g., extremely slow mitochondrial substitution rates in corals[1]).

Systematic sources of high interspecific identity in RefSeq r226

<u>Hybrids and subspecies are included in the nonredundant database</u>

RefSeq r226 contains explicit hybrids (e.g., *Paralichthys olivaceus* × *Verasper variegatus*; *Tachysurus fulvidraco* × *T. vachelli*) and subspecies (e.g., *Ursus thibetanus* subspp.). Maternal inheritance means a hybrid's mitogenome usually resolves to the maternal species, pulling hybrids and maternal species into the same cluster—an expected outcome, not a failure of clustering. Reviews across animal systems document frequent mitochondrial capture with minimal nuclear introgression[2].

<u>Clades repeatedly forming multi-species clusters at 98.1%</u>

Clustering RefSeq r226 mitogenomes at 98.1% identity yields the densest multi-species clusters in fishes, with additional instances in corals, mammals, etc. In

freshwater cyprinids, for example, our clusters merge multiple species and named hybrids across *Megalobrama*, *Carassius*, *Cyprinus*, *Siniperca*, *Schizothorax*, and sturgeons (*Scaphirhynchus*, *Acipenser*), and in marine groups they merge species in *Thunnus*, *Sebastes*, *Takifugu*, and *Epinephelus*. Mechanistically, three well-known processes can explain why distinct binomials share high identity in mitochondria. (i) Strict maternal inheritance and recorded hybridization: in the genera above (*e.g.*, *Megalobrama terminalis* × *Culter alburnus*; *Carassius auratus* × *Cyprinus carpio*; *Takifugu rubripes* × *T. flavidus*; *Epinephelus moara* × *E. lanceolatus*; *Scaphirhynchus albus* × *S. platorynchus*; *Acipenser gueldenstaedtii* × *A. baerii*), the mitogenomes track the maternal species, collapsing species+hybrid names into one 98.1% cluster—exactly as expected under animal mtDNA inheritance[3–7]. (ii) Recent divergence with incomplete lineage sorting and reticulation during adaptive radiations, classically in East African cichlids: in our data, several Lake Malawi genera (*Trematocranus*, *Nimbochromis*, *Dimidiochromis*, *Cheilochromis*, *Protomelas*, *Placidochromis*) coalesce at 98.1%, consistent with genome-wide evidence of gene flow and shared ancestral variation in the radiation. Analogous dynamics occur in lacustrine whitefish (*Coregonus*), chars (*Salvelinus*), and in marine rockfishes (*Sebastes*)[8–10]. (iii) Third, intrinsically slow mitochondrial substitution in Anthozoa, combined with hybridization, keeps interspecific identities high in our coral clusters (*Acropora*, *Porites*, *Orbicella*, *Sinularia*, *Tubastraea*)[1,11–13]. Finally, mammal cases in our clusters (e.g., *Ursus thibetanus* subspecies; multiple *Canis lupus* forms including *C. l. familiaris*; *Lepus*) fit a broader picture in which introgression produces mito–nuclear discordance—well established for bears (*Ursus maritimus* ↔ *U. arctos*), wolves/dogs, and hares (*Lepus*)[14–16].

Accordingly, we treat 98.1% DNA identity as a pragmatic separator rather than a universal boundary: even without hybridization, reticulation or extreme rate effects, some named species main remain indistinguishable by mitogenomes alone.

The breadth of mitochondrial diversity through the SSU-LSU lenses

Rationale

We use mitochondrial 12S–16S rRNA markers[17,18] to complement the COI-based phylogeny results depicted in Fig. 3b, and to emphasize the diversity gain brought by FMA. This phylogenetic reconstruction is not intended to refine deep eukaryotic relationships, but rather to highlight gains and remaining gaps in current mitochondrial references.

Method

To illustrate the evolutionary breadth of environmentally recovered mitogenomes, we analysed mitochondrial 16S and 12S rRNA genes from FMA species-cluster representatives together with RefSeq r226 ones. The initial combined dataset comprised 16S (n = 19,202) and 12S (n = 19,052) sequences. We removed short sequences (<650 bp for 16S; <400 bp for 12S), retaining 11,588 and 17,984 sequences, respectively. To focus on environmentally relevant microeukaryotic diversity, we excluded from RefSeq r226 a subset of metazoan mitogenomes (n = 7,904) with extensive existing genomic representation and limited relevance to freshwater microeukaryotes (e.g., many fishes, birds, and mammals). After this filter, 2,777 (16S) and 8,032 (12S) sequences were retained. These were then aligned with FAMSA v1.2 CPU and trimmed with BMGE[19] v1.12 (-m DNA -g 0.5) to remove ambiguously aligned sites. The two loci were then concatenated into a supermatrix, allowing partial occupancy (missing loci coded as gaps), yielding 8,466 taxa in total (RefSeq r226, n = 6,924; FMA, n = 1,542). Maximum-likelihood trees were inferred with IQ-TREE[20] v3.0.1 using a GTR+F+I+G4 model (-m GTR+I+G4+F); node support was evaluated with ultrafast bootstrap (1,000 replicates; -bb 1000) and the Shimodaira-Hasegawa approximate likelihood ratio test (SH-aLRT;1,000 replicates; -alrt 1000). Trees were inferred unrooted (no outgroup designated).

Results

To illustrate the additional phylogenetic breadth contributed by the FMA, we assembled a maximum-likelihood tree from concatenated mitochondrial 12S + 16S rRNA sequences. The inclusion of FMA lineages extended representation across several deep mitochondrial clades–most prominently within sparsely sampled microeukaryote groups, such as SAR, and, more precisely, within stramenopiles and alveolates; thereby bridging notable gaps in the present reference databases.

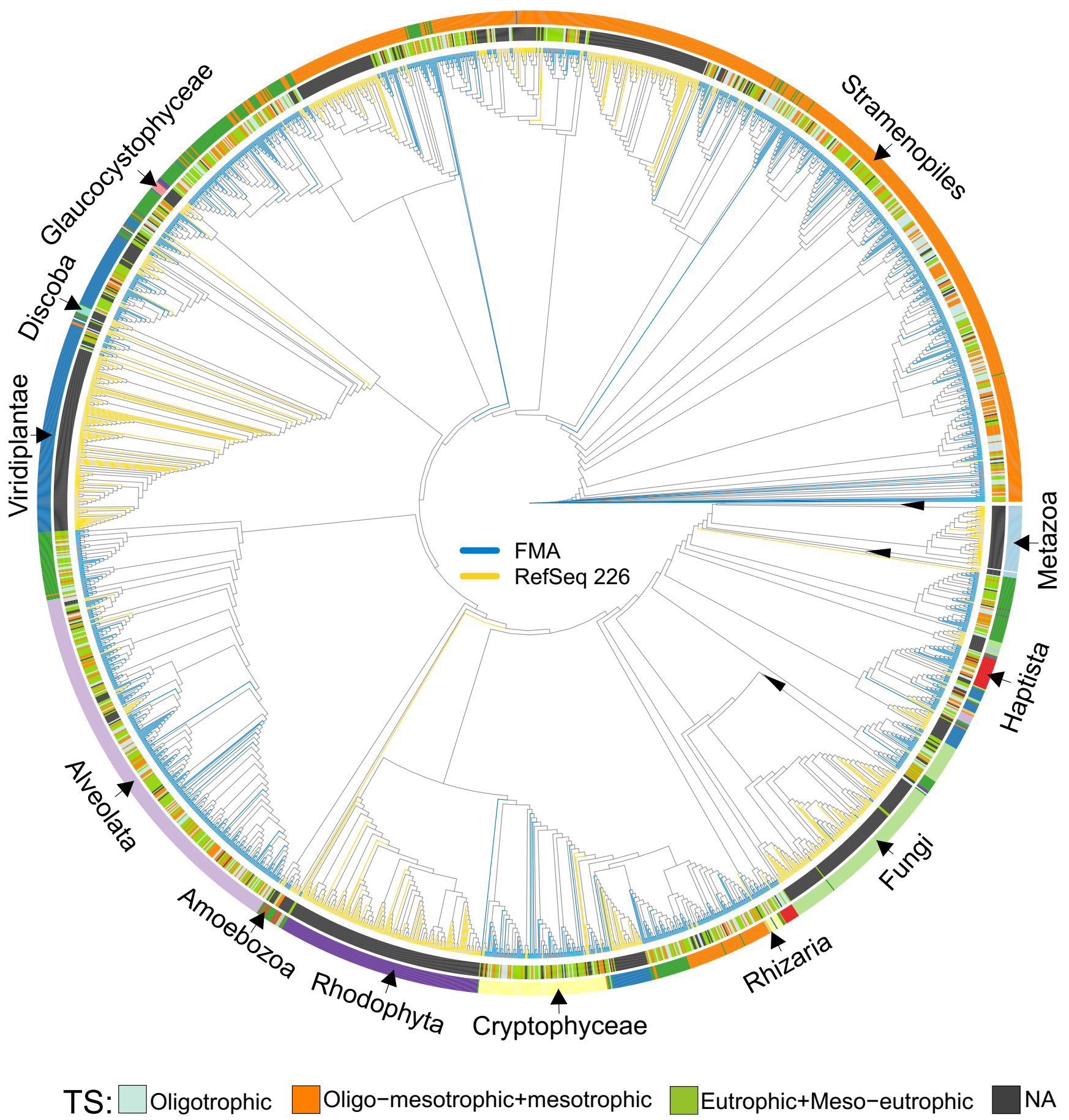

Suppl. Fig. S1 | Phylogenetic and compositional landscape of the LSU-SSU mitochondrial region. Maximum-likelihood phylogeny from concatenated mitochondrial 12S+16S rRNA genes (IQ-TREE, GTR+F+I+G4), comprising RefSeq r226 mitogenomes (yellow; n = 6,924) and 1,542 FMA mitogenomes (blue). Concentric rings (outer → inner) depict the taxonomy and the trophic status of the sampling site for the FMA records. Taxonomic ranks with low representation were grouped into a single category (grey), and uncharacterized mitochondrial genomes are shown in dark green. Black arrows indicate collapsed branches. Raw data is provided as a Source Data file.

Discussion

The 12S–16S phylogeny, complementary to the COI one, provided a synoptic view of how the FMA restructures mitochondrial sampling across the eukaryotic tree. Against the backdrop of a RefSeq r226 subset, the FMA tips the representation away from the familiar overabundance of animals and plants and into historically neglected branches, most visibly within the microbial eukaryotes. Stramenopiles, alveolates, rhizarians, and cryptophytes all gain extensive new coverage, with FMA lineages threading into regions of the tree that were previously sparsely populated in RefSeq r226. Rather than simply adding more tips to already well-sampled clades, the FMA introduces breadth at deeper nodes and across multiple supergroups, converting swaths of phylogenetic “white space” into populated structure. Yet the tree also reveals long, isolated branches populated by uncharacterized mitochondrial lineages (Suppl. Fig. S1, dark green, outer ring).

a

Most abundant annotations	
TIGR01770	NAD(P)H-quinone oxidoreductase subunit 2
TIGR01972	NADH-quinone oxidoreductase subunit M
K02256	Cytochrome c oxidase subunit 1
K02262	Cytochrome c oxidase subunit 3
PF00361.26	NADH-ubiquinone oxidoreductase chain 5
PF00146.26	NADH-ubiquinone oxidoreductase chain 1

b

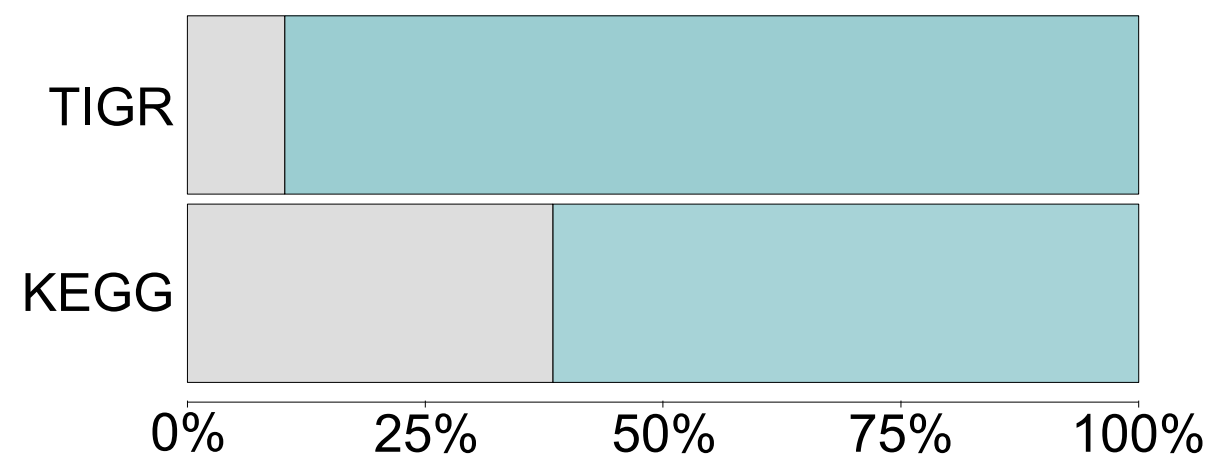

Suppl. Fig. S2 | Most frequent gene families and annotation coverage across FMA mitogenomes. (a) For each database (TIGRFAM, KEGG, Pfam), the two most frequent protein families detected among FMA mitochondrial coding sequences (CDSs) are listed with their identifiers. (b) Stacked bars show annotation coverage for the FMA mitogenome set: the blue segment is the proportion of CDSs with ≥1 assignment in the respective database, and the grey segment is unassigned CDSs. Percentages are calculated over all CDSs from the FMA mitogenomes (X-axis, 0–100%). Raw data is provided as a Source Data file

Sequence-discrete recruitment as an *in situ* check on the mitochondrial species boundary

Rationale

Genome-resolved studies of prokaryotes in natural environments consistently reveal the existence of sequence-discrete populations. In read recruitment to a reference genome, most reads align at very high identity to the local population (typically >95–100%); mapping density then drops sharply across ~90–95% identity, with only a sparse <90% tail from more distantly related genomes or spurious alignments[21]. This coverage–identity geometry—tight high-identity peak separated by a trough—is the *in situ* signature of ecologically cohesive, sequence-discrete populations (operational "species") and mirrors the bimodal species distribution of intergenomic ANI with a break near ~95% across large genome cohorts[22]. We asked whether environmental reads recruited to locally derived mitogenomes show the same pattern, and whether our 98.1% mitogenome identity threshold coincides with a similarity "gap" resembling the one observed for conspecific and non-conspecific matches in prokaryotes.

Method

We analysed 97 metagenomes from Lake Stechlin (Germany). Each library was subsampled to 30 million reads with reformat.sh (default settings), yielding 2.91×10^9 reads in total. Reads were recruited against a curated set of 38 Lake Stechlin mitogenomes (taxonomically diverse and abundant). Recruitment used blastn (BLAST+) with stringent filters: minimum alignment length ≥ 100 bp and E-value ≤ 1×10^{-5}. A reference was counted as present in a sample only if ≥ 3× coverage was contributed by 99–100%-identity reads. For each metagenome, we binned the percent identity of all retained hits into 1% bins (63–100%).

Results

Across the 97 datasets, identity distributions are strongly skewed, with a dominant mode at 99–100% and a pronounced coverage cliff just below ~98% (Suppl. Fig.

S2). Only a small minority of reads fall in the 97–95% interval and counts decline further at lower identities. Relative to the 98.1% line, the histogram separates into two regimes: very high-identity matches consistent with local conspecific lineages, and substantially lower identities attributable to related but distinct lineages or occasional cross-mappings.

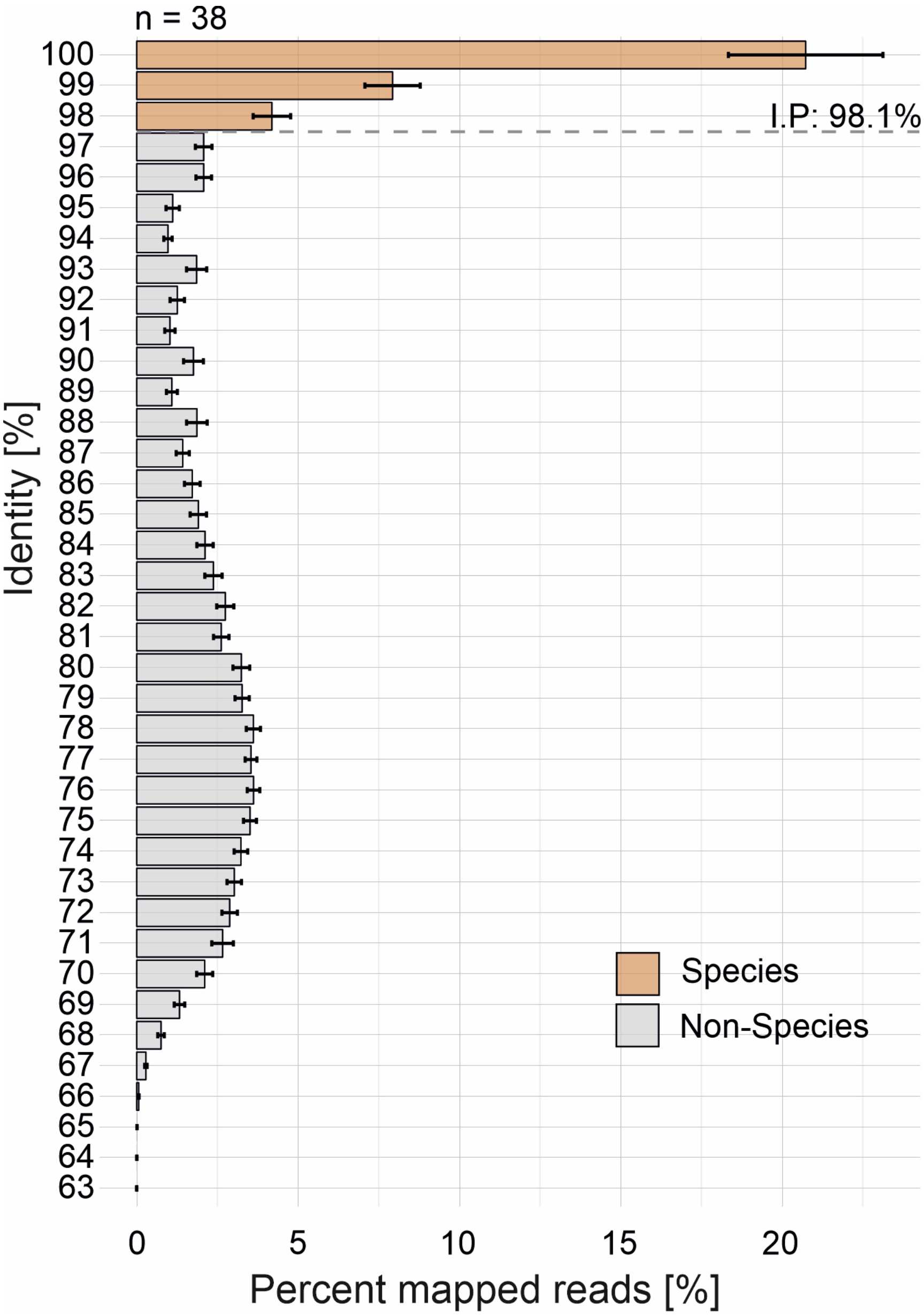

Suppl. Fig. S3 | Identity distribution of environmental reads recruited to lake mitogenomes. Bars show the mean proportion of recruited reads per 1% identity bin across 97 Lake Stechlin metagenomes; error bars indicate standard errors across samples; n = 38 denotes the number of reference mitogenomes queried. The dashed line marks the a priori 98.1% species threshold. Colours distinguish bins above ("Species") and below ("Non-Species") the threshold. Recruitment used BLASTn (alignment length ≥ 100 bp; E ≤ 1 × 10^{-5}). A reference was considered detected in a

sample if ≥ 3× coverage was provided by 99–100%-identity reads. Raw data is provided as a Source Data file

Discussion

Environmental read recruitment to local mitogenomes reproduces the sequence-discrete, two-mode structure described for genome recruitment in prokaryotes[21]. The coverage cliff aligns with the 98.1% mitogenome threshold derived independently from within- versus between-species comparisons, providing an *in situ* validation that the threshold partitions conspecific signal from non-conspecific background. Uniform subsampling mitigates depth effects, and the requirement for ≥ 3× coverage from 99–100% reads ensures that the high-identity mode reflects genuine community membership rather than spurious genomic alignments.

Perspectives on mitochondrial genome completeness

Rationale

Genome-resolved metagenomics opens access to the functional repertoires of uncultivated microbes, but robust inference depends on credible completeness and contamination assessments. For bacteria and archaea, single-copy core-gene panels provide standardised estimates[23]. Mitochondria lack a universal rubric because gene repertoires, genome architectures (circular, linear, multipartite), and even genetic codes vary widely across eukaryotic lineages[24]. Common practice—checking expected gene inventories, apparent circularisation, and length against references[25]—is informative but intrinsically reference-dependent, and can mislead where lineage coverage is sparse or biased; as for microeukaryotes. Given that uneven representation, we adopt a conservative lower-bound strategy for FMA: lineage-specific mitochondrial marker sets, genome size and gene numbers; that minimise over-calling of completeness while acknowledging that deep divergence or reductive evolution will likely depress scores.

Method

We approximated completeness using three complementary approaches. First, we used taxon-specific marker sets derived from RefSeq r226. Within each major lineage, a gene was designated a marker if present in ≥90% of curated mitogenomes (permissive to accommodate natural variability). Annotation followed our standard pipeline (see Methods: Cluster Validation I), and 12S/16S rRNA were included across all taxa. Marker-set sizes were: Aphelida (n = 13), Arthropoda (n = 16), Ascomycota (n = 18), Basidiomycota (n = 18), Ciliophora (n = 22), Cryptophyceae (n = 59), Discoba (n = 120), Fungi incertae sedis (n = 19), Rhizaria (n = 34), Stramenopiles (n = 55), Spiralia (n = 16) and Chlorophyta (n = 52). For each assembly, completeness was the fraction of lineage markers detected. Because homology detection degrades with divergence, we interpret these values explicitly as lower bounds. Second, we estimated completeness based on assembly size by computing, for each lineage, the median mitochondrial genome size among reference mitogenomes and expressing completeness as the ratio between the observed assembly size and this lineage-specific median. Third, we assessed completeness based on gene content by determining the median number of detected genes per lineage among references and calculating the fraction of this median gene count recovered in each assembly. Finally, for each assembly we averaged the three estimates—marker recovery, size-based completeness, and gene-content completeness—to obtain a combined completeness measure that provides a conservative yet more robust approximation than any single metric alone.

Results

The framework delivered lineage-aware, conservative completeness estimates. Well-sampled clades (e.g., fungi, arthropods, streptophytes) produced compact marker sets with consistent recovery, supporting confident calls. In contrast, lineages with sparse or distant references—notably Haptista (n = 6 references), Cryptophyceae (n = 11 references), and parts of SAR (n = 316 references)—showed broader dispersion and lower medians. In these groups, reduced scores likely reflect a mixture of lower recovery, true biological idiosyncrasy (e.g., rRNA fragmentation, gene transfers to the nucleus, reductive evolution) and database

divergence that attenuates marker detectability. Importantly, the additional size-based and gene-count completeness metrics help contextualize these patterns: assemblies scoring low in markers but approaching expected size or gene content likely represent divergent but near-complete mitogenomes rather than technical assembly failures. Many mitogenomes recovered in FMA come from exactly these undersampled clades, thereby expanding reference space and setting the stage for more faithful marker sets in the future.

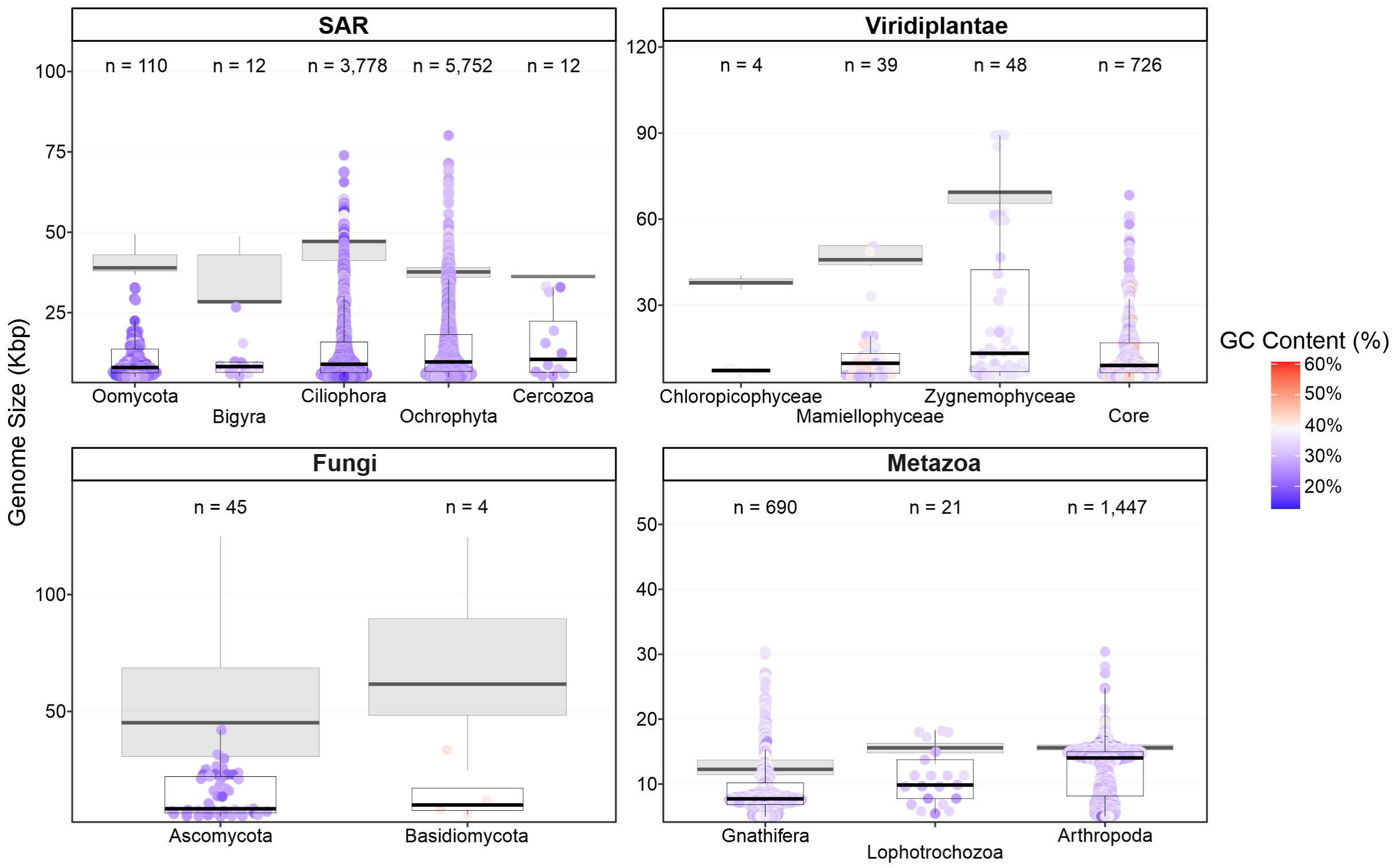

Suppl. Fig. S4 | Mitochondrial genome size distributions across taxonomic groups with GC content variation. Figure showcases recovered mitochondrial genome sizes (Kbp) (transparent boxplots) across major eukaryotic taxonomic groups (i.e., SAR, Viridiplantae, Fungi and Metazoa), against reference mitochondrial genomes from RefSeq r226 (grey boxplots in the background). Each point represents an individual mitochondrial genome (n = 19,925) and is colored according to its GC content, with a color gradient from blue (low GC) to red (high GC), as indicated on the right label. The central line across the boxplots identifies the median, marking the dataset's midpoint. The box itself demarcates the interquartile range, extending from the first quartile to the third quartile, encapsulating the central 50% of the data. The whiskers project from the box to the furthest data points not categorized as outliers and show the spread of the main body of the dataset. Raw data is provided as a Source Data file.

Discussion

A single universal completeness metric for mitochondrial genomes is not yet realistic. Our lineage-specific marker approach (combined with genome size and gene numbers) is conservative by design and yields estimates that should be read as approximations—especially where references are sparse or evolutionarily distant. In practice, completeness assessment should triangulate marker recovery with orthogonal evidence (phylogenetic similarity, circularization signals, synteny to close relatives) and be interpreted in light of lineage biology (e.g., hydrogenosomes/mitosomes, RNA editing, genome fragmentation). As new mitogenomes from microeukaryotes enter public databases—including many contributed here—marker sets will tighten, reducing divergence penalties and improving the accuracy of completeness estimates.

Suppl. Fig. S5 | Taxonomic representation of the mitochondrial species-threshold calibration datasets. Bars show the number of unique taxonomic groups represented by macroorganisms and microorganisms across ranks in the interspecies and intraspecies reference datasets used to infer the 98.1% mitochondrial genome identity threshold. Values above bars indicate the number of unique taxa per rank.

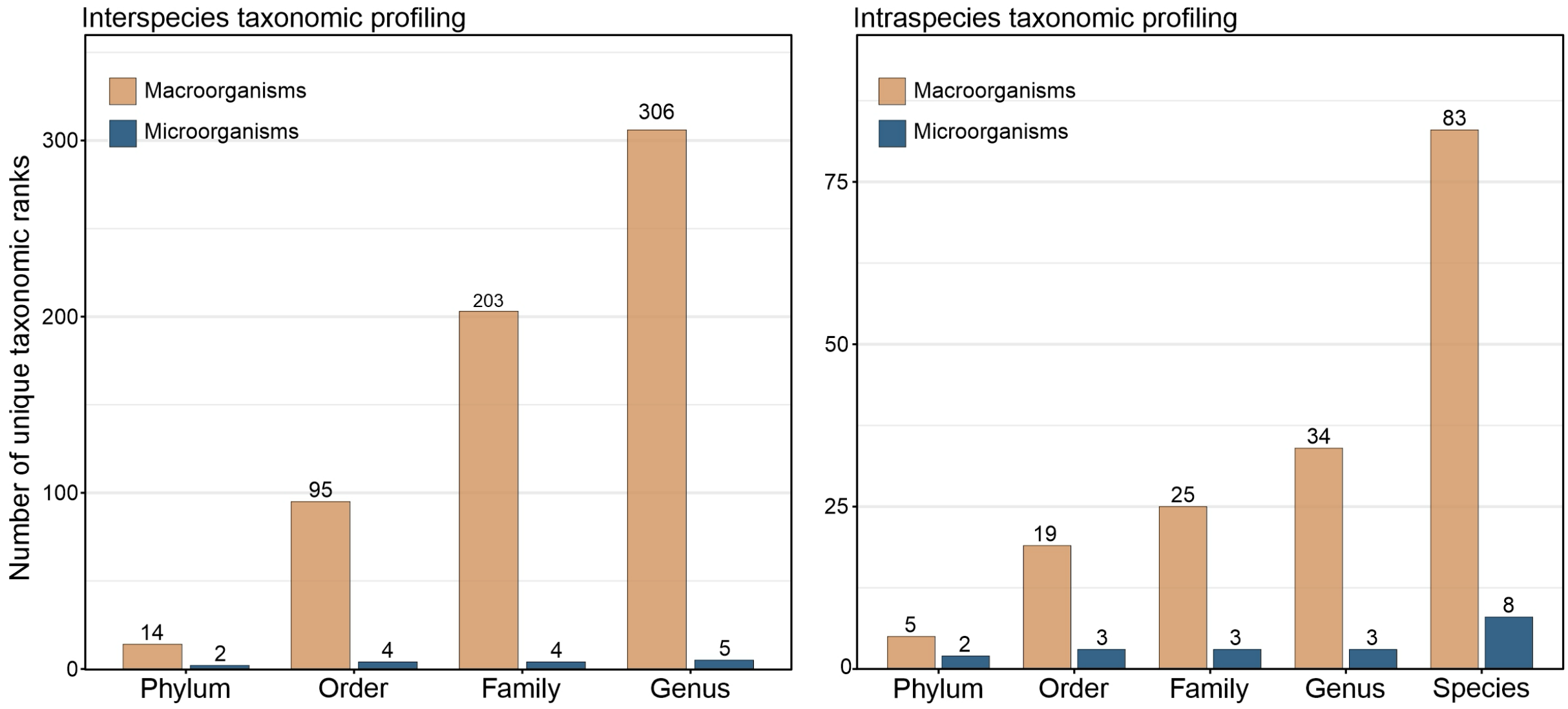

Mock-community benchmarking of sequencing and assembly effects

Rationale

To evaluate how sequencing technology, assembly strategy, and genome fragmentation could influence recovery of microeukaryotic organellar genomes in our metagenomic dataset, we performed a controlled mock-community benchmarking experiment using the ZymoBIOMICS Microbial Community Standard (D6300; Zymo Research). This mock community was selected because it is strongly dominated by bacterial DNA, while also containing a low-abundance microeukaryotic component, thereby approximating the expected taxonomic structure of our environmental samples. In particular, the community includes *Saccharomyces cerevisiae* strain Y-567, whose mitochondrial genome is approximately 80 kbp in length.

Method

Both short-read and long-read sequencing approaches were applied to the mock community. Total sequencing output was broadly comparable between platforms, with 15.97 Gb generated from Illumina sequencing and 19.69 Gb from Oxford Nanopore sequencing. For long-read sequencing, approximately 1 μg of high-molecular-weight DNA was used for library preparation with the Oxford Nanopore Ligation Sequencing Kit V14 (SQK-LSK114; Oxford Nanopore Technologies). Libraries were sequenced on R10.4.1 flow cells (FLO-MIN114) using MinION Mk1B and GridION platforms. Basecalling was performed with Dorado v0.4.2 in super-accurate mode using the dna_r10.4.1_e8.2_400bps_sup@v4.2.0 model, with adapter trimming applied at both read ends. For short-read sequencing, approximately 200 ng of DNA was sequenced on an Illumina NovaSeq 6000 platform using 2 × 150 bp paired-end reads. Nanopore reads were quality filtered with Chopper v0.7.0 using -q 10 -l 500 and screened for residual adapters with Porechop_ABI v0.5.1. Illumina reads were quality and adapter trimmed with bbduk.sh from BBMap v39.01 using qtrim=rl trimq=18 k=21

ref=adapters_and_contaminants.fa, and overlapping paired-end reads were merged with bbmerge.sh using default settings.

To assess the effect of assembly strategy, reads from each technology were assembled independently using multiple metagenomic assemblers. Illumina reads were assembled with MEGAHIT (v1.2.9) and metaSPAdes (v3.15.5), whereas Nanopore reads were assembled with metaFlye (v2.9.3-b179735) using --meta --nano-hq, and with Canu (v2.2). We then identified contigs corresponding to the *S. cerevisiae* mitochondrial genome, including fragmented mitochondrial assemblies where complete circular recovery was not achieved. Recovered mitochondrial genome fragments were compared across assemblies using pairwise average nucleotide identity.

Results

Pairwise ANI values among the recovered *S. cerevisiae* mitochondrial genome fragments were consistently high across all assembly strategies, ranging from 99.94% to 100%. The median pairwise genomic identity was 99.97%, indicating that the recovered mitochondrial sequences were highly concordant despite differences in sequencing platform, read length distribution, and assembler algorithm. This suggests that, although assembly fragmentation may vary among methods, the underlying sequence reconstruction of the *S. cerevisiae* mitochondrial genome was robust and reproducible across technologies. These results are consistent with previous metagenomic benchmarking studies showing that assembly strategy can affect contiguity while preserving high sequence-level accuracy in well-supported genomic regions[26].

Suppl. Table S1 | Genome-property comparison between FMA mitoMAG representatives and RefSeq r226 mitochondrial references across major taxonomic groups. For each taxonomic group, the table reports median sequence length (kbp), median GC content (%), median predicted gene number, and the number of records included for both FMA species-level representative mitoMAGs and corresponding RefSeq r226 mitochondrial genomes*.

Taxonomy	median_size_kbp_FMA	median_size_kbp_RefSeq226	median_gc_FMA	median_gc_RefSeq226	median_genes_FMA	median_genes_RefSeq226	n_genomes_FMA	n_genomes_RefSeq226
Arthropoda	15.089	15.593	33.45	23.3	11	12	111	5949
Ascomycota	22.994	45.1845	27.74	26.9	16	27	17	458
Bigyra	8.593	28.382	29.68	21.9	6	31	7	7
Ciliophora	16.285	47.143	24.69	22.05	15	41	571	20
core chlorophytes	15.0395	45.175	33.845	37.7	12	35	186	49
Gnathifera	12.033	12.268	33.98	32.8	8	11	109	10
Lophotrochozoa	11.288	15.579	30.98	33.2	7	13	7	1269
Mamiellophyceae	12.044	45.831	36.17	39.15	13	48.5	18	4
Ochrophyta	15.765	37.656	27.81	33.6	17	38	1349	163
Oomycota	10.009	38.94	23.16	22	11	41	65	70
Prymnesiophyceae	18.8345	29.013	31.865	29.4	21.5	27	40	5

*RefSeq r226 records are used as a group-level reference context for interpreting contiguity and gene recovery in the FMA. Because FMA records are metagenome-derived mitochondrial reconstructions and may be partial, differences in length or gene number should be interpreted as indicators of variable recovery, fragmentation, and taxon-specific mitochondrial genome architecture rather than as universal completeness estimates.

Read-level mitochondrial microdiversity analysis

To evaluate whether within-sample mitochondrial variation could influence mitoMAG interpretation, we performed a read-level microdiversity analysis on Lake Stechlin, Germany, one of the most deeply sampled systems in the dataset. This analysis was designed to quantify mitochondrial sequence variation directly from raw metagenomic reads, before consensus assembly. Because bulk metagenomic data cannot distinguish true intracellular heteroplasmy from within-population mitochondrial polymorphism or the co-occurrence of closely related mitochondrial haplotypes, we interpret this analysis as an upper-bound estimate of heteroplasmy-like mitochondrial microdiversity around recovered mitoMAGs.

Lake Stechlin contained 1,172 detected mitoMAGs across 299 Illumina shotgun metagenomes. To reduce redundancy and computational complexity, these mitoMAGs were clustered using the same species-level clustering procedure applied in the main analysis, yielding 398 representative mitoMAGs. Quality-filtered Illumina reads from the 299 metagenomes were mapped against these representative mitoMAGs using BBMap. The resulting sorted BAM files were analysed with inStrain[27] v1.8.0 using default settings to quantify read-level nucleotide variation across the recovered mitochondrial representatives.

Profiles were retained only when the mitoMAG had at least 2× mean coverage and at least 50% breadth of coverage. The 50% breadth threshold follows inStrain recommendations for robust genome detection and reduces false-positive profiles caused by sparse or local read recruitment[27]. For each retained mitoMAG profile, we extracted the number of divergent sites normalized per kilobase. These values were then compared with the divergence implied by the FMA species-level clustering boundary. Because the species-level cutoff is 98.1% sequence identity, the corresponding divergence boundary is 1.9%, equivalent to 19 divergent sites per kilobase.

The density of divergent sites was generally low. Across retained profiles, the mean was 2.82 ± 4.43 divergent sites per kilobase, with a median of 1.07 divergent sites per kilobase and an interquartile range of 0.22–3.56 divergent sites per kilobase.

Expressed as approximate sequence divergence, this corresponds to a mean of 0.282 ± 0.443% and a median of 0.107%. Thus, most read-level mitochondrial variation was well below the 1.9% divergence corresponding to the 98.1% species-level clustering threshold used in the FMA.

Consistent with this, 2,656 of 2,690 retained mitoMAG profiles, corresponding to 98.74%, remained below 19 divergent sites per kilobase. Only 34 profiles, or 1.26%, exceeded this boundary. These high-divergence cases are unlikely to represent ordinary low-level heteroplasmy alone. More plausibly, they reflect the presence of closely related mitochondrial lineages within the same sample. We therefore interpret them as rare cases of elevated mitochondrial microdiversity rather than as evidence that heteroplasmy broadly affects species-level clustering.

Overall, this read-level analysis indicates that mitochondrial microdiversity in Lake Stechlin is usually far below the sequence divergence required to cross the FMA species-level boundary. Heteroplasmy-like variation is therefore unlikely to be a major driver of the species-level richness or phylogenetic diversity patterns reported in the main manuscript.

References

1. Shearer, T. L., van Oppen, M. J. H., Romano, S. L. & Wörheide, G. Slow mitochondrial DNA sequence evolution in the Anthozoa (Cnidaria). *Molecular ecology* **11,** 2475–2487; 10.1046/j.1365-294x.2002.01652.x (2002).

2. Toews, D. P. L. & Brelsford, A. The biogeography of mitochondrial and nuclear discordance in animals. *Molecular ecology* **21,** 3907–3930; 10.1111/j.1365-294X.2012.05664.x (2012).

3. Hutchison, C. A., Newbold, J. E., Potter, S. S. & Edgell, M. H. Maternal inheritance of mammalian mitochondrial DNA. *Nature* **251,** 536–538; 10.1038/251536a0 (1974).

4. Park, J. Y., Yoon, J. H., Noh, C. H., Kang, M. J. & Bang, I.-C. Complete mitochondrial genome of the hybrid grouper Epinephelus lanceolatus (♀) × E. moara (♂). *Mitochondrial DNA. Part B, Resources* **3,** 1079–1080; 10.1080/23802359.2018.1507633 (2018).

5. Gao, F. *et al.* Characterization of the complete mitochondrial genome of the hybrid Epinephelus moara♀ × Epinephelus lanceolatus♂, and phylogenetic analysis in subfamily epinephelinae. *J. Ocean Univ. China* **16,** 555–563; 10.1007/s11802-017-3202-2 (2017).

6. Guo, W. *et al.* The complete mitochondrial genome of the hybrid of Siniperca chuatsi (♀) × Siniperca scherzeri (♂). *Mitochondrial DNA. Part A, DNA mapping, sequencing, and analysis* **27,** 1094–1095; 10.3109/19401736.2014.930838 (2016).

7. Jiang, H.-B., Bao, J. & Han, Y. Mitochondrial DNA sequence of the hybrid of Takifugu flavidus (♀) × Takifugu rubripes (♂). *Mitochondrial DNA. Part A, DNA mapping, sequencing, and analysis* **27,** 2117–2118; 10.3109/19401736.2014.982583 (2016).

8. Malinsky, M. *et al.* Whole-genome sequences of Malawi cichlids reveal multiple radiations interconnected by gene flow. *Nature ecology & evolution* **2,** 1940–1955; 10.1038/s41559-018-0717-x (2018).

9. Svardal, H. *et al.* Ancestral Hybridization Facilitated Species Diversification in the Lake Malawi Cichlid Fish Adaptive Radiation. *Molecular biology and evolution* **37,** 1100–1113; 10.1093/molbev/msz294 (2020).

10. Vonlanthen, P. *et al.* Eutrophication causes speciation reversal in whitefish adaptive radiations. *Nature* **482,** 357–362; 10.1038/nature10824 (2012).

11. Vollmer, S. V. & Palumbi, S. R. Hybridization and the evolution of reef coral diversity. *Science (New York, N.Y.)* **296,** 2023–2025; 10.1126/science.1069524 (2002).

12. Chan, W. Y., Peplow, L. M., Menéndez, P., Hoffmann, A. A. & van Oppen, M. J. H. Interspecific Hybridization May Provide Novel Opportunities for Coral Reef Restoration. *Front. Mar. Sci.* **5,** 353063; 10.3389/fmars.2018.00160 (2018).

13. Hobbs, J.-P. A. *et al.* Hybridisation and the evolution of coral reef biodiversity. *Coral Reefs* **41,** 535–549; 10.1007/s00338-021-02193-9 (2022).

14. Cahill, J. A. *et al.* Genomic Evidence of Widespread Admixture from Polar Bears into Brown Bears during the Last Ice Age. *Molecular biology and evolution* **35,** 1120–1129; 10.1093/molbev/msy018 (2018).

15. Fan, Z. *et al.* Worldwide patterns of genomic variation and admixture in gray wolves. *Genome research* **26,** 163–173; 10.1101/gr.197517.115 (2016).

16. Melo-Ferreira, J. *et al.* Recurrent introgression of mitochondrial DNA among hares (Lepus spp.) revealed by species-tree inference and coalescent simulations. *Systematic biology* **61,** 367–381; 10.1093/sysbio/syr114 (2012).

17. Chan, A. H. E., Chaisiri, K., Morand, S., Saralamba, N. & Thaenkham, U. Evaluation and utility of mitochondrial ribosomal genes for molecular systematics of parasitic nematodes. *Parasites & vectors* **13,** 364; 10.1186/s13071-020-04242-8 (2020).

18. Chan, A. H. E., Saralamba, N., Saralamba, S., Ruangsittichai, J. & Thaenkham, U. The potential use of mitochondrial ribosomal genes (12S and 16S) in DNA barcoding and phylogenetic analysis of trematodes. *BMC genomics* **23,** 104; 10.1186/s12864-022-08302-4 (2022).

19. Criscuolo, A. & Gribaldo, S. BMGE (Block Mapping and Gathering with Entropy): a new software for selection of phylogenetic informative regions from multiple sequence alignments. *BMC evolutionary biology* **10,** 210; 10.1186/1471-2148-10-210 (2010).

20. Nguyen, L.-T., Schmidt, H. A., Haeseler, A. von & Minh, B. Q. IQ-TREE: a fast and effective stochastic algorithm for estimating maximum-likelihood phylogenies. *Molecular biology and evolution* **32,** 268–274; 10.1093/molbev/msu300 (2015).

21. Bendall, M. L. *et al.* Genome-wide selective sweeps and gene-specific sweeps in natural bacterial populations. *The ISME journal* **10,** 1589–1601; 10.1038/ismej.2015.241 (2016).

22. Jain, C., Rodriguez-R, L. M., Phillippy, A. M., Konstantinidis, K. T. & Aluru, S. High throughput ANI analysis of 90K prokaryotic genomes reveals clear species boundaries. *Nature communications* **9,** 5114; 10.1038/s41467-018-07641-9 (2018).

23. Parks, D. H., Imelfort, M., Skennerton, C. T., Hugenholtz, P. & Tyson, G. W. CheckM: assessing the quality of microbial genomes recovered from isolates, single cells, and metagenomes. *Genome research* **25,** 1043–1055; 10.1101/gr.186072.114 (2015).

24. Gray, M. W., Lang, B. F. & Burger, G. Mitochondria of protists. *Annual review of genetics* **38,** 477–524; 10.1146/annurev.genet.37.110801.142526 (2004).

25. Wideman, J. G. *et al.* Unexpected mitochondrial genome diversity revealed by targeted single-cell genomics of heterotrophic flagellated protists. *Nature microbiology* **5,** 154–165; 10.1038/s41564-019-0605-4 (2020).

26. Meyer, F. *et al.* Critical Assessment of Metagenome Interpretation: the second round of challenges. *Nature methods* **19,** 429–440; 10.1038/s41592-022-01431-4 (2022).

27. Olm, M. R. *et al.* inStrain profiles population microdiversity from metagenomic data and sensitively detects shared microbial strains. *Nature biotechnology* **39,** 727–736; 10.1038/s41587-020-00797-0 (2021).
