# Supplementary material for: A global mitochondrial genomic atlas illuminates freshwater microeukaryote diversity
Source: Nat Commun. 2026 Sep 25;17:11139. doi: 10.1038/s41467-026-78160-1 (PMC13710963; doi:10.1038/s41467-026-78160-1)
Supplement: Supplementary file 2 [file 41467_2026_78160_MOESM2_ESM.pdf]

Description of Additional Supplementary Files

File Name: Supplementary Data 1

Description: Metadata for the 3,400 freshwater metagenomic assemblies analysed, including sample and run identifiers, assembly accession, location, source study, sequencing technology, assembly, polishing and mapping tools, and quality-control workflow.

File Name: Supplementary Data 2

Description: Geographic and trophic-status assignments for the 362 freshwater lakes and reservoirs included in the study, organized by country.

File Name: Supplementary Data 3

Description: Sensitivity and accuracy of the retrained Tiara classifier across sequence categories, subsampling thresholds and replicates.

File Name: Supplementary Data 4

Description: RefSeq release 226 mitochondrial genomes used to validate the species-level clustering threshold, with cluster representatives, cluster members and full taxonomic annotations.

File Name: Supplementary Data 5

Description: NCBI mitochondrial genetic-code translation tables used for gene prediction, provided in FASTA-style format.

File Name: Supplementary Data 6

Description: Metadata for the 3,662 FMA species-level representatives, including cluster size, genome size, GC content, gene count, LCA taxonomy, geographic origin, trophic classification and filter size.

File Name: Supplementary Data 7

Description: Identifiers, ENA analysis accessions, taxonomic annotations and associated metadata for all 19,925 FMA mitochondrial genomes and fragments, including species-representative assignments, rRNA and COI matches, genome properties and circularity.

File Name: Supplementary Data 8

Description: Taxonomic searches and metadata for 743 unassigned FMA representatives, including 12S, 16S, eKOI and NCBI COI matches, location, trophic status, filter size and genome properties.

File Name: Supplementary Data 9

Description: Taxonomic identities of 7,904 metazoan mitochondrial genomes excluded from the 12S-16S phylogenetic analysis.

File Name: Supplementary Data 10

Description: Statistical results supporting main Figures 2 and 5, including omnibus and pairwise tests, bootstrap comparisons, phylogenetic-diversity summaries, standardized effect sizes, and depth and seasonal contrasts.

File Name: Supplementary Data 11

Description: Fragment membership and genome properties for mitochondrial fragments consolidated into composite mitogenomes, including component identifiers, location, size, GC content and LCA taxonomy.

File Name: Supplementary Data 12

Description: Phylogenetic-diversity values calculated using mitochondrial fragments versus consolidated mitochondrial genomes for SAR and Cryptophyceae.
