# Supplementary material for: A global mitochondrial genomic atlas illuminates freshwater microeukaryote diversity
Source: Nat Commun. 2026 Sep 25;17:11139. doi: 10.1038/s41467-026-78160-1 (PMC13710963; doi:10.1038/s41467-026-78160-1)
Supplement: Supplementary file 3 [file 41467_2026_78160_MOESM4_ESM.pdf]

nature portfolio

Corresponding author(s): Adrian-Stefan Andrei

Last updated by author(s): Aug 4, 2026

Reporting Summary

Nature Portfolio wishes to improve the reproducibility of the work that we publish. This form provides structure for consistency and transparency in reporting. For further information on Nature Portfolio policies, see our Editorial Policies and the Editorial Policy Checklist.

Statistics

For all statistical analyses, confirm that the following items are present in the figure legend, table legend, main text, or Methods section.

n/a	Confirmed	
☐	☒	The exact sample size (*n*) for each experimental group/condition, given as a discrete number and unit of measurement
☒	☐	A statement on whether measurements were taken from distinct samples or whether the same sample was measured repeatedly
☐	☒	The statistical test(s) used AND whether they are one- or two-sided<br>*Only common tests should be described solely by name; describe more complex techniques in the Methods section.*
☐	☒	A description of all covariates tested
☐	☒	A description of any assumptions or corrections, such as tests of normality and adjustment for multiple comparisons
☐	☒	A full description of the statistical parameters including central tendency (e.g. means) or other basic estimates (e.g. regression coefficient) AND variation (e.g. standard deviation) or associated estimates of uncertainty (e.g. confidence intervals)
☐	☒	For null hypothesis testing, the test statistic (e.g. *F*, *t*, *r*) with confidence intervals, effect sizes, degrees of freedom and *P* value noted<br>*Give P values as exact values whenever suitable.*
☒	☐	For Bayesian analysis, information on the choice of priors and Markov chain Monte Carlo settings
☐	☒	For hierarchical and complex designs, identification of the appropriate level for tests and full reporting of outcomes
☐	☒	Estimates of effect sizes (e.g. Cohen's *d*, Pearson's *r*), indicating how they were calculated

Our web collection on statistics for biologists contains articles on many of the points above.

Software and code

Policy information about availability of computer code

Data collection	No software was used for data collection.
Data analysis	Softwares:<br>Cutadapt v2.5<br>fastp v0.20.0<br>BBMap/BBNorm<br>Trimmomatic<br>PRINSEQ<br>Sickle v1.33<br>metaSPAdes v3.8.0–3.15<br>MEGAHIT v1.1.3–1.2.9<br>Flye v2.8<br>Racon v1.4.13<br>Medaka v1.0.3<br>Pilon v1.23<br>Minimap2 v2.17<br>Bowtie2 v2.3.5.1<br>BWA-MEM v0.7.12<br>Tiara v1.0.3<br>BBMAP - reformat.sh<br>barrnap v0.9

PRANK v.170427
MMseqs2 v16.747c6
Codetta v2.0.16
hmmscan v3.1b2
Prodigal v2.6.3
blastn v2.9.0
checkV v1.0.3
VIBRANT v1.2.0
Kaiju v1.10.1
Metabuli v1.1.1
DeepMicroClass v1.0.3
makeblastdb v2.9.0
BlastKOALA
MAFFT v7.453
ReAlign-P v1.0.0
IQ-TREE v3.0.1
IQ-TREE v2.6.3
FAMSA v1.2 CPU
R v.4.2.2
ape v.5.3 package
pfam_scan.pl v1.6
BlastKOALA
iNEXT v2.0.22 R package
BMGE v1.12
vegan v2.6.4 R package
picante v1.8.2 R package
tidyverse v2.0.0 R package
RStudio 3.1.446
Fairy v0.5.8
MetaBAT2 v2.2.17
HMMER v3.3
ReAlign-P
inStrain v1.8.0
Custom scripts used within this study were deposited under the following link: https://github.com/MiELevog/A-Global-Mitogenomic-Atlas-Illuminates-Freshwater-Microeukaryote-Diversity. Source data are provided with this paper.

For manuscripts utilizing custom algorithms or software that are central to the research but not yet described in published literature, software must be made available to editors and reviewers. We strongly encourage code deposition in a community repository (e.g. GitHub). See the Nature Portfolio guidelines for submitting code & software for further information.

Data

Policy information about availability of data

All manuscripts must include a data availability statement. This statement should provide the following information, where applicable:

- Accession codes, unique identifiers, or web links for publicly available datasets
- A description of any restrictions on data availability
- For clinical datasets or third party data, please ensure that the statement adheres to our policy

Data availability
All mitogenomes generated within this study have been deposited in the ENA (Project accession: PRJEB95915), as well as in ZENODO (https://doi.org/10.5281/zenodo.20827346). The accession numbers for 3,400 assemblies used are listed in Supplementary Data S1. The 19,925 mitogenome IDs, their accession numbers, Bioproject IDs, and sample IDs, along with the metadata (when available), are provided in Supplementary Data S6.

Databases:
eKOI v1.0.0
RefSeq r226
GTDB r95
pdCEL
Ribosomal Operon Database (ROD)
TIGRFAMs v15.0
SILVA 138.1 SSURef NR99
SILVA 138.1 LSURef NR99
Pfam release 38.0
KEGG database
MitoCOGs

Research involving human participants, their data, or biological material

Policy information about studies with human participants or human data. See also policy information about sex, gender (identity/presentation), and sexual orientation and race, ethnicity and racism.

Reporting on sex and gender	This study does not involve human research participants.

Reporting on race, ethnicity, or other socially relevant groupings	This study does not involve human research participants.
Population characteristics	This study does not involve human research participants.
Recruitment	This study does not involve human research participants.
Ethics oversight	This study does not involve human research participants.

Note that full information on the approval of the study protocol must also be provided in the manuscript.

Field-specific reporting

Please select the one below that is the best fit for your research. If you are not sure, read the appropriate sections before making your selection.

- [ ] Life sciences
- [ ] Behavioural & social sciences
- [x] Ecological, evolutionary & environmental sciences

For a reference copy of the document with all sections, see nature.com/documents/nr-reporting-summary-flat.pdf

Ecological, evolutionary & environmental sciences study design

All studies must disclose on these points even when the disclosure is negative.

Study description

This was an observational, genome-resolved metagenomic study integrating 3,400 publicly available freshwater shotgun metagenomes from 362 lakes spanning all seven continents. The study established a global mitogenome-based framework for generating species-resolved, phylogenetically anchored inventories and recovered approximately 20,000 mitochondrial metagenome-assembled genomes (mitoMAGs).
No experimental treatments or organismal manipulations were performed; therefore, treatment factors, factorial interactions and experimental replicates were not applicable. Depending on the ecological analysis, the principal observational grouping variables were trophic status, season and sampling depth. The study had a hierarchical structure: mitoMAGs were recovered from individual metagenomic samples, which were associated with specific sampling events, depths, sites and lakes. The 3,400 metagenomic samples constituted the primary observational units, whereas individual mitoMAGs were not treated as independent biological replicates. Sample-specific metadata and analysis-specific sample sizes are provided in Supplementary Data S1, the relevant figure legends and the Source Data.

Research sample

Sample type: freshwater.
Sample number: 362.
Origin: 28 countries.
In response to the study's focus on microbial communities within freshwater ecosystems, our research sample comprises an extensive collection of datasets from metagenomic sequencing efforts targeting 28 countries on seven continents. The lakes were meticulously selected to represent a broad spectrum of climatic regions and trophic states (from oligotrophic to eutrophic), ensuring a comprehensive analysis of microbial diversity across different ecological conditions. This strategic selection was motivated by the hypothesis that varying nutrient availability and environmental pressures across these climatic regions and trophic states would influence the diversity and adaptive strategies of the resident microbial populations. The datasets themselves are a compilation of approximately 3'400 environmental metagenomes from which we recovered ~ 20,000 mitochondrial genomes.

Sampling strategy

This study integrates both publicly available metagenomic datasets and metagenomes generated by us, collectively representing inland freshwater lakes and reservoirs across broad ecological and geographical gradients. Sampling sites span multiple countries, with associated lake characteristics (including trophic status), geographic coordinates, and original study information provided in the Supplementary Material. The metagenomes encompass five trophic categories—eutrophic, meso-eutrophic, mesotrophic, oligo-mesotrophic, and oligotrophic—capturing substantial variation in freshwater ecosystem productivity. Samples were commonly processed using size-fractionated filtration with 5-µm and 0.22-µm pore sizes to retain both larger microeukaryotic cells and smaller or fragmented biomass, thereby enabling the recovery of mitochondrial genomes from a wide size range of eukaryotes. Assemblies were generated using either short-read or long-read sequencing technologies, as detailed in Supplementary Data S1, and all datasets were processed through a standardized computational workflow to ensure consistency in downstream analyses.
No a priori sample-size or statistical power calculation was performed because this was an observational, dataset-driven survey rather than an experiment designed to test a prespecified effect size. The final sample size was determined by the availability of eligible freshwater shotgun metagenomic datasets and their suitability for standardized processing and mitochondrial genomic recovery. The resulting collection spans multiple geographical regions, freshwater systems and trophic conditions and was therefore considered sufficient for developing and evaluating the mitochondrial genomic framework presented here.

Data collection

For previously published datasets, the original sampling and data-recording procedures, including the instruments used, are described in the primary studies cited in the Supplementary Information.
LIMNOS datasets. The LIMNOS dataset comprises more than 1,000 metagenomic samples collected and documented by members of the LIMNOS consortium from four lakes in northeastern Germany—Stechlin, Fuchskuhle, Breiter Luzin and Tiefwaren—between 2011 and 2020. Samples were generally collected monthly from the epilimnion of all four lakes and from the hypolimnion of Lake Stechlin. Water samples were processed by successive size-fractionated filtration through 5.0-µm and 0.22-µm membranes. The dataset also includes samples collected between 2004 and 2008, biweekly samples from Lake Stechlin collected in 2020 and water-column profiles from Lake Fuchskuhle collected in 2019.

PELAGICS datasets. The PELAGICS dataset was generated through a coordinated pan-European campaign involving 24 collaborating scientists from 16 countries. Samples were collected and documented by members of the PELAGICS consortium from approximately 70 lakes between 2020 and 2024. Lake identities, trophic-status classifications, sequencing platforms and details of the assembly, polishing and quality-control procedures are provided in the Supplementary Data.
All available information concerning sampling procedures, study sites, sequencing technologies and associated metadata is provided in the Supplementary Information and Supplementary Data.

Timing and spatial scale

The study integrates datasets collected across multiple temporal and spatial scales. For previously published datasets, the start and end dates, sampling frequency, geographic coordinates and site locations are provided for each sampling campaign in the Supplementary Information and associated primary studies.
The LIMNOS dataset comprises several sampling cohorts: legacy samples collected between 2004 and 2008; generally monthly sampling between 2011 and 2020 from the epilimnion of Lakes Stechlin, Fuchskuhle, Breiter Luzin and Tiefwaren and from the hypolimnion of Lake Stechlin; biweekly sampling of Lake Stechlin during 2020; and water-column profile sampling of Lake Fuchskuhle during 2019. The PELAGICS samples were collected between 2020 and 2024 through a coordinated campaign encompassing approximately 70 lakes across 16 European countries. Exact collection dates are provided for each sample in the Supplementary Material.
The spatial scale ranges from within-lake depth profiles and repeated sampling of individual lakes to regional, pan-European and global coverage of inland freshwater lakes and reservoirs. Sample-specific geographic coordinates, sampling depths, site information and, where available, in situ temperature measurements are provided in the Supplementary Material and linked to the corresponding records in the European Nucleotide Archive.

Data exclusions

No data were excluded from the analyses.

Reproducibility

This study was observational and computational and did not involve experimental manipulations. Consequently, no experimental trials were repeated, and successful or failed attempts to repeat experiments are not applicable. Computational reproducibility is supported by the public availability of the input data and generated outputs, together with detailed descriptions of the analytical methods, software versions, parameters and custom scripts used.

Randomization

This is an exploratory study and randomization is not relevant to the study design.

Blinding

Blinding was not performed because it was not relevant to this study. This study was an exploratory survey of microeukaryotic diversity without a priori expectations that would influence the analyses.

Did the study involve field work? ☒ Yes ☐ No

Field work, collection and transport

Field conditions

Metadata describing field conditions are provided in the Supplementary Information. For comprehensive temperature measurements, refer to Table S6 in the Supplementary Dataset. Weather conditions at the time of sampling were typical for each location, and no sampling was conducted during anomalous weather events.

Location

Antarctica -77.43 162.18 Lake Bonney
Australia -27.509 152.3483 Lake Claredon Dam
Austria 47.195119 10.888649 Piburger See
Austria 47.229444 11.013889 Gossenkollesee
Austria 47.557932 13.657765 Hallstatter See
Austria 47.75 13.4 Wolfgang see
Austria 47.806 13.398 Mondsee
Austria 47.806248 13.398749 Mondsee
Austria 47.816667 13.366667 Mondsee
Austria 47.851 13.805 Traunsee
Austria 47.851155 13.805195 Traunsee
Austria 47.865613 13.544841 Attersee
Austria 47.866667 13.533333 Attersee
Austria 47.866667 13.8 Traunsee
Austria 47.86667 13.53333 Attersee
Bolivia -17.4106389 -66.13664167 Laguna Alalay
Brazil -3.533 -61.583 Lago Anama
Canada 43.575988 -81.659668 Lake Huron - Pond 1
Canada 43.628909 -80.047251 Fairy Lake
Canada 43.639494 -65.707216 French Lake
Canada 43.740348 -79.794679 Heart Lake
Canada 43.956985 -79.501228 Eversley Lake
Canada 44.167631 -81.310244 Schmidt Lake
Canada 44.1779 -78.179116 Rice Lake
Canada 44.191192 -66.103933 Clare Lake
Canada 44.204558 -81.052039 Dankert Lake
Canada 44.256065 -64.604625 Spectacle Lake
Canada 44.259513 -80.968908 Copps Lake
Canada 44.261952 -80.641057 Irish Lake

Canada 44.274609 -77.910492 Stevenson Lake
Canada 44.373545 -64.575777 Wiles Lake
Canada 44.392148 -76.818354 Varty Lake
Canada 44.449413 -65.86015 Everitts Lake
Canada 44.467555 -78.695518 Kawartha Lakes
Canada 44.494194 -76.413815 Upper Rock Lake
Canada 44.517504 -65.495287 Lake Mulgrave
Canada 44.544714 -75.998585 Charleston Lake
Canada 44.603322 -76.471579 Christie Lake
Canada 44.603455 -79.506646 Bass Lake 2
Canada 44.651541 -63.497262 Morris Lake
Canada 44.655567 -63.469954 Bissett Lake
Canada 44.687068 -79.325113 Lake St John
Canada 44.692693 -63.553527 Lake Micmac
Canada 44.722618 -63.551142 Lake Charles
Canada 44.725929 -81.227067 Boat Lake
Canada 44.745336 -63.785165 Halfway Lake
Canada 44.969839 -64.555716 Gaspereau Lake
Canada 45.018778 -64.59139 Tupper Lake
Canada 45.035782 -76.184422 Mississippi Lake
Canada 45.216979 -81.55796 Cameron Lake
Canada 45.219083 -63.31812 Shortts Lake
Canada 45.257686 -74.702097 Loch Garry
Canada 45.291223 -66.056773 Lily Lake
Canada 45.316199 -66.760412 McDougall Lake
Canada 45.415281 -65.968124 Ritchie Lake
Canada 45.456735 -72.205891 Lac Brais
Canada 45.522507 -61.560681 Sheperd Lake
Canada 45.564777 -77.04943 Mink Lake
Canada 45.622179 -61.362123 Embrees Pond
Canada 45.689327 -70.919835 Lac des Trois Milles
Canada 45.70964 -67.188989 Magaguadavic Lake
Canada 45.778379 -72.003987 Burbank Pond
Canada 45.876582 -81.981245 Pike Lake
Canada 45.87684 -75.957058 Manitou Lake
Canada 45.87875 -82.886544 Silver Lake
Canada 45.892125 -74.028641 Echo Lake
Canada 45.931439 -64.368197 Silver Lake
Canada 45.948123 -74.143544 Rond Lake
Canada 45.966328 -74.301609 Theodore Lake
Canada 46.084681 -71.507629 Lac a la truite
Canada 46.110564 -64.150711 Collins Lake
Canada 46.119508 -70.853652 Lake Fortin
Canada 46.132703 -61.185324 Lake Ainslie
Canada 46.136558 -70.809503 Lake Volet
Canada 46.170166 -60.014699 Reserve Mines - Pond 1
Canada 46.171199 -60.318258 Johnson Lake
Canada 46.238761 -62.883752 Lake Verde
Canada 46.347667 -62.528109 Whitlocks Pond
Canada 46.42853 -62.419913 Larkins Pond
Canada 46.480215 -80.973773 Ramsey Lake
Canada 46.635064 -65.117567 Kent Lake
Canada 46.796823 -72.522866 Lac des Chicots
Canada 46.89213 -65.726518 Crocker Lake
Canada 46.896289 -67.430162 Sisson - Pond 1
Canada 46.921603 -67.657604 Lake Edward
Canada 46.934091 -67.761037 Piries Lake
Canada 46.949699 -67.537685 Blue Bell Lake
Canada 47.010232 -66.967572 Trousers Lake
Canada 47.623501 -52.825599 Millers Pond
Canada 47.694086 -67.642841 Eightmile Lake
Canada 47.702298 -68.588895 Grand Lac Squatec
Canada 47.946561 -69.226091 Bertrand Lake
Canada 48.213133 -65.852159 Lac Ã l'oie
Canada 48.214755 -79.052164 Pelletier Lake
Canada 48.266623 -68.864121 Grand lac Malobes
Canada 48.284702 -68.869635 Lac de la Station
Canada 48.290487 -68.405921 Neigette Lake
Canada 48.358443 -68.152578 Des Joncs Lake
Canada 48.40963 -71.78992 Vouzier Lake
Canada 48.437398 -123.522473 Glen Lake
Canada 48.448187 -123.529102 Langford Lake
Canada 48.600521 -67.820387 Michaud Lake
Canada 48.609742 -67.796449 Antoine Lake
Canada 48.673607 -67.734732 Desire Lake
Canada 48.714034 -64.76873 Bazire Lake

Canada 48.714643 -123.613474 Dougan Lake
Canada 48.949934 -55.693434 Grand Falls-Windsor - Pond 1
Canada 49.008293 -122.35186 Laxton Lake
Canada 49.011402 -113.456511 Outpost Lake
Canada 49.044741 -122.310811 Mill Lake
Canada 49.062317 -100.141832 Max Lake
Canada 49.070025 -123.811737 Quennell Lake
Canada 49.112245 -81.03531 Lillabelle Lake
Canada 49.187271 -80.971159 Dora Lake
Canada 49.203775 -124.014124 Diver Lake
Canada 49.214698 -124.055142 Brannen Lake
Canada 49.217733 -105.851938 Fife Lake
Canada 49.230739 -124.060791 Green Lake
Canada 49.230934 -95.682275 Whitemouth Lake
Canada 49.236235 -122.97178 Deer Lake
Canada 49.243327 -122.946538 Burnaby Lake
Canada 49.288777 -119.531587 Vaseux Lake
Canada 49.289097 -119.582084 Mahoney Lake
Canada 49.302656 -119.571005 Green Lake
Canada 49.410038 -119.585408 Skaha Lake
Canada 49.443092 -122.020294 Chehalis Lake
Canada 49.559465 -124.814538 Chicadee Lake
Canada 49.636743 -125.01777 Maple Lake
Canada 49.667268 -110.29574 Elkwater Lake
Canada 49.67651 -117.658481 Upper Little Slocan Lake
Canada 49.688665 -93.747517 Teggau Lake - Pond 3
Canada 49.690256 -93.717327 Teggau Lake - Pond 2
Canada 49.695238 -124.507497 Cranby Lake
Canada 49.698298 -93.70868 Teggau Lake - Pond 1
Canada 49.711052 -83.684753 Johnsons Lake
Canada 49.715267 -119.923178 Munro Lake
Canada 49.787686 -94.463764 Rabbit Lake
Canada 49.829004 -119.839318 Silver Lake
Canada 49.839629 -102.425965 Gillis Lake
Canada 49.856847 -119.612241 Shannon Lake
Canada 49.89478 -120.31 Elkhart Lake
Canada 49.896614 -120.564326 Kentucky Lake
Canada 49.915259 -120.2769 Paradise Lake
Canada 49.986539 -107.935004 Pelletier Lake
Canada 50.034106 -74.216565 Winsch Lake
Canada 50.110319 -108.496977 Gull Lake
Canada 50.114032 -122.981403 Alta Lake
Canada 50.149717 -120.23787 Douglas Lake
Canada 50.151407 -122.931788 Green Lake
Canada 50.166435 -120.528181 Nicola Lake
Canada 50.172812 -119.327375 Kalamalka Lake
Canada 50.204665 -66.179286 Shakaikaniss Lake
Canada 50.215541 -122.490763 Lillooet Lake
Canada 50.318415 -119.256066 Swan Lake
Canada 50.388589 -100.626164 Shoal Lake
Canada 50.393416 -109.488322 Fox Valley - Pond 1
Canada 50.401284 -91.254248 Rob Lake
Canada 50.403098 -121.085132 Pimainus Lake
Canada 50.411549 -119.250032 Otter Lake
Canada 50.450374 -127.400686 Alice Lake
Canada 50.472208 -120.152128 Roche Lake
Canada 50.474369 -97.409451 Norris Lake
Canada 50.485507 -120.50673 Shambrook Lake
Canada 50.536695 -119.499488 Bolean Lake
Canada 50.542953 -106.012079 Pelican Lake
Canada 50.593529 -119.639537 Pillar Lake
Canada 50.621082 -120.049718 Upper Buse Lake
Canada 50.643861 -122.401882 Anderson Lake
Canada 50.718456 -71.765768 Froget Lake
Canada 50.859036 -121.053029 Cultus Lake
Canada 50.876894 -119.313467 Little White Lake
Canada 50.888723 -98.313076 Lake Pine
Canada 50.903076 -98.035665 Steepbank Lake
Canada 51.087067 -56.861193 Otter Pond
Canada 51.097401 -120.34108 Whitewood Lake
Canada 51.316192 -102.87704 Wilson Lake
Canada 51.473574 -123.887461 Konni Lake
Canada 51.591362 -113.752929 Stirlingville - Pond 1
Canada 51.734097 -121.352237 108 Mile Lake
Canada 51.83278 -120.388941 Italia Lake
Canada 52.003037 -108.784517 Tramping Lake

Canada 52.011725 -125.008155 Clearwater Lake
Canada 52.320231 -109.950294 Macklin Lake
Canada 52.398519 -98.912933 Devils Lake
Canada 52.638048 -109.898255 Suffern Lake
Canada 52.645011 -103.306995 Saskatchewan - Pond 2
Canada 52.670496 -109.211926 Schmidt Lake
Canada 52.70846 -109.791027 Manitou Lake - Pond 1
Canada 52.722421 -105.20894 Basin Lake - Pond 1
Canada 52.738966 -109.652625 Manitou Lake
Canada 52.844297 -108.858587 Atton Lake
Canada 52.91311 -107.879068 Acton Lake
Canada 52.94939 -122.420822 Dragon Lake
Canada 52.979083 -107.813473 ge sneachta
Canada 53.037201 -108.278678 Murray Lake
Canada 53.089241 -107.236949 Mistawasis Lake
Canada 53.110315 -111.699187 Thomas Lake
Canada 53.183739 -106.844945 Saskatchewan - Pond 1
Canada 53.189089 -107.659168 Meeting Lake
Canada 53.256011 -112.912167 Miquelon Lake
Canada 53.42795 -124.545783 Brewster Lakes
Canada 53.449373 -116.779894 Fickle Lake
Canada 53.463352 -113.228239 Drader's Lake
Canada 53.543962 -109.095452 Spruce Lake - Pond 1
Canada 53.560254 -114.321126 Clear Lake
Canada 53.607447 -123.488697 Naltesby Lake
Canada 53.623604 -113.95501 Muir Lake
Canada 53.682958 -116.270249 Near McLeod River - Pond 1
Canada 53.810107 -125.913779 Eakin Lake
Canada 53.875399 -124.755299 Tahultzu Lake - Pond 1
Canada 53.924085 -107.702873 Poplar Ridge Lake
Canada 53.938458 -125.907128 Tatalaska Lake
Canada 53.939715 -114.321366 Lake La Nonne
Canada 53.977642 -125.898396 Francois Lake - Pond 1
Canada 54.008382 -123.135854 Reid Lake
Canada 54.077005 -125.757675 Mulvaney Lake
Canada 54.186152 -125.428674 Co-op Lake
Canada 54.242465 -125.061656 Hanson Lake
Canada 54.285684 -104.640543 Little bear lake - Pond 1
Canada 54.37048 -101.457133 Egg Lake
Canada 54.381312 -128.559744 Lakelse Lake
Canada 54.483 -112.754406 Lake Long
Canada 54.574531 -123.807885 Lake Fraser-Fort George
Canada 54.646202 -111.652296 Lake Elinor
Canada 54.656922 -126.924746 Lake Round
Canada 54.934739 -127.55412 Lake Kitseguecla
Canada 54.993669 -108.344502 Saskatchewan - Pond 1
Canada 55.061409 -114.016738 Lesser Slave River No.124
Canada 55.120772 -125.196624 Lake Takatoot
Canada 55.217558 -119.091379 Lake Saskatoon
Canada 55.22516667 -77.69711111 Near Hudson-bay - Pond 6
Canada 55.22566667 -77.69716667 Near Hudson-bay - Pond 5
Canada 55.22611111 -77.69647222 Near Hudson-bay - Pond 4
Canada 55.22641667 -77.69572222 Near Hudson-bay - Pond 3
Canada 55.22680556 -77.69444444 Near Hudson-bay - Pond 1
Canada 55.22680556 -77.69561111 Near Hudson-bay - Pond 2
Canada 55.445634 -129.353516 Lake Kitimat-Stikine
Canada 55.446225 -129.334999 Lake Kitimat-Stikine
Canada 55.628822 -118.528498 Lake Kakut
Canada 55.871434 -117.173848 Lake Magloire
Canada 56.240918 -117.734668 Lake Cardinal
Canada 56.462556 -111.162013 Lake Gregoire
Canada 57.292089 -111.238794 Lake Kearl
Canada 58.21637 -129.837323 Upper Gnat Lake
Canada 58.935329 -129.954645 Lake Kitimat-Stikine
Canada 59.300477 -129.276001 Lake Good Hope
Canada 59.534902 -112.216745 Lake Pine
Canada 59.703067 -133.60852 Lower McDonald Lake
Canada 59.761821 -133.773316 Cliff Lake
Canada 59.798928 -122.523299 Lake Coles - Pond 1
Canada 59.944209 -111.853586 Lake Four Mile
Canada 59.979224 -127.55746 Near Liar river pond 1
Canada 60.062737 -128.680252 Lake Second Wye
Canada 60.063643 -128.699031 Lake Wye
Canada 60.063822 -116.847789 Great Slave lake - Pond 5
Canada 60.080432 -128.745812 Lake Watson - Pond 1
Canada 60.107692 -128.80679 Lake Watson

Canada 60.125379 -130.926778 Lake Pine
Canada 60.322662 -137.015561 Lake Klukshu
Canada 60.450307 -134.265322 Lake Marsh - Pond 1
Canada 60.53671 -114.568217 Great Slave lake - Pond 4
Canada 60.596644 -116.262865 Great Slave lake - Pond 3
Canada 60.721344 -114.996196 Great Slave lake - Pond 2
Canada 60.813148 -114.589631 Great Slave lake - Pond 1
Canada 60.836026 -116.656975 Lake Heart
Canada 61.455898 -130.240718 Lake Wolverine
Canada 61.890891 -116.542274 Chan Lake
Canada 61.930612 -116.520168 Near Birsh Lake - Pond 2
Canada 61.934997 -132.521865 Lake Jackfish
Canada 61.998774 -116.330501 Near Birsh Lake - Pond 1
Canada 62.187056 -134.685124 Little salmon lake - Yukon
Canada 62.256408 -140.66822 Lakes Enger
Canada 62.556145 -116.433386 Near Mackenzie River - Pond 5
Canada 63.809838 -137.894084 Lake Gravel
Canada 67.381817 -134.040849 Near Mackenzie River - Pond 4
Canada 67.417758 -133.922557 Near Mackenzie River - Pond 3
Canada 67.432054 -134.851562 Near Mackenzie River - Pond 2
Canada 67.468001 -133.763637 Chii Echeii Van
Canada 68.232838 -133.44037 Lake Boot
Canada 68.350907 -133.708655 Lake Boot
China 39.11049 117.7274 Chaobai River
China 39.15497 117.6609 Chaobai River
China 39.15863 117.6434 Chaobai River
China 39.27763 117.5778 Chaobai River
China 39.38984 117.5044 Chaobai River
China 39.47033 117.479 Chaobai River
China 39.60959 117.3882 Chaobai River
China 39.67972 117.2903 Chaobai River
China 39.71022 117.2088 Chaobai River
China 39.73796 117.1294 Chaobai River
China 39.76123 117.1858 Chaobai River
China 39.78488 116.9725 Chaobai River
China 39.85734 116.8424 Chaobai River
China 39.90604 116.7613 Chaobai River
China 39.90758 116.7813 Chaobai River
China 39.97084 116.7628 Chaobai River
China 40.04623 116.7651 Miyun Reservoir
China 40.1023 116.7319 Miyun Reservoir
China 40.12949 116.6711 Miyun Reservoir
China 40.33768 116.7958 Miyun Reservoir
China 40.34758 116.8168 Miyun Reservoir
China 40.34765 116.8369 Miyun Reservoir
China 40.36955 116.8359 Miyun Reservoir
China 40.39061 116.9106 Miyun Reservoir
China 40.40766 116.8471 Miyun Reservoir
China 40.43775 116.9959 Miyun Reservoir
China 40.61254 116.7956 Miyun Reservoir
China 40.63165 116.775 Miyun Reservoir
China 40.65039 117.1777 Miyun Reservoir
China 40.65438 116.775 Miyun Reservoir
China 40.68011 117.1267 Chaohe River
China 40.694 117.1617 Chaohe River
China 40.72984 116.6237 Chaohe River
Croatia 43.857811 15.980253 Visova⑤}ko jezero
Croatia 43.883333 15.566667 Lake Vrana
Croatia 44.85 14.383333 Lake Vrana
Croatia 44.860611 15.600525 Plitvi⑤}ki Ljeskovac
Croatia 44.888253 15.606771 Plitvi⑤}ka Jezera
Czech Republic 48.5 14.29 Bad Leonfelden - Pond 1
Czech Republic 48.618919 14.672049 Jiricky rybnik Pond
Czech Republic 48.7775469 13.8676142 Plesne jezero Lake
Czech Republic 48.846361 14.487639 Rimov Reservoir
Czech Republic 48.848516 14.490728 Rimov Reservoir
Czech Republic 48.916292 14.88674 Cep I Lake
Czech Republic 49.0225 15.241667 Vodni Nadrz Landstejn Lake
Czech Republic 49.1652458 13.1998789 Certovo jezero Lake
Czech Republic 49.1812253 13.1857425 Cerne jezero Lake
Czech Republic 49.7660478 14.4149044 Slapy Reservoir Lake
Czech Republic 50.066111 13.932222 Udolni nadrz Klicava
Czech Republic 50.07 13.93 Udolni nadrz Klicava
Czech Republic 50.088333 13.127778 Vodni nadrz Zlutice
Czech Republic 50.09 13.13 Vodni nadrz Zlutice
Czech Republic 50.179408 12.587602 Lake Medard

Czech Republic 50.179564 12.584932 Lake Medard
Czech Republic 50.516667 13.616667 Lake Matylda
Czech Republic 50.536944 13.644444 Lake Mostecke jezero
Czech Republic 50.539746 13.645342 Lake Mostecke jezero
Czech Republic 50.633333 13.933333 Lake Milada
Czech Republic 50.653611 13.944444 Lake Milada
Czech Republic 50.654974 13.948885 Lake Milada
Estonia 57.931389 27.342222 Lake Mustjarv
Estonia 57.940833 27.346667 Lake Valgojarv
Estonia 58.283333 26.033333 Lake Vortsjarv
Estonia 58.676667 27.491667 Lake Lahepera
Finland 61.07666667 25.03277778 Lake Lovonjarvi
Finland 61.20805556 25.11388889 Lake Hameenlinna
Finland 61.21611111 25.21555556 Lake Rajajarvet
Finland 61.21805556 25.2125 Lake Rajajarvet
Finland 61.23083333 25.14222222 Lake Miekkojarvi
Finland 61.24222222 25.06305556 Lake Valkea-Kotinen
France 45.495647 2.88759 Lac Pavin
France 45.664187 2.986085 Lac d'Aydat
France 48.608669 1.938444 Bullion - Pond 1
France 48.688806 1.916306 Auffargis - Pond 1
Germany 53.105421 12.984514 Grosser Fuchskuhlen See
Germany 53.105659 12.984807 Grosser Fuchskuhlen See
Germany 53.105818 12.984839 Grosser Fuchskuhlen See
Germany 53.106095 12.985021 Grosser Fuchskuhlen See
Germany 53.1427 13.017808 Lake Stechlin
Germany 53.153235 13.02703 Lake Stechlin
Germany 53.15403 13.01311 Lake Stechlin
Germany 53.155403 13.031352 Lake Stechlin
Germany 53.356995 13.465993 Lake Breiter Luzin
Germany 53.358636 13.466862 Lake Breiter Luzin
Germany 53.527308 12.691058 Lake Tiefwaren
Germany 53.529496 12.691925 Lake Tiefwaren
Greece 38.563333 21.558611 Lake Trichonida
Greece 39.656111 20.891389 Lake Pamvotida
Greece 40.526944 21.286111 Lake Orestiada
Greece 40.664167 23.531389 Lake Volvi
Hungary 46.833333 17.733333 Lake Balaton
Hungary 47.216667 18.616667 Lake Velence
Hungary 47.816667 16.75 Lake Neusiedl
Italy 45.615644 10.633534 Lago di Garda
Italy 45.816667 8.4 Lago d'Orta
Italy 45.966667 8.65 Lago Maggiore
Italy 45.983333 8.966667 Lago di Lugano
Italy 46.009123 9.021579 Lago di Lugano
Japan 31.233333 130.566667 Lake Ikeda
Japan 35.219306 135.99575 Lake Biwa
Kyrgyzstan 42.36926 76.5013 Issyk Kul
Kyrgyzstan 42.36927 76.85868 Issyk Kul
Kyrgyzstan 42.520266 76.70319 Issyk Kul
Kyrgyzstan 42.52367 77.88886 Issyk Kul
Kyrgyzstan 42.58698 78.07565 Issyk Kul
Kyrgyzstan 42.60604 78.21927 Issyk Kul
Kyrgyzstan 42.69569 78.08699 Issyk Kul
Kyrgyzstan 42.72432 78.14245 Issyk Kul
Kyrgyzstan 42.74128 78.29785 Issyk Kul
Kyrgyzstan 43.3956 77.10314 Issyk Kul
Netherlands 52.061203 5.160367 Plas Vechten Lake
Netherlands 52.566667 5.216667 Markermeer
Poland 53.662246 21.89935 Lake Ros
Poland 53.778889 21.454444 Lake Jezioro Majcz Wlk
Poland 53.821461 21.404498 Lake Jezioro Kuc
Poland 53.875833 21.567778 Lake Jezioro Taltowisko
Poland 53.9 21.666667 Lake Jezioro Szymoneckie
Puerto Rico 18.33333333 -66.23666667 Lago La Plata
Romania 45.6925 22.859972 Lacul Cincis
Romania 46.125833 25.886972 Saint Anna Lake
Romania 46.664583 23.052472 Lake Belis-Fantanele
Romania 46.720139 23.277694 Lake Tarnita
Romania 46.966444 23.90025 Lacul Stiucilor
Rwanda -1.64 29.05 Lake Kivu
Rwanda -1.78 29.2 Lake Kivu
Spain 38.535 -0.266528 Embalse de Amadorio
Spain 39.133333 -0.65 Embalse de Tous
Spain 39.987935 -1.873691 Laguna del Tejo
Spain 39.99351 -2.14144 Arcas - Pond 1

Spain 42.125278 2.755278 Lake of Banyoles
Spain 42.5816 1.0031 Estany de Sant Maurici
Sweden 57.93305556 15.55166667 Lake Stortoveln
Sweden 57.93361111 15.57222222 Lake Glimmingen
Sweden 58.34027778 16.20388889 Lake Skiren
Sweden 58.4 14.6 Lake Vattern
Sweden 58.8 13.333333 Lake Vattern
Sweden 59.433553 17.067261 Lake Malaren
Sweden 59.64222222 16.14361111 Lake Vastersjon
Sweden 59.76888889 18.64277778 Lake Malstasjon
Sweden 59.7975 18.50611111 Lake Fyrsjon
Sweden 59.83638889 18.64194444 Lake Erken
Sweden 59.846171 18.575602 Lake Erken
Sweden 59.8625 18.5425 Lake Platen
Sweden 63.34916667 14.45888889 Lake Krokom municip
Sweden 63.38569444 14.45580556 Lake Krokom municip
Sweden 64.12311111 18.7805 Lake Orealven
Sweden 64.15038889 18.79975 Lake Orealven
Sweden 67.65141667 21.16713889 Lake Luongasjarvi
Sweden 67.92330556 20.36911111 Lake Jukajarvi
Sweden 67.92872222 20.36125 Lake Jukajarvi
Sweden 68.316667 19.283333 Lake Tornetrask
Switzerland 45.966 8.65 Lago Maggiore
Switzerland 46.009 9.021 Lago di Lugano
Switzerland 46.550276 8.711588 Lake Cadagno
Switzerland 46.683333 7.716667 Lake Thun
Switzerland 46.683334 7.716668 Lake Thun
Switzerland 46.683 7.716 Lake Thun
Switzerland 47.01861111 6.996666667 Le Loclat
Switzerland 47.300659 8.578777 Lake Zurich
Switzerland 47.349938 8.679344 Greifensee
Switzerland 47.366667 8.7 Greifensee
Switzerland 47.3 8.566667 Lake Zurich
Switzerland 47.3 8.57 Lake Zurich
Switzerland 47.633333 9.366667 Bodensee
Tanzania -4.84 29.61 Lake Tanganyika
Tanzania -4.89 29.586 Lake Tanganyika
Tanzania -4.89 29.59 Lake Tanganyika
Tanzania -4.91 29.49 Lake Tanganyika
Tanzania -5.58 29.51 Lake Tanganyika
Tanzania -5.74 29.92 Lake Tanganyika
Tanzania -6.18 29.66 Lake Tanganyika
Tibet 28.970151 90.512947 Lake Chenco
Tibet 30.621636 93.188717 Jia Naiyucuo
USA 43.0995 -89.4045 Lake Mendota
USA 43.099 -89.405 Lake Mendota
USA 46.0412 -89.6861 Trout bog lake
USA 68.63333333 -149.6 Lake Toolik

Access & import/export

The PELAGICS dataset was generated through a coordinated pan-European sampling campaign conducted in collaboration with in-country partners from each participating nation, ensuring local access permissions, logistical coordination, and compliance with national regulations. The LIMNOS dataset was derived from sampling in German lakes, with fieldwork performed by the Leibniz Institute of Freshwater Ecology and Inland Fisheries (IGB). All sampling activities were carried out in conformity with applicable laws and permitting requirements, and no material was collected or transferred without the involvement of responsible partners in the country of origin. Responsibility for the LIMNOS sampling and associated data generation lies with Hans-Peter Grossart, and responsibility for the PELAGICS sampling campaign lies with Rohit Ghai.

Disturbance

No disturbances were caused by the sampling procedures.

Reporting for specific materials, systems and methods

We require information from authors about some types of materials, experimental systems and methods used in many studies. Here, indicate whether each material, system or method listed is relevant to your study. If you are not sure if a list item applies to your research, read the appropriate section before selecting a response.

Materials & experimental systems

n/a	Involved in the study
☒	☐ Antibodies
☒	☐ Eukaryotic cell lines
☒	☐ Palaeontology and archaeology
☒	☐ Animals and other organisms
☒	☐ Clinical data
☒	☐ Dual use research of concern
☒	☐ Plants

Methods

n/a	Involved in the study
☒	☐ ChIP-seq
☒	☐ Flow cytometry
☒	☐ MRI-based neuroimaging

Plants

Seed stocks	This study does not involve plant samples.
Novel plant genotypes	This study does not involve plant samples.
Authentication	This study does not involve plant samples.
