# Supplementary material for: A global mitochondrial genomic atlas illuminates freshwater microeukaryote diversity
Source: Nat Commun. 2026 Sep 25;17:11139. doi: 10.1038/s41467-026-78160-1 (PMC13710963; doi:10.1038/s41467-026-78160-1)
Supplement: Supplementary file 4 [file 41467_2026_78160_MOESM5_ESM.pdf]

nature portfolio

# Peer Review File

# A Global Mitochondrial Genomic Atlas Illuminates Freshwater Microeukaryote Diversity

Corresponding Author: Dr Adrian-Stefan Andrei

**This file contains all reviewer reports in order by version, followed by all author rebuttals in order by version.**

Version 0:

Reviewer comments:

Reviewer #1

(Remarks to the Author)
The manuscript entitled “A Global Mitochondrial Genomic Atlas Illuminates Freshwater Microeukaryote Diversity” represents a timely and important contribution to our understanding of microbial eukaryotic diversity and ecology in freshwater systems. The authors take advantage of large-scale metagenomic datasets that have traditionally been explored primarily for prokaryotic diversity, instead using organellar (mitochondrial) genomes to access eukaryotic diversity. This is a powerful idea that helps bridge a longstanding gap caused by the difficulty of recovering nuclear eukaryotic genomes from metagenomic data, a limitation that has led to microbial eukaryotes being underexplored in many datasets.

While the general concept of leveraging mitochondrial genomes from metagenomes has been developing over the past several years, both conceptually and methodologically, this study substantially extends previous efforts by applying the approach at an unprecedented scale and with a specific focus on freshwater lakes. The resulting atlas convincingly demonstrates the potential of mitogenomic data to reveal previously uncharacterized diversity and ecological patterns, and it clearly advances our understanding of freshwater microeukaryotes.

Overall, we find this manuscript to be very interesting and well written, with clear potential for significant impact. The work represents a substantial technical and conceptual advance, and the dataset assembled here will likely be a valuable resource for the community. That said, there are several points where additional clarification, discussion, or quantitative support would further strengthen the manuscript and help readers better interpret the scope and limitations of the approach.

Major comments

1. While many recovered mitochondrial genomes can be confidently placed within higher-level clades, the lack of comprehensive mitochondrial reference databases clearly limits taxonomic assignment at lower ranks. This important limitation would benefit from a more explicit discussion. For example, how many of the recovered mitochondrial genomes could be assigned a species name? Quantifying this would help readers better assess the current limits of taxonomic resolution imposed by reference database gaps.

2. Relative abundances are central to the ecological analyses in this study. The authors propose an innovative approach based on mitochondrial genomes, using cluster size as a proxy for relative abundance. While this is an interesting and promising idea, some additional clarification and discussion of potential biases (e.g., effects of mitochondrial copy number, genome fragmentation, or assembly success) would be helpful, as this approach is relatively new.

3. An important question concerns how well the pipeline performs for non-canonical mitochondrial genomes, including fragmented, linear, highly reduced, or otherwise atypical architectures, which are relatively common among microbial eukaryotes. The manuscript touches on this only briefly. It would be valuable to know whether any such atypical mitochondrial genomes were recovered in the dataset, and how they behaved during filtration and clustering. In addition, the literature cited on reduced mitochondrial genomes appears somewhat outdated.

4. To recover mitoginoomes authors used an approach based on multilayer filtering of the candidate mitoginomes, followed by clustering based on an empirically inferred species-level threshold. This is an interesting and pragmatic solution, but it raises some concerns. In particular, if a single mitochondrial genome is split into two or more non-overlapping fragments,

these fragments would not cluster and could be represented as separate mitogenomes in the atlas. Perhaps that is addressed by the search for conserved genes. Some discussion of how frequently this might occur, and how it could affect downstream diversity estimates, would be useful.

5. Following that comment, we think that more systematic statistics describing the recovered mitogenomes, such as size distributions, GC content, and gene content across major groups, ideally in comparison to their closest known relatives is missing. While some of this information is available in supplementary materials, bringing a clearer summary into the main text would strengthen the manuscript. Such metrics could also serve as proxies for genome completeness, which is particularly important given that many recovered mitogenomes appear to be partial. Clarifying how complete a mitochondrial genome needs to be to support species-level or ecological conclusions would help contextualise the results.

6. The species delineation strategy is innovative and potentially very powerful. However, the calibration dataset used to infer species-level thresholds is described as being biased toward multicellular eukaryotes. Providing more quantitative information on the taxonomic composition of this dataset (e.g., distribution across major eukaryotic supergroups) would help readers better evaluate how transferable the threshold is to various groups of microbial eukaryotes.
7. The phylogenetic tree is presented at a relatively general level. Several large clades appear to be dominated by newly recovered mitochondrial genomes, including within Metazoa. Commenting in more detail on what kinds of taxa or lineages this “dark diversity” may represent would be useful.

Minor comments

Lines 294–303 focus on Geminigeraceae mitochondrial genomes. While this is interesting, discussing a single, specific group in such detail is in contrast with the otherwise large-scale perspective of the study. A clearer motivation for highlighting this particular case, or a brief indication of its broader relevance, would help it fit more naturally into the manuscript.

Line 420: Metazoan mitogenomics per se is well established, but environmental metazoan mitogenomics remains an actively developing field. Providing a bit more context and citing more recent studies would help place the present work in a broader perspective, particularly given that this study also encompasses some metazoan diversity.

Supplementary Table S6: Units are missing for the size and GC content columns.

In summary, this is a very strong and exciting manuscript that convincingly demonstrates the power of mitochondrial genomes for exploring (freshwater) microeukaryotic diversity across scales. Addressing the points above, particularly those related to genome completeness, taxonomic resolution, and methodological limitations, would further strengthen the paper.

Reviewer #2

(Remarks to the Author)

My name is Jeremy Wideman, I am an Associate Professor in the Center for Mechanisms of Evolution at Arizona State University. I have published several papers on mtDNAs on protists and though I am not an ecologist, I believe I am capable of providing feedback on this manuscript.
I am happy for the authors to contact me if they have any questions about this review.
The manuscript by Moncadas et al. presents a global atlas of freshwater mitochondrial genomes. The scope of the paper is very large, and a lot of data has been analyzed. I think that there is some excellent work done here, but given the presentation, it is difficult for me to assess what kinds of genomes have been identified and if the Atlas is relatively complete or is extremely biased towards only the most common freshwater organisms (algae and perhaps certain parasites like oomycetes).
I have made several suggestions that will help me to understand the completeness of the data and whether it is truly an atlas, or more of a compendium of the most common species/lineages. Even if the Atlas is lacking, the work is still important, however, to maximize the utility of the dataset for other scientists, more information and better clarity on the taxa actually recovered is necessary.

I cannot comment on ecology apart from, yes! mtDNAs are useful for ecology!
Thus, my comments are largely limited to Figure 3 and the associated supplementary data.

MAJOR Comments:

1. The authors use the term "mitochondrial genome" where they mean "mitochondrial contig". The mean length of ~9kb for a protist mtDNA is misleading. This is the mean contig length that you have ascribed to being part of mtDNA.
How many complete, circular mapping, mtDNAs do you recover?
The authors need to make clearer what it is they are reporting. State the # of mitochondrial genome contigs, the number of unique novel circular mapping mtDNAs.

2. The authors state that they increase the number of SAR clade mtDNA representatives. SAR is a massive group. This is like saying that you recovered X# of either plant, animal, or fungal mtDNAs without specifying how many of each.
To address this issue, trees need to have more taxa identified from every group. At the moment, I cannot assess how much

diversity has actually been recovered.
There are MANY things that can be done to improve the readability of the trees so that readers can assess the true impact of this work.
ALSO: for any further review of this paper I require access to the data including a tree with readable names.

At MINIMUM please make the following inferable from the tree (and split figure 3a accordingly as well):
A. Stramenopiles: where are the ochrophytes vs oomycetes vs the rest of the stramenopiles? If you ONLY recovered photosynthetic stramenopiles, then the dataset isn't as interesting.
B. Alveolates: Distinguish between dinoflagellates, apicomplexans,ciliates. This helps readers understand what kind of diversity you actually obtained for this group.
C. Rhizaria: Unclear what you have included or not in the dataset.
D. Animals and Fungi: what groups have been recovered? Label major phyla
E. Sometimes there is a yellow line amidst a bunch of blue, these seeming orphans need to be named. If the COIs/16S branches are well supported, then these "unknown groups" become known.
F. Cryptists and haptists include several lineages of heterotrophs. It SEEMS like this paper has mostly recovered phototrophs. BUT this is unclear from the way the data are presented. Please articulate in the trees where phototrophic vs heterotrophic lineages are.
G. What discobids are in the tree?
H. The COI proteins for any putative "unknown lineage" should be used as BLAST queries into a mitochondrial database that includes ALL published mitochondrial genomes. This might be difficult, but it is important to know if you have found a new lineage or if the database you are basing your inferences off of is severely incomplete.

3. A COI tree and a SSU/LSU tree is convenient, but given that mitochondrial genomes are being recovered, a larger tree with more subunits could be generated. Inclusion of COB at least would increase resolution.
Similarly, trees with this many branches are subject to artefact, is it necessary to include so many branches? some degree of identity could be used to simplify the tree.
ALSO: why not include branch lengths? A cladogram is only somewhat useful.
Why are the trees rooted on Stramenopiles?
A stripped down concatenated tree using ALL electron transport chain proteins regularly found in the mtDNA could help resolve your tree a bit better and identify any new lineages. Our Butenko 2024 paper in BMC biology should have all the necessary sequences. Of course, you would need to drastically decrease the number of taxa included in your tree.

4. Do you have a sense of your false negative rate? Many "NEW" mtDNAs that we discover in our lab hit mtDNAs and bacterial sequences with similar BLAST evalues. It can be somewhat difficult to be certain. AND MANY of our most interesting discoveries would NEVER be picked up by any automated system. So, I'm curious. How certain are you of your rejected contigs?

5. The 748 unclassifiable mtDNAs should be manually checked, especially any that are complete or near complete. This will help understand how many new lineages you may have identified.

Reviewer #3

(Remarks to the Author)
I co-reviewed this manuscript with one of the reviewers who provided the listed reports. This is part of the Nature Communications initiative to facilitate training in peer review and to provide appropriate recognition for Early Career Researchers who co-review manuscripts.

Reviewer #4

(Remarks to the Author)
This paper explores a large dataset of mitogenomes from protists occurring in lakes. Generating this dataset and analysing it has substantial merit. One of the main and most interesting findings is the new diversity of mitogenomes across several protist groups that were previously underrepresented.

Main comments:

The work claims to be “global”, while the vast majority of samples come from North America and Europe (Fig1). This should be rephrased in the title and the rest of the manuscript. E.g., there seem to be a couple of samples from the entire South American continent, a few from Africa, and one or a handful from Australia. The dataset is quite impressive as it is, but it does not cover several parts of the world.

Heteroplasmy and microchimerism: I think microchimerism arising from metagenomic assembly should be discussed in

depth, given how it could affect diversity metrics (richness, PD, etc.). Assuming heteroplasmy is occurring (i.e., slightly different mitochondrial genomes in the same cell), during assembly, a consensus or "chimeric" mitogenome could be generated. Something similar could happen with mitogenomes from closely related taxa. This is probably mitigated to some extent by the applied clustering, but I think it should be tested against mitogenomes produced from single cells, single mitochondria, etc. Connected to this, multiple assemblers have been used, even different versions of the same assembler. Given that the performance of some assemblers can differ (e.g., Spades vs. megahit), it should be discussed how this could affect the results.

Size fractions: It is mentioned that most samples originated from the 0.2-5um size fraction. This covers mostly the pico-plankton (0.2-3um), and a small portion of the nano-plankton (3-20um). It does not cover the micro-plankton (20-200um). Thus, the sentence in Line 554, indicating "...facilitating the recovery of mitochondrial genomes from a broad size spectrum of eukaryotic organisms …" does not seem correct, and should be rephrased, indicating that the dataset predominantly represents the signal recovered in the pico-plankton.

Some ecological questions addressed with the produced dataset (e.g., those relating mitogenomes with the trophic state of lakes) seem a bit shallow and too broad, leading to confirmatory results. I wonder whether the manuscript could focus more on the new insights derived from exploiting this large, new, dataset.

Specific comments:

L76: Cyanobacteria also contribute to primary production in the ocean…
L78: "In freshwater AND marine ecosystems…"
Fig1: I'd remove the word "Global" from the legend.
Fig4: What are the differences between the two figures in panel A)?
L406: The information that can be conveyed by phylogenetic overclustering or overdispersion should be explained more here.
L471: Could this diversity be inflated due to microchimerism and heteroplasmy?
L500: I think this paragraph should discuss some points more specifically: microchimerisms, the use of different assemblers and how this can affect results, the distribution of the samples (mostly N.America and Europe), as well as the size fractions from which metagenomes were generated (mostly picoplankton).
L534: There are ca. 16 pages of methods. It'd be good to reduce this section.
L555: Most metageomes represent the picoplankton and are probably enriched in picoeukaryotes. Discuss the signal of nanoeukaryotes and microeukaryotes and how this can be related to the sampling process, etc.
L558: More information should be provided about the parameters used with the different assemblers, naming all those that have been used.

Version 1:

Reviewer comments:

Reviewer #1

(Remarks to the Author)
It has been a while since I reviewed the first version of this manuscript, so I had forgotten some of the details. After reading the revised version, however, I am even more convinced that this is a timely and much-needed resource. Congratulations to the authors!

I am really impressed by how much time, effort, and thought the authors put into addressing my comments. The extent of the additional analyses and in silico tests is impressive. I am also happy to see that these additional analyses did not affect the main conclusions, or only did so marginally, for example by connecting genome fragments into a single assembly.

I think the manuscript has also improved considerably thanks to the more precise wording regarding the recovery of mitochondrial genomes and the clearer presentation of sequence similarity, uniqueness, taxonomy, and completeness. I particularly appreciate that the authors now explicitly describe the resource as an atlas of mitochondrial genome fragments, which I think is a much more accurate way of presenting the dataset.

As I wrote in my original review, I find this work both interesting and highly valuable. The authors have thoroughly addressed my concerns, and I am fully convinced by the additional analyses and the revised manuscript. Congratulations again on an excellent piece of work.

Reviewer #2

(Remarks to the Author)
Authors have addressed all concerns satisfactorily but one. Fantastic work everyone!

Figure 2a SAR should be split into Stramenopiles Rhizaria and Alveolates to help clarify.

I do not need to see the manuscript again and this should be seen as a minor correction and not a revision.

Reviewer #3

(Remarks to the Author)
I co-reviewed this manuscript with one of the reviewers who provided the listed reports. This is part of the Nature Communications initiative to facilitate training in peer review and to provide appropriate recognition for Early Career Researchers who co-review manuscripts.

**Open Access** This Peer Review File is licensed under a Creative Commons Attribution 4.0 International License, which permits use, sharing, adaptation, distribution and reproduction in any medium or format, as long as you give appropriate credit to the original author(s) and the source, provide a link to the Creative Commons license, and indicate if changes were made.
In cases where reviewers are anonymous, credit should be given to 'Anonymous Referee' and the source.
The images or other third party material in this Peer Review File are included in the article’s Creative Commons license, unless indicated otherwise in a credit line to the material. If material is not included in the article’s Creative Commons license and your intended use is not permitted by statutory regulation or exceeds the permitted use, you will need to obtain permission directly from the copyright holder.
To view a copy of this license, visit https://creativecommons.org/licenses/by/4.0/

# Point-to-Point response to Reviewers

## Manuscript title: "***A Global Mitochondrial Genomic Atlas Illuminates Freshwater Microeukaryote Diversity***" (NCOMMS-26-001174-T)

Dear reviewers,

We are sincerely grateful for the time, care, and expertise you devoted to evaluating our manuscript. Your detailed and constructive comments have been highly valuable in helping us revisit our analyses, clarify our presentation, and refine the framing of our main conclusions. We acknowledge that several of the concerns raised reflected limitations in the way certain data, methodological choices, and caveats were presented in the original version. In response, we have reorganised key figures, expanded the methodological descriptions, added further quantitative summaries where appropriate, and revised the discussion of methodological limitations to make the scope and interpretation of our findings clearer. We believe that these changes have substantially strengthened the manuscript and have addressed the reviewers' comments in a transparent and comprehensive manner.

Below, we respond to each comment in turn, indicating how the feedback has been incorporated and where additional analyses, clarifications, or textual revisions have been made. Reviewers' comments are reproduced in italics, and our responses are provided in regular text, rendered in dark blue. All line numbers refer to the revised manuscript with tracked changes.

## REVIEWER #1

**Comment "*Remarks to the Author*"**: *"The manuscript entitled "A Global Mitochondrial Genomic Atlas Illuminates Freshwater Microeukaryote Diversity" represents a timely and important contribution to our understanding of microbial eukaryotic diversity and ecology in freshwater systems. The authors take advantage of large-scale metagenomic datasets that have traditionally been explored primarily for prokaryotic diversity, instead using organellar (mitochondrial) genomes to access eukaryotic diversity. This is a powerful idea that helps bridge a longstanding gap caused by the difficulty of recovering nuclear eukaryotic genomes from metagenomic data, a limitation that has led to microbial eukaryotes being underexplored in many datasets.*

*While the general concept of leveraging mitochondrial genomes from metagenomes has been developing over the past several years, both conceptually and methodologically, this study substantially extends previous efforts by applying the approach at an unprecedented scale and with a specific focus on freshwater lakes. The resulting atlas convincingly demonstrates the potential of mitogenomic data to reveal previously uncharacterized diversity and ecological patterns, and it clearly advances our understanding of freshwater microeukaryotes.*

*Overall, we find this manuscript to be very interesting and well written, with clear potential for significant impact. The work represents a substantial technical and conceptual advance, and the dataset assembled here will likely be a valuable resource for the community. That said, there are several points where additional clarification, discussion, or quantitative support would further strengthen the manuscript and help readers better interpret the scope and limitations of the approach."*

**Response**: We thank Reviewer #1 for their careful reading of our manuscript and for their encouraging assessment of the study. We also appreciate the reviewer's constructive comments, which helped us clarify the scope of the atlas, strengthen the quantitative support for our conclusions, and discuss the methodological limitations of the approach more explicitly. We have revised the manuscript accordingly and respond to each comment in detail below.

**Major Comments**

**Comment 1**: "*While many recovered mitochondrial genomes can be confidently placed within higher-level clades, the lack of comprehensive mitochondrial reference databases clearly limits taxonomic assignment at lower ranks. This important limitation would benefit from a more explicit discussion. For example, how many of the recovered mitochondrial genomes could be assigned a species name? Quantifying this would help readers better assess the current limits of taxonomic resolution imposed by reference database gaps."*

**Response 1:** We thank the reviewer for this comment. We agree that the limits of lower-rank taxonomic assignment needed to be quantified more explicitly.

We have now added this information to the revised manuscript. Using high-identity matches to mitochondrial marker databases for 12S rRNA, 16S rRNA, and COI, and applying a conservative ≥98% identity threshold, 846 of the 19,925 recovered mitochondrial genome fragments could be assigned to genus level, corresponding to approximately 4.2% of the atlas. We report this value at genus level because it is the lowest rank that can be applied consistently across the marker resources used here; 12S rRNA and 16S rRNA references are not uniformly species-resolved, whereas COI-based species names would apply only to a subset of recovered fragments. We have revised the text to clarify that.

<u>This result is now stated in the Results section (Lines 403-426), and the corresponding lower-rank taxonomic assignments have been added to Supplementary Data S6</u>.

Lines 403-426: "*At the same time, lower-rank taxonomic resolution remained limited. High-identity searches of recovered 12S rRNA, 16S rRNA, and COI genes against mitochondrial marker databases linked 855 of 19,925 mitochondrial genome fragments to named genera at ≥98% identity, corresponding to 4.3% of the atlas.* Notably, 748 FMA clusters could not be confidently assigned to any taxonomic rank. *Together, these patterns show that the*

*FMA expands coverage most strongly in portions of microeukaryotic diversity that remain poorly represented in current mitochondrial reference databases.*"

**Comment 2:** "*Relative abundances are central to the ecological analyses in this study. The authors propose an innovative approach based on mitochondrial genomes, using cluster size as a proxy for relative abundance. While this is an interesting and promising idea, some additional clarification and discussion of potential biases (e.g., effects of mitochondrial copy number, genome fragmentation, or assembly success) would be helpful, as this approach is relatively new.*"

**Response 2:** We agree with the reviewer that the interpretation of cluster size required clearer explanation, especially because mitochondrial genomes vary in copy number across eukaryotic lineages.

**A**. We have revised the manuscript to clarify that cluster size is not intended to represent cell abundance, biomass, or mitochondrial copy number. Rather, it is used as an incidence-like measure of repeated mitochondrial recovery. Species-level mitochondrial units were defined using the empirically calibrated 98.1% identity threshold, and cluster size records how often mitochondrial genome fragments assigned to a given species-level unit were recovered within an ecological unit (e.g., lake trophic category, season and strata). Thus, the framework follows the logic of biodiversity surveys based on repeated detections of taxa across samples, rather than read-depth-based abundance estimation. This design was used specifically to reduce the direct influence of mitochondrial copy-number variation.

We added the following clarification in the Methods section (Lines 1487-1492): "*Cluster size was used as a recovery-based representation of each species-level mitochondrial unit within a trophic category. We use this metric as an incidence-like measure of repeated mitochondrial recovery, rather than as a direct estimate of organismal abundance, biomass or mitochondrial copy number. Incidence data were compiled into a trophic group-by-species matrix and reformatted into a list suitable for rarefaction analysis*."

**B**. To further evaluate the possible effects of sequencing technology and assembler choice on mitochondrial sequence identity, we performed a proof-of-concept analysis using a ZymoBIOMICS mock community containing *Saccharomyces cerevisiae* as the eukaryotic component within a bacteria-dominated community (approx. 96% of total DNA pool of bacterial origin). The same mock community was sequenced using both Illumina short reads and Oxford Nanopore long reads, and assemblies were generated independently with MEGAHIT and metaSPAdes for Illumina data, and metaFlye and Canu for Nanopore data. We then recovered the *S. cerevisiae* mitochondrial genome/genome fragments from each assembly and compared them pairwise using ANI.

The recovered mitochondrial sequences were highly concordant across sequencing platforms and assemblers. Pairwise identities ranged from 99.94% to 100%, with a median identity of 99.97%. These values are well above the 98.1% identity threshold used for species-level clustering in the atlas, indicating that, when the same mitochondrial unit is recovered, differences in sequencing technology or assembler choice do not move it

across the species-level boundary used in our analyses. This interpretation is consistent with mock-community metagenomic benchmarks showing that shotgun sequencing and assembly can recover the expected organisms present in defined communities and that assembled contigs or genomes can match their known reference genomes at very high ANI, often >99.9% (under sufficient coverage provided)[1-4]. We have added this proof-of-concept analysis to the revised Supplementary Information (see Mock-community benchmarking of sequencing and assembly effects).

References


1. Luo, C., Tsementzi, D., Kyrpides, N. C. & Konstantinidis, K. T. Individual genome assembly from complex community short-read metagenomic datasets. The ISME journal 6, 898–901; 10.1038/ismej.2011.147 (2012).
2. Serra Moncadas, L., Hofer, C., Bulzu, P.-A., Pernthaler, J. & Andrei, A.-S. Freshwater genome-reduced bacteria exhibit pervasive episodes of adaptive stasis. Nature communications 15, 3421; 10.1038/s41467-024-47767-7 (2024).
3. Liu, L., Yang, Y., Deng, Y. & Zhang, T. Nanopore long-read-only metagenomics enables complete and high-quality genome reconstruction from mock and complex metagenomes. Microbiome 10, 209; 10.1186/s40168-022-01415-8 (2022).
4. Nicholls, S. M., Quick, J. C., Tang, S. & Loman, N. J. Ultra-deep, long-read nanopore sequencing of mock microbial community standards. GigaScience 8; 10.1093/gigascience/giz043 (2019).


**C**. We also tested whether mitochondrial genome fragmentation could affect phylogenetic placement and Faith's phylogenetic diversity (PD) estimates.

To evaluate this directly, we performed a controlled fragmentation analysis using recovered mitochondrial genome fragments from two taxonomic contexts represented in the FMA. First, we selected two Cryptomonadaceae mitochondrial genome fragments of 20.3 and 22.7 kbp. Second, we selected 54 SAR mitochondrial genome fragments assigned to Ochrophyta, Ciliophora, and Cercozoa, ranging from 12.0 to 71.2 kbp. These sequences were artificially split into 2 to 18 non-overlapping fragments per sequence, with a median of 3 fragments. We then inferred phylogenies using the same markers, software, versions, and parameters as in the manuscript, comparing trees generated from the longer recovered fragments with trees generated from their artificially fragmented counterparts.

The results showed that fragmentation did not alter the phylogenetic placement of the tested lineages. Manual comparison of the trees showed that the fragmented sequences remained placed within the same branch regions as their corresponding longer fragments, and the topology was unchanged with respect to the focal groups.

We then quantified the effect on Faith's PD. In both test cases, the fragmented treatment did not inflate PD relative to the longer-fragment treatment. Instead, PD increased when longer mitochondrial fragments were used: by 1.6% in the Cryptomonadaceae test and by 11% in the SAR test (Supplementary Data S9.2). This indicates that, in these controls, fragmentation led to more conservative PD estimates rather than artificial inflation. A likely explanation is that longer fragments capture a larger fraction of the mitochondrial marker

space and therefore include more lineage-specific divergent sites, particularly for environmental taxa that are distant from available references. Shorter fragments still retained sufficient signal for placement, but captured less of the terminal divergence contributing to PD.

We now explicitly acknowledge in the Limitations section (Lines 827-1029) that mitochondrial genome completeness and fragmentation can affect absolute PD values. Importantly, however, the direction and magnitude of the control analysis indicate that fragmentation is unlikely to explain the large phylogenetic diversity gains reported for the FMA. The observed effects of fragmentation, 1.6% and 11% in the tested groups, are small relative to the PD increases reported for FMA. We therefore treat fragmentation as an important caveat for absolute PD estimation, but not as a likely driver of the broad phylogenetic gains reported in the manuscript.

We have reshaped the Limitations paragraph located after the Discussion chapter (Lines 996-1000; 1016-1021):

"*Fragmentation may also affect absolute phylogenetic diversity estimates: shorter fragments can retain sufficient signal for phylogenetic placement, but may capture fewer lineage-specific divergent sites than longer fragments, making branch-length and PD estimates potentially conservative in some cases.*

*We interpret cluster size and absolute PD values with caution, placing emphasis on standardized comparisons across trophic states, seasons, and depths rather than on the absolute number of recovered fragments. Coverage-based rarefaction, bootstrap resampling, sample-size rarefaction, and null models reduce the influence of uneven recovery effort, but they cannot eliminate lineage-specific differences in mitochondrial architecture or assembly probability*."

**Comment 3:** "*An important question concerns how well the pipeline performs for non-canonical mitochondrial genomes, including fragmented, linear, highly reduced, or otherwise atypical architectures, which are relatively common among microbial eukaryotes. The manuscript touches on this only briefly. It would be valuable to know whether any such atypical mitochondrial genomes were recovered in the dataset, and how they behaved during filtration and clustering. In addition, the literature cited on reduced mitochondrial genomes appears somewhat outdated.*"

**Response 3:** We agree that non-canonical mitochondrial genomes are highly relevant for microbial eukaryotes. However, the study does not reconstruct or classify complete mitochondrial genome topology. The FMA is built from genomes and genomic fragments, not from experimentally validated, architecture-resolved mitochondrial chromosomes. Therefore, we cannot determine for each entry whether the source molecule was circular, linear, fragmented, multipartite, or highly reduced. We now state this explicitly in the revised manuscript. This distinction is important because topology requires assembly continuity and molecule-level evidence, whereas our framework was designed to recover high-confidence mitochondrial genomic assemblies from complex metagenomes.

At the same time, the framework is not built around the canonical single circular metazoan mitochondrial genome. Candidate sequences were selected and curated using contig-level features: sequence composition, predicted mitochondrial genes, rRNA/protein annotation, genetic-code-aware gene prediction, and orthogonal contamination screening. Clustering was performed with query-side coverage, specifically to tolerate fragmented assemblies while limiting spurious clustering from short local overlaps. Thus, linearity or circularity is not a criterion used by the pipeline and should not, by itself, cause exclusion. The relevant limitation is instead whether a mitochondrial sequence is long enough, assembled, and sufficiently represented in reference space to pass the filters.

We have also clarified how the length filter affects this issue. The Methods already state that only contigs between 5 kbp and 122 kbp were retained, corresponding to the 2.5th–97.5th percentiles of mitochondrial genome lengths in RefSeq. This filter was chosen to enrich for bona fide mitochondrial contigs and to provide enough sequence complexity for k-mer-based classification. However, we now emphasize that this design can under-recover very reduced mitochondrial genomes, minicircle-like molecules, or short architecture-specific fragments below the 5 kbp threshold.

We also examined the taxonomic composition of the retained dataset in relation to the reviewer's question. The final atlas retained broad freshwater microeukaryotic diversity, including SAR, Cryptophyceae, Haptista, Stramenopiles, Oomycota, Fungi, Viridiplantae, Discoba, and Gnathifera/rotifer-associated lineages. Most relevant to the question of non-canonical architecture, the retained assignments include many Ciliophora. This matters because ciliates are one of the clearest microbial eukaryote groups in which linear mitochondrial genomes are common. Therefore, the recovery of many ciliate-assigned mitochondrial fragments indicates that the pipeline did not simply collapse toward canonical circular-genome lineages or systematically remove sequences from lineages where linear mitogenomes are expected.

We have deliberately phrased this point conservatively. The presence of ciliate-assigned mitochondrial fragments supports recovery from a lineage with commonly linear mitogenomes; it does not demonstrate that we reconstructed complete linear mitochondrial chromosomes. Similarly, recovery of rotifer-associated, oomycete, fungal, discoban, and other microbial eukaryotic assignments indicates broad retention of divergent mitochondrial signal, but it does not allow topology to be assigned taxon-by-taxon. This is especially important because most FMA entries are distant from current databases: only 4.3% of mitochondrial genome fragments could be linked to named genera at ≥98% identity, and 748 FMA clusters could not be confidently assigned to any taxonomic rank. Under this level of reference divergence, assigning genome topology by taxonomic match alone would be very unreliable.

We therefore now frame atypical mitochondrial architecture as a recoverability and interpretation issue, not as a directly measured feature of the atlas. The revised manuscript states that non-canonical architectures may influence recovery when they produce short fragments, extreme gene loss or highly divergent sequence composition. This is consistent with the caveats already discussed in the manuscript: mitochondrial copy number, genome size, genome structure, fragmentation, and reference representation can

influence read recruitment, assembly, clustering, and phylogenetic placement. We have strengthened this section and updated the literature cited on atypical mitochondrial genomes, and recent work linking mitochondrial genome fragmentation to molecular evolutionary rate. In summary, we do not claim that the FMA reconstructs mitochondrial genome topology. Rather, our data show that the framework retains high-confidence mitochondrial genome fragments from a broad diversity of freshwater microeukaryotes, including lineages in which non-canonical architectures are expected. We now make explicit that topology cannot be inferred directly from these data, and that highly reduced or extremely fragmented architectures remain a likely source of under-recovery.

We have added the following clarification in the Methods sections (Lines 1110-1112): "*However, while the filter improves specificity it may exclude very small mitochondrial genomes, minicircle-like molecules, or short architecture-specific fragments below 5 kbp*."

A new limitation section was added (Lines 828-1029).

We have updated the literature to better cover reductive genome evolution in mitochondria with the following works:

*Boxma, B. et al. An anaerobic mitochondrion that produces hydrogen. Nature 434, 74–79; 10.1038/nature03343 (2005).*
*Karnkowska, A. et al. A Eukaryote without a Mitochondrial Organelle. Current biology : CB 26, 1274–1284; 10.1016/j.cub.2016.03.053 (2016).*
*Graf, J. S. et al. Anaerobic endosymbiont generates energy for ciliate host by denitrification. Nature 591, 445–450; 10.1038/s41586-021-03297-6 (2021).*
*Novák, L. V. F. et al. Genomics of Preaxostyla Flagellates Illuminates the Path Towards the Loss of Mitochondria. PLoS genetics 19, e1011050; 10.1371/journal.pgen.1011050 (2023).*
*Najer, T. et al. Mitochondrial genome fragmentation is correlated with increased rates of molecular evolution. PLoS genetics 20, e1011266; 10.1371/journal.pgen.1011266 (2024).*

**Comment 4:** "*To recover mitoginoomes authors used an approach based on multilayer filtering of the candidate mitoginomes, followed by clustering based on an empirically inferred species-level threshold. This is an interesting and pragmatic solution, but it raises some concerns. In particular, if a single mitochondrial genome is split into two or more non-overlapping fragments, these fragments would not cluster and could be represented as separate mitogenomes in the atlas. Perhaps that is addressed by the search for conserved genes. Some discussion of how frequently this might occur, and how it could affect downstream diversity estimates, would be useful.*"

**Response 4:** We thank the reviewer for this comment. To address this possibility directly, we added a *post hoc* validation step based on metagenomic binning. Because non-overlapping fragments cannot be linked by nucleotide identity alone, we asked whether any FMA species representatives showed evidence of belonging to the same mitochondrial genome using information independent of sequence overlap: tetranucleotide composition and differential coverage across metagenomes (n = 429). This follows the logic of genome-resolved metagenomics, where contigs belonging to the same genome are linked by shared sequence composition and co-variation in coverage across samples, but here we adapted the approach to mitochondrial representatives.

Candidate co-binned fragments were retained only when they also showed compatible LCA taxonomic assignments and non-redundant mitochondrial gene repertoires, ensuring that coverage and composition alone were not sufficient to merge entries.

This analysis identified 63 multi-contig bins involving 114 original FMA representatives that fulfilled these criteria and were therefore interpreted as likely cases of non-overlapping fragments belonging to the same species-level mitochondrial unit. These representatives were collapsed into 63 revised species-level units before downstream species-centric analyses were recalculated. This correction involved 3.1% of the original FMA representatives and reduced the total number of representatives by 51, corresponding to a net reduction of 1.4%. Thus, fragmentation-driven over-splitting was detectable but limited, and the revised FMA no longer carries these cases forward as independent species-level representatives.

We updated the species-centric analyses accordingly. The total number of recovered species-level representatives decreased only marginally, while phylogenetic diversity estimates changed only slightly, increasing marginally after fragment consolidation. This is consistent with our additional fragmentation assessment described in Response 2C: non-overlapping fragments from the same mitochondrial lineage retain the phylogenetic signal of that lineage and are therefore unlikely to generate spurious placement across distant clades, although genome completeness can affect absolute branch-length and PD estimates (Supplementary Data S9.2). Figure 3a and all other species-centric results were updated to reflect the corrected species-level representative set. Figure 3b did not require revision because the COI-based tree uses COI-bearing representatives and was not affected by these inferred fragment consolidations, and Figure 3c was retained because it summarizes functional annotations independently of species counts.

We have added this post hoc binning validation to the Methods section and revised the Discussion to clarify that the final FMA incorporates this correction. We now present fragmentation-driven over-splitting as a minor residual caveat, most relevant to rare or low-coverage lineages that cannot be robustly evaluated by coverage-based binning.

Methods (Lines:1326-1339) : "*To assess whether distinct FMA clusters representatives could correspond to non-overlapping fragments of the same mitochondrial genome, we performed a post hoc binning validation using 429 metagenomes. Multi-sample contig coverage profiles were generated with Fairy v0.5.8 using the sketch and coverage workflows with default settings. FMA species representatives were then binned with MetaBAT2 v2.2.17 using default settings. Multi-fragment bins were considered candidate fragmentation events and were further evaluated using two consistency criteria: contigs within a bin were required to show congruent lowest common ancestor taxonomic assignments and non-redundant mitochondrial gene repertoires. Bins satisfying both criteria were interpreted as cases in which multiple recovered representatives likely corresponded to fragments of the same species-level mitochondrial unit. In total, 63 bins involving 114 FMA representatives fulfilled these criteria. These representatives were collapsed into 63 revised species-level units and used in downstream species-centric analyses.*"

Limitations (Lines: 972-980): "*Potential over-splitting of non-overlapping mitochondrial fragments was minimized in the FMA before downstream analyses through post hoc curation based on tetranucleotide composition, differential coverage, taxonomic consistency, and non-redundant mitochondrial gene content. This procedure captures the most detectable cases of fragmentation-driven over-splitting, although residual cases may remain for rare or low-coverage lineages that cannot be robustly evaluated by coverage-based binning. Such cases could lead to a slight overestimation of species counts, but are unlikely to drive the broad diversity patterns reported here.*"

**Comment 5:** "*Following that comment, we think that more systematic statistics describing the recovered mitogenomes, such as size distributions, GC content, and gene content across major groups, ideally in comparison to their closest known relatives is missing. While some of this information is available in supplementary materials, bringing a clearer summary into the main text would strengthen the manuscript. Such metrics could also serve as proxies for genome completeness, which is particularly important given that many recovered mitogenomes appear to be partial. Clarifying how complete a mitochondrial genome needs to be to support species-level or ecological conclusions would help contextualise the results.*"

**Response 5:** We agree that the manuscript needed a clearer and more systematic summary of the recovered mitochondrial genome fragments, particularly because many records are partial and genome completeness affects how the atlas should be interpreted.

We have now added a genome-property summary to the main Results section and expanded the supplementary material with Suppl. Table S1.

We added and reshaped the following part of the Results section (Lines: 374-399): "*Across the 19,925 curated mitoMAGs, recovery spanned the full trophic gradient: 9,468 records (47.5%) derived from eutrophic and meso-eutrophic systems, 4,415 (22.2%) from oligo-mesotrophic and mesotrophic systems, 5,420 (27.2%) from oligotrophic systems, and 622 (3.1%) from lakes lacking a formal trophic assignment (Fig. 1). Collectively, these mitoMAGs represent 3,662 species-level clusters and are typically compact, AT-rich, and gene-dense, with a median length of 9.1 kb, median GC content of 28.9%, and median coding density of 69.9%. The catalogue contains 265,367 predicted genes, including 4,561 12S rRNA, 4,672 16S rRNA, and 6,991 COI loci. Importantly, these length statistics describe recovered mitochondrial genome-associated reconstructions rather than necessarily complete mitochondrial genomes. At the species-representative level, mitoMAGs ranged from 5.0 to 100.95 kbp, with a median of 15.09 kbp and a mean of 19.15 kbp.*

*Genome properties differed among major groups and, where comparable RefSeq r226 records were available, provided a reference context for interpreting contiguity and gene recovery. For example, arthropod representatives (n = 111) were close to RefSeq r226 arthropod mitogenomes (n = 5,956) in median length and protein-coding gene number (15.08 vs 15.59 kbp; 11 vs 12 protein-coding genes), whereas ciliophore representatives (n = 571) were shorter and contained fewer predicted protein-coding genes than available RefSeq r226 ciliophore mitogenomes (n = 20; 16.28 vs 47.14 kbp; 15 vs 41 protein-coding genes). This contrast indicates that some FMA representatives correspond to compact,*

*near-reference-length mitochondrial genomes, whereas others capture shorter gene-bearing portions of larger or structurally complex mitochondrial genomes. Full group-level summaries of size, GC content, coding density, gene content, and RefSeq comparisons are provided in Supplementary Table S1."*

We have also clarified how completeness relates to downstream inference. Because mitochondrial genome size and architecture vary substantially across eukaryotes, we do not define completeness using a single universal length or gene-count threshold. Instead, completeness requirements depend on the analysis. Species-level clustering requires sufficient overlapping mitochondrial sequence to satisfy the 98.1% identity and coverage criteria, together with mitochondrial gene evidence and contamination filtering. Phylogenetic analyses require the presence of relevant marker genes, whereas ecological comparisons rely on standardized recovery of species-level mitochondrial units across trophic states, seasons, or depths. We now state explicitly that the FMA should be interpreted as a curated atlas of mitochondrial genome fragments and species-level mitochondrial units.

The following paragraph was added in the Limitations section (Lines: 986-1000):
*"Completeness is best interpreted in relation to the inference being drawn. Complete mitogenome assemblies remain necessary for resolving genome architecture, gene order, and full organellar gene repertoires. The FMA analyses, however, were designed around curated mitochondrial signal rather than complete mitochondrial genomes in every case. Species-level units were defined from fragments with sufficient mitochondrial overlap to satisfy the 98.1% identity and coverage criteria, and were retained only after mitochondrial gene-content validation, contamination filtering, and post hoc checks for fragmentation-driven over-splitting. Phylogenetic analyses used representatives carrying the relevant marker genes, whereas ecological analyses relied on standardized recovery of curated species-level units across trophic states, seasons, and depths. Under this framework, partial but well-curated mitochondrial fragments can support species-level clustering, phylogenetic placement, and comparative ecological inference, while complete assemblies remain essential for questions concerning mitochondrial architecture, genome organization, and full gene-content evolution."*

**Comment 6:** *"The species delineation strategy is innovative and potentially very powerful. However, the calibration dataset used to infer species-level thresholds is described as being biased toward multicellular eukaryotes. Providing more quantitative information on the taxonomic composition of this dataset (e.g., distribution across major eukaryotic supergroups) would help readers better evaluate how transferable the threshold is to various groups of microbial eukaryotes."*

**Response 6:** We agree with the reviewer that the taxonomic structure of the calibration dataset needs to be documented more explicitly. The taxa used for threshold analysis inference are provided in the Source Data for Fig. 2a,b, and we have now added a supplementary figure (Suppl. Fig. S5) summarizing their distribution across major eukaryotic groups.

The composition of the calibration dataset was necessarily constrained by the current structure of mitochondrial reference databases. To infer the threshold, we required genera represented by at least seven named species and species represented by at least seven conspecific mitochondrial genomes, so that within-species and between-species identity distributions could be estimated confidently. These criteria were essential for a robust calibration, but they also enriched the dataset for lineages that are better represented in RefSeq r226, particularly multicellular eukaryotes.

Importantly, we tested whether this imbalance affected the inferred identity distributions. Mitochondrial genome sequence identity did not differ significantly between macro- and microeukaryotes, either within species or between species (Wilcoxon rank-sum test with Bonferroni correction; within species, $p = 0.07$; between species, $p = 0.65$). This suggests that, within the reference data currently available, organismal complexity does not introduce a systematic shift in the mitochondrial identity distributions used to derive the threshold.

To further evaluate transferability to unicellular eukaryotes, we performed an additional targeted evaluation of the threshold using a dataset enriched in unicellular taxa. Briefly, we curated the RefSeq r226 mitochondrial database (n = 17,771 mitogenomes) by excluding multicellular eukaryotes through taxonomy-informed filtering. This procedure yielded a reduced dataset comprising 482 unicellular mitochondrial genomes. We then re-applied our species delineation threshold (i.e., 98.1%) to this filtered dataset. Clustering resulted in 472 clusters, of which 463 were singletons and only 9 comprised multiple genomes. This corresponds to an estimated accuracy 97.9% and a recall 96.1%; exceeding the performance obtained on the full RefSeq dataset (~92.4% concordance with current taxonomy). This result shows that the threshold retains its discriminatory power when restricted to unicellular eukaryotes.

In addition to Suppl. Fig. S5, we added the following in Results section (Lines: 264-268): "*To evaluate transferability to unicellular eukaryotes, we further applied the threshold to a unicellular-enriched RefSeq r226 subset (482 species) generated by taxonomy-informed removal of multicellular lineages. In this subset, 97.9% of mitogenomes remained separated as species-level clusters, supporting the threshold for the unicellular diversity currently represented in mitochondrial reference databases.*"

We further updated the Fig. 2a,b.

**Comment 7:** "*The phylogenetic tree is presented at a relatively general level. Several large clades appear to be dominated by newly recovered mitochondrial genomes, including within Metazoa. Commenting in more detail on what kinds of taxa or lineages this "dark diversity" may represent would be useful.*"

**Response 7:** We revised Fig. 3b to show the phylogeny at finer taxonomic resolution, so that the main lineages contributing to the newly recovered mitochondrial diversity are more apparent. We also clarified in the figure legend that RefSeq r226 Metazoa were subsampled for visualization, because metazoan mitogenomes dominate the reference database.

The metazoan signal in the FMA is biologically expected in freshwater environmental metagenomes. The recovered lineages include aquatic taxa or aquatic life stages whose biomass, eggs, larvae, tissue fragments, or extracellular DNA can be retained during sampling. These include rotifers such as Brachionus, Polyarthra, Synchaeta, Ascomorpha, and Pompholyx; copepods and crustacean zooplankton such as Acanthocyclops, Eurytemora, Neocalanus, Daphnia, and Diaphanosoma; freshwater bivalves such as Dreissena; chironomids such as Chironomus; and water mites such as Unionicolides. Several of these taxa have microscopic body sizes or propagules, including rotifers of approximately 50–500 µm, bivalve eggs of approximately 40–100 µm, copepod eggs of approximately 75–350 µm, Daphnia and Diaphanosoma eggs of approximately 150–450 µm, and chironomid eggs of approximately 150–250 µm. We therefore retained these records as part of the freshwater mitochondrial signal, while improving the figure annotation so their placement and taxonomic context are clearer. The full diversity record is found in Supplementary Data S6.

<u>We added the following paragraph to the Discussion section (Lines: 749-791)</u>: "*The newly recovered mitochondrial diversity is distributed across multiple freshwater-relevant eukaryotic lineages rather than concentrated in a single unresolved branch. It spans a broad biological range, from metazoan-affiliated mitochondrial representatives assigned to molluscs, rotifers and microcrustaceans, to unicellular planktonic groups such as ochrophytes, ciliates, rhizarians, cryptophytes, haptists, fungi and heterotrophic flagellates. These lineages include phytoplankton, bacterivorous and algivorous protists, parasitic or saprotrophic fungi, rotifers, copepods and cladocerans, indicating that the FMA expands mitochondrial representation across several trophic compartments of freshwater ecosystems. Their divergence from named references likely reflects limited mitochondrial sampling of freshwater microeukaryotes, scarce cultivated or single-cell genomic representation for many planktonic taxa, and the rapid sequence and structural evolution of eukaryotic mitochondrial genomes. Thus, the FMA broadens mitochondrial coverage across undersampled regions of the freshwater eukaryotic tree, rather than simply increasing representation within already well-characterized clades.*"

## Minor Comments

**Comment 8:** "*Lines 294–303 focus on Geminigeraceae mitochondrial genomes. While this is interesting, discussing a single, specific group in such detail is in contrast with the otherwise large-scale perspective of the study. A clearer motivation for highlighting this particular case, or a brief indication of its broader relevance, would help it fit more naturally into the manuscript.*"

**Response 8:** We agree that the motivation for highlighting the Geminigeraceae locus needed to be clearer.

We have revised this section to make explicit that the example is not intended as a taxon-specific digression, but as an illustration of a broader point: the FMA expands not only mitochondrial taxonomic and phylogenetic coverage, but also the recoverable mitochondrial gene-content space. The atlas shows that freshwater microeukaryotes are

not only divergent in sequence from current references; in some cases, they also encode mitochondrial loci with architectures not represented in available databases.

Geminigeraceae provided a suitable example because it is well represented in the FMA, with 1,079 recovered mitochondrial fragments and 96 species-level representatives, and because the locus was recurrent, being detected in 14 curated mitogenomes. The locus encodes a type I restriction–modification DNA methyltransferase (HsdM) fused to an HsdS target-recognition domain and lies next to a transposase-like ORF. This architecture is characteristic of bacterial restriction–modification/mobile-element systems and is consistent with a bacterial-derived acquisition into a mitochondrial genome context. We have therefore retained the example, but reframed it to make its broader relevance clear: it demonstrates how environmental mitogenome recovery can expose candidate gene-content innovations that are absent from current mitochondrial reference space.

We have modified the Results section (Lines: 502-511) to: "Among the rare FMA-unique signals, we identified a recurrent candidate non-canonical mitochondrial locus in Geminigeraceae, a cryptophyte family represented in the atlas by 1,079 mitochondrial fragments and 96 species-level representatives. The locus, present in 14 curated mitogenomes, encoded a type I restriction–modification methyltransferase domain fused to an HsdS target-recognition domain, potentially coupling DNA methylation with sequence-specific recognition, and was adjacent to a transposase-like ORF. Its absence from current mitochondrial references and mobile-element-like architecture make it a representative example of how the FMA can reveal rare mitochondrial gene-content novelties, beyond expanding taxonomic and phylogenetic coverage."

We have removed the paragraph in the Discussion concerning Geminigeraceae.

**Comment 9:** "*Line 420 Metazoan mitogenomics per se is well established, but environmental metazoan mitogenomics remains an actively developing field. Providing a bit more context and citing more recent studies would help place the present work in a broader perspective, particularly given that this study also encompasses some metazoan diversity.*"

**Response 9:** We have revised the Discussion to place the metazoan component of the study in a more precise methodological context. The revised text now distinguishes established metazoan mitogenomics from the more recent environmental application of mitochondrial metagenomics, where mitochondrial contigs or genomes are recovered from mixed DNA rather than from individual specimens.

We now state that most previous studies have recovered metazoan mitochondrial data from shotgun sequencing of bulk organismal assemblages, taxon-enriched mixtures, or specimen-associated material. We contrast this with the FMA, where metazoan representatives were recovered non-targeted from freshwater community DNA, consistent with contributions from small aquatic animals, planktonic life stages, eggs, tissue fragments, or extracellular DNA retained during sampling.

We have revised the Discussion section (Lines: 687-700): *"Mitochondrial metagenomics has a strong foundation in metazoan biodiversity research, but its environmental application remains comparatively recent. Most studies have recovered mitochondrial reads or genomes from shotgun sequencing of bulk organismal assemblages, taxon-enriched mixtures or specimen-associated material, rather than from untargeted environmental metagenomes. The metazoan representatives recovered here therefore fall within this emerging field, but arise as non-target recoveries from freshwater community DNA, consistent with contributions from small aquatic animals, planktonic life stages, eggs, tissue fragments or extracellular DNA retained during sampling. For microeukaryotes, by contrast, mitochondrial recovery has largely advanced through single-cell genomics, which has demonstrated feasibility but not broad ecosystem coverage. The present study connects these trajectories by applying de novo mitochondrial recovery to total freshwater community DNA, with microeukaryotic diversity as the primary focus."*

**Comment 10:** *"Supplementary Table S6: Units are missing for the size and GC content columns."*

**Response 10:** We thank the reviewer for pointing this out. We have corrected Supplementary Table S6 by adding the units to the relevant column headers.

**Comment 11:** *"In summary, this is a very strong and exciting manuscript that convincingly demonstrates the power of mitochondrial genomes for exploring (freshwater) microeukaryotic diversity across scales. Addressing the points above, particularly those related to genome completeness, taxonomic resolution, and methodological limitations, would further strengthen the paper."*

**Response 11:** We appreciate the reviewer's positive assessment of the manuscript and the constructive suggestions provided throughout the review. We have addressed the points raised on genome completeness, taxonomic resolution, and methodological limitations by adding new analyses, expanding the relevant methodological descriptions, and revising the discussion of the approach and its caveats. We believe these revisions make the scope and interpretation of the study clearer and strengthen the manuscript.

## REVIEWER 2

**Comment "Remarks to the Author":** *"My name is Jeremy Wideman, I am an Associate Professor in the Center for Mechanisms of Evolution at Arizona State University. I have published several papers on mtDNAs on protists and though I am not an ecologist, I believe I am capable of providing feedback on this manuscript.*
*I am happy for the authors to contact me if they have any questions about this review.*
*The manuscript by Moncadas et al. presents a global atlas of*
*freshwater mitochondrial genomes. The scope of the paper is very large, and a lot of data has been analyzed. I think that there is some excellent work done here, but given the*

*presentation, it is difficult for me to assess what kinds of genomes have been identified and if the Atlas is relatively complete or is extremely biased towards only the most common freshwater organisms (algae and perhaps certain parasites like oomycetes).*
*I have made several suggestions that will help me to understand the completeness of the data and whether it is truly an atlas, or more of a compendium of the most common species/lineages. Even if the Atlas is lacking, the work is still important, however, to maximize the utility of the dataset for other scientists, more information and better clarity on the taxa actually recovered is necessary.*

*I cannot comment on ecology apart from, yes! mtDNAs are useful for ecology!*
*Thus, my comments are largely limited to Figure 3 and the associated supplementary data."*

**Response:** We thank Professor Wideman for his careful assessment of the manuscript and for bringing specialist expertise in mitochondrial evolutionary genomics to the review. We appreciate his positive view of the scope and potential value of the work, and we agree that the original presentation needed to provide clearer information on the nature, completeness, and taxonomic identity of the recovered mitochondrial genomic data. We address each point in detail below.

**Major Comments**

**Comment 1:** *"The authors use the term "mitochondrial genome" where they mean "mitochondrial contig". The mean length of ~9kb for a protist mtDNA is misleading. This is the mean contig length that you have ascribed to being part of mtDNA.*
*How many complete, circular mapping, mtDNAs do you recover?* The authors need to make clearer what it is they are reporting. State the # of mitochondrial genome contigs, the number of unique novel circular mapping mtDNAs."

**Response 1:**

We agree that our previous use of the term "mitochondrial genome (mitogenome)" was too broad and could imply that all recovered records represented complete mitochondrial genomes. This was not our intention. We have therefore revised the manuscript throughout to distinguish more clearly between mitochondrial genomes, and mitochondrial metagenome-assembled genomes/reconstructions (as present in FMA).

<u>We now refer to the recovered metagenome-derived mitochondrial reconstructions as mitochondrial metagenome-assembled genomes (mitoMAGs)</u>. We use this term operationally, analogous to bacterial and archaeal MAGs, to indicate that the records vary in completeness and may include both complete and partial mitochondrial genome reconstructions. We have also revised the Abstract, Introduction, Results, figure labels, and table captions to avoid implying that all records are complete mitochondrial genomes.

<u>In response to the reviewer's specific point, we now make clear that the mean length reported for the full catalogue refers to the mean length of recovered mitoMAGs, not the expected mean length of complete protist mitochondrial genomes</u>. Across the curated

catalogue, we recovered 19,925 mitoMAGs, which were consolidated into 3,662 species-level representative clusters. The full mitoMAG set had a median length of 9.1 kb, whereas the species-level representatives ranged from 5.0 to 100.95 kb, with a median of 15.09 kb and a mean of 19.15 kb. We have added this distinction to the Results section.

We also now report circularized records separately from the broader mitoMAG catalogue. Of the 19,925 curated mitoMAGs, 412 showed terminal overlap consistent with circularization, defined as exact overlapping contig ends of at least 50 bp. These are now described as putatively circularized mitoMAGs, rather than as complete mitochondrial genomes. Among these, 187 correspond to species-level representatives and likely are unique novel circularized mitochondrial DNA records. These values have been added to the Results section and Supplementary Table S6.2.

To further address the reviewer's concern, we added comparative context against RefSeq r226 where available. This comparison shows that some FMA representatives are close to expected reference mitochondrial genome sizes, whereas others are shorter and likely represent partial gene-bearing portions of larger mitochondrial genomes. For example, arthropod representatives were similar to RefSeq r226 arthropod mitogenomes in median length and protein-coding gene number, whereas ciliophore representatives were shorter and contained fewer predicted protein-coding genes than available RefSeq r226 ciliophore mitogenomes. We now explicitly state that this pattern indicates variable completeness across taxonomic groups.

In response to the reviewer's comment, we have now reshaped the first part of the section "A freshwater mitochondrial atlas broadens taxonomic and phylogenetic coverage".

We also note that circularity is not expected to be universal across the taxonomic breadth of freshwater microbial eukaryotes represented in this catalogue. Some groups, including ciliates and other protists, may have linear or otherwise non-canonical mitochondrial genome architectures. For this reason, we now distinguish records with terminal overlap as a putatively circularized subset, but we do not use absence of circularity alone as an exclusion criterion for completeness.

**Comment 2:** *"The authors state that they increase the number of SAR clade mtDNA representatives. SAR is a massive group. This is like saying that you recovered X# of either plant, animal, or fungal mtDNAs without specifying how many of each.*
*To address this issue, trees need to have more taxa identified from every group. At the moment, I cannot assess how much diversity has actually been recovered.*
*There are MANY things that can be done to improve the readability of the trees so that readers can assess the true impact of this work.*
*ALSO: for any further review of this paper I require access to the data including a tree with readable names.*
*At MINIMUM please make the following inferable from the tree (and split figure 3a accordingly as well):"*

**Response 2:** In response to the reviewer's comment we revised Fig. 3b, the unrooted COI-based phylogeny, by adding finer taxonomic labels across the major clades

represented in the FMA. In particular, the revised tree now resolves the composition of broad groups such as SAR at a more informative level, allowing placements within ochrophyte, ciliate and rhizarian lineages to be distinguished rather than treated as a single undifferentiated category.

In addition, because Fig. 3b was designed primarily to provide a marker-gene-based taxonomic anchor for Fig. 3a, we added a complementary rooted mitochondrial phylogenomic reconstruction as Fig. 4. This new analysis provides a broader view of the placement of FMA representatives across eukaryotic mitochondrial diversity and helps readers assess the phylogenetic spread of the recovered lineages beyond the COI-only phylogenetic space.

We have also made the underlying tree file available with readable tip labels as manuscript-associated file and in the Zenodo repository.

**Comment 2A**: "*Stramenopiles: where are the ochrophytes vs oomycetes vs the rest of the stramenopiles? If you ONLY recovered photosynthetic stramenopiles, then the dataset isn't as interesting.*"

**Response 2A:** We agree that Stramenopiles should not be presented as a single undifferentiated category. We therefore increased the taxonomic resolution of the Stramenopiles labels in Fig. 3b.

To address the reviewer's specific concern, we also summarized the FMA taxonomy matches to Stramenopiles affiliated with the COI gene tree. These assignments show that the recovered diversity is not restricted to photosynthetic lineages. Ochrophyta, which include many photosynthetic freshwater planktonic taxa, accounted for 696 species-level representatives and 3,643 mitoMAGs. Their recovery is expected given the abundance and ecological importance of ochrophyte algae in freshwater systems. However, we also recovered a substantial non-photosynthetic stramenopile component, including Oomycota, with 25 species-level representatives and 40 mitoMAGs, and Bigyra, with 2 representatives and 3 mitoMAGs. These groups include osmotrophic, parasitic, saprotrophic and heterotrophic lineages. The presented numbers do not reflect the whole extent of FMA but rather the species representatives from which we could recover COI genes.

Thus, the FMA captures both the expected photosynthetic stramenopile component of freshwater plankton and a non-photosynthetic stramenopile component. The revised Fig. 3b now makes this taxonomic structure explicit. In addition, the newly added Fig. 4 further shows that some representatives that remained "unclassified" after annotation-based taxonomy are phylogenetically placed near Bigyra-like stramenopile lineages, indicating that part of the unresolved FMA fraction may also correspond to undersampled non-photosynthetic stramenopile diversity.

**Comment 2B:** "*Alveolates: Distinguish between dinoflagellates, apicomplexans,ciliates. This helps readers understand what kind of diversity you actually obtained for this group.*"

**Response 2B**: We agree that Alveolata should not be presented as an undifferentiated group, because ciliates, dinoflagellates, apicomplexans and other alveolate lineages represent distinct biological and ecological components. We have therefore revised Fig. 3b to distinguish the main alveolate lineages recovered by the FMA.

The updated annotation shows that the alveolate signal in the COI-based tree is dominated by Ciliophora, with 236 species-level representatives and 1,675 mitoMAGs. We did not recover a substantial dinoflagellate or apicomplexan component in this COI-containing subset, and we now make this explicit rather than implying broad alveolate representation. We also note that these numbers refer specifically to the subset of FMA species representatives for which COI genes were recovered and included in the COI phylogeny, and therefore should not be interpreted as a complete census of all alveolate mitochondrial diversity in the atlas.

The newly added rooted mitochondrial phylogenomic tree in Fig. 4 provides additional context for the alveolate component, placing FMA representatives within Ciliophora and Colponemida-related lineages. Together, these revisions clarify that the alveolate diversity visualized in the FMA is primarily ciliate-associated, with a smaller Colponemida-related component, rather than being driven by dinoflagellate or apicomplexan mitochondrial recoveries.

**Comment 2C:** "*Rhizaria: Unclear what you have included or not in the dataset.*"

**Response 2C:** In the revised Fig. 3b, we now explicitly label the Rhizaria-affiliated representatives recovered in the COI-based tree. In this subset, Rhizaria are represented by 3 species-level representatives corresponding to 5 mitoMAGs. We retained the assignment at the Rhizaria level because the available marker and annotation signal did not support a confident lower-rank subdivision without overinterpretation.

We also clarify that these numbers refer specifically to the COI-containing FMA representatives included in Fig. 3b, and therefore should not be read as a complete census of all Rhizaria-related mitochondrial diversity in the atlas. The newly added rooted mitochondrial phylogenomic tree in Fig. 4 provides additional context by placing several representatives that remained unclassified after annotation-based taxonomy near Rhizaria/Cercozoa-like branches. Thus, the revised figures distinguish the conservatively classified Rhizaria component from additional unclassified FMA lineages with Rhizaria-like phylogenetic affinities. We believe this now makes clear both what is confidently assigned to Rhizaria and where the broader, still under-resolved Rhizaria-related signal occurs in the FMA.

**Comment 2D:** "*Animals and Fungi: what groups have been recovered? Label major phyla*"

**Response 2D:** We have revised Fig. 3b and the corresponding Source Data to indicate the major metazoan and fungal groups recovered in the FMA. For Metazoa, the recovered representatives include Arthropoda (97 species-level representatives; 1,375 mitoMAGs), Lophotrochozoa (5 representatives; 16 mitoMAGs), and Gnathifera (89 representatives;

554 mitoMAGs). These assignments show that the metazoan component is dominated by freshwater-relevant arthropod and rotifer lineages, with smaller contributions from other animal phyla.

For Fungi, the recovered representatives include Ascomycota (10 representatives; 27 mitoMAGs) and Basidiomycota (1 representatives; 1 mitoMAGs). These labels are now shown more clearly in the revised figure/source data, allowing readers to assess which animal and fungal lineages contribute to the recovered mitochondrial diversity.

**Comment 2E:** *"Sometimes there is a yellow line amidst a bunch of blue, these seeming orphans need to be named. If the COIs/16S branches are well supported, then these "unknown groups" become known."*

**Response 2E:** We thank the reviewer for this suggestion. Taxonomy was assigned wherever the marker and LCA evidence supported it, but not all individual labels can be visually represented in Fig. 3b without making the tree unreadable. We have therefore improved the labels for the major informative groups in the figure, while providing the complete per-record taxonomic assignments in Supplementary Data S6.1–S6.3, including the 12S rRNA-, 16S rRNA-, COI- and LCA-based classifications.

In addition, we added a rooted mitochondrial phylogenomic tree as Fig. 4, specifically to bring the previously "unclassified" component of the FMA into clearer evolutionary context. This analysis shows where unclassified mitoMAGs fall relative to reference lineages, while avoiding unsupported lower-rank taxonomic naming when the available references remain sparse.

**Comment 2F:** *"Cryptists and haptists include several lineages of heterotrophs. It SEEMS like this paper has mostly recovered phototrophs. BUT this is unclear from the way the data are presented. Please articulate in the trees where phototrophic vs heterotrophic lineages are."*

**Response 2F**: We agree that broad labels such as Cryptista and Haptista can obscure whether the recovered lineages are mainly phototrophic, mixotrophic, or heterotrophic. We have therefore revised the labels in Fig. 3b to show lower-rank placements where these were supported, and we provide the full taxonomic assignments in Supplementary Data S6.1–S6.3.

For Cryptista, the classified FMA signal includes plastid-bearing cryptophyte lineages, mainly Cryptomonadaceae, Geminigeraceae, Chroomonadaceae and Pyrenomonadaceae, which are best interpreted as predominantly phototrophic or mixotrophic. These accounted for 107 species-level representatives and 2,358 mitoMAGs. We also recovered a heterotrophic cryptist component assigned to Katablepharidaceae, comprising 9 species-level representatives and 281 mitoMAGs. Thus, the cryptist fraction is not exclusively phototrophic, although plastid-bearing cryptophytes make up most of the classified signal.

For Haptista, the classified representatives were assigned to Haptophyta, mainly Prymnesiophyceae and Pavlovales, comprising 25 species-level representatives and 455 mitoMAGs. These lineages are predominantly phototrophic or mixotrophic, and we did not recover a confidently resolved heterotrophic haptist component in the classified FMA subset. We now make this clearer in the tree labels and associated Source Data, distinguishing the dominant phototrophic/mixotrophic signal from the smaller heterotrophic cryptist component where supported.

**Comment 2G:** *"What discobids are in the tree?"*

**Response 2G:** We have now made the Discoba component explicit in the revised Fig. 3b and in Supplementary Data S6.1–S6.3. Based on the LCA taxonomy, Discoba are represented by 3 species-level representatives and 13 mitoMAGs: one Jakobida/Histionina representative comprising 2 mitoMAGs, and two Heterolobosea representatives comprising 11 mitoMAGs.

The COI/eKOI-anchored assignments provide compatible but low-identity placements. The closest matches include the jakobid Histiona aroidea (80.87% identity) and heterolobosean taxa affiliated with Acrasidae/Acrasis kona (70.77%) and Gruberellidae/Stachyamoeba lipophora (72.60–73.16%). Because these identities are low, we do not treat them as species-level identifications. Instead, we report them conservatively as Discoba-affiliated placements. The revised Fig. 3b and the new rooted mitochondrial phylogenomic tree in Fig. 4 now make these Jakobida- and Heterolobosea-related components easier to inspect.

**Comment 2H**. *"The COI proteins for any putative "unknown lineage" should be used as BLAST queries into a mitochondrial database that includes ALL published mitochondrial genomes. This might be difficult, but it is important to know if you have found a new lineage or if the database you are basing your inferences off of is severely incomplete."*

**Response 2H:** To address the reviewer's specific point, we additionally used the COI sequences available from LCA-unclassified representatives as queries against the NCBI nr database. The closest NCBI matches, their identities, and associated taxonomies are now reported in Supplementary Data S6.3. These searches show that some unclassified representatives do have detectable affinities to published mitochondrial sequences, but often at low identity and/or with inconsistent lower-rank placement. We therefore did not convert these cases into formal taxonomic assignments unless the evidence was sufficiently strong.

We also added a rooted mitochondrial phylogenomic tree as Fig. 4 to place the unclassified fraction in broader evolutionary context. This analysis shows where these lineages fall relative to reference mitochondrial genomes, while preserving conservative taxonomy where current databases remain too sparse for confident naming.

**Comment 3:** *"A COI tree and a SSU/LSU tree is convenient, but given*

*that mitochondrial genomes are being recovered, a larger tree with more subunits could be generated. Inclusion of COB at least would increase resolution.*
*Similarly, trees with this many branches are subject to artefact, is it necessary to include so many branches? some degree of identity could be used to simplify the tree.*
*ALSO: why not include branch lengths? A cladogram is only somewhat useful.*
*Why are the trees rooted on Stramenopiles?*
*A stripped down concatenated tree using ALL electron transport chain proteins regularly found in the mtDNA could help resolve your tree a bit better and identify any new lineages. Our Butenko 2024 paper in BMC biology should have all the necessary sequences. Of course, you would need to drastically decrease the number of taxa included in your tree."*

**Response 3:** We thank the reviewer for this suggestion. We have added a new mitochondrial phylogenomic tree as Fig. 4.

For this analysis, we first dereplicated the reference set by retaining one representative mitochondrial genome per genus from RefSeq r226 and all the public SAR mitogenome collection (see Methods). We also dereplicated the FMA by clustering species-level representatives with MMseqs2 at 70% sequence identity and query-side coverage ≥80%, resulting in 2,160 non-redundant FMA representatives. These were combined with 821 dereplicated reference mitogenomes, giving a master dataset of 2,981 mitochondrial genomes.

We then screened all genomes against a custom HMM database built from 24 conserved MitoCOGs. The marker set included NADH dehydrogenase subunits 1, 2, 4, 5, 6, 7, 8, 9 and 11; cytochrome c oxidase subunits 1, 2 and 3; cytochrome b; ATP synthase subunits 1, 3 and 6; cytochrome c maturation protein ccmFN; and mitochondrial ribosomal proteins L2, L6, L11, L16, S4, S12 and S14. Genomes with fewer than seven recovered markers were excluded. Marker alignments were refined, trimmed, filtered to remove the fastest-evolving 10% of sites, and concatenated into a final amino-acid supermatrix containing 1,936 mitochondrial genomes. The tree was inferred in IQ-TREE using a guide tree under LG+F+R10 followed by a PMSF approximation with the LG+C60+F+G model.

The new tree is shown with branch lengths and is rooted with a bacterial outgroup. Thus, the revised manuscript now includes a reduced, branch-length-scaled, rooted mitochondrial phylogenomic tree based on multiple conserved mitochondrial proteins, rather than relying only on the COI and rRNA marker trees.

**Comment 4:** *"Do you have a sense of your false negative rate? Many "NEW" mtDNAs that we discover in our lab hit mtDNAs and bacterial sequences with similar BLAST evalues. It can be somewhat difficult to be certain. AND MANY of our most interesting discoveries would NEVER be picked up by any automated system. So, I'm curious. How certain are you of your rejected contigs?"*

**Response 4:** We agree that some genuinely mitochondrial contigs may have been excluded, especially if they were short, highly divergent, gene-poor, or lacked recognizable mitochondrial markers. We therefore do not interpret the rejected contigs as demonstrably non-mitochondrial in every case. Rather, our filtering defines the boundary

of a conservative atlas: contigs were retained only when they carried enough independent evidence to support downstream curation, classification, clustering and phylogenetic analysis.

This was an intentional choice. The FMA was designed as a high-confidence resource, not as an exhaustive collection of every possible mitochondrial fragment in the assemblies. We first required contigs to be at least 5 kbp, because shorter fragments provide limited sequence context for machine-learning classification and downstream validation. Candidate representatives were then assigned genetic codes, translated with Prodigal, and screened against TIGRFAMs with HMMER. Representatives with two or fewer annotated genes were removed because they did not contain enough interpretable signal for reliable mitochondrial validation. Remaining candidates were further evaluated using rRNA-based searches, protein-based LCA classification against a mitochondria-plus-bacteria reference set, and an independent panel of classifiers to screen for viral, bacterial, plastid and nuclear contamination.

The properties of the discarded representatives support this conservative decision. They were generally small and poorly informative, with a median size of 6.711 kbp, a median cluster size of 1, a median of 0 recovered 12S rRNA markers, 0 recovered 16S rRNA markers, and 0 TIGRFAM annotations.

We therefore cannot provide a precise false-negative rate, because there is no independent ground truth for rejected environmental contigs. The false-negative rate is almost certainly non-zero. Some discarded contigs may represent genuine but too-fragmentary or gene-poor mitochondrial sequences/genomic regions. However, retaining such weakly supported candidates would have introduced a high risk of false positives and would have made species counts, phylogenetic placement and ecological comparisons less robust. For the purposes of this manuscript, we therefore preferred to exclude contigs that could not be validated with sufficient confidence, even at the cost of missing some real mitochondrial fragments.

**Comment 5:** *"The 748 unclassifiable mtDNAs should be manually checked, especially any that are complete or near complete. This will help understand how many new lineages you may have identified."*

**Response 5:** We re-examined the unclassified fraction using several complementary lines of evidence. First, all records had already passed the proteome-based LCA workflow, but these 748 representatives remained taxonomically unassigned because the available protein evidence did not support a confident classification. Second, for unclassified representatives containing COI, we searched the COI sequences against NCBI nr and report the closest matches, sequence identities and taxonomies in Supplementary Data S6.3. Third, we added a new rooted mitochondrial phylogenomic tree based on multiple conserved mitochondrial markers to place the unclassified representatives in broader evolutionary context.

This analysis shows that the unclassified fraction is not a single unresolved pool. Several groups of unclassified mitoMAGs fall near recognizable but reference-poor regions of the eukaryotic tree, including Rhizaria/Cercozoa-like branches, choanoflagellate-like lineages, Cryptophyceae-like clades, Bigyra-like stramenopiles, fungal-like lineages and smaller Amoebozoa-, Apusozoa- or Discoba-related regions. We now use this tree to show where these lineages are positioned, while retaining conservative "unclassified" labels when the evidence does not justify a formal lower-rank assignment.

**REVIEWER 3**

**Comment "Remarks to the Author":** *"I co-reviewed this manuscript with one of the reviewers who provided the listed reports. This is part of the Nature Communications initiative to facilitate training in peer review and to provide appropriate recognition for Early Career Researchers who co-review manuscripts."*

**Response:** We thank Reviewer #3 for contributing to the assessment of our manuscript. We appreciate the time and care devoted to the review, and we welcome the Nature Communications initiative that supports training and recognition for Early Career Researchers involved in peer review.

**REVIEWER 4**

**Comment "Remarks to the Author":** *"This paper explores a large dataset of mitogenomes from protists occurring in lakes. Generating this dataset and analysing it has substantial merit. One of the main and most interesting findings is the new diversity of mitogenomes across several protist groups that were previously underrepresented."*

**Response:** We thank the reviewer for their careful reading of the manuscript and for their positive assessment of the work. The reviewer's comments helped us clarify the scope of the atlas, refine the terminology used to describe the recovered genomic information, and strengthen the presentation of the newly recovered diversity. We have revised the manuscript accordingly and respond to each point in detail below.

**Major Comments**

**Comment 1:** *"The work claims to be "global", while the vast majority of samples come from North America and Europe (Fig1). This should be rephrased in the title and the rest of the manuscript. E.g., there seem to be a couple of samples from the entire South American continent, a few from Africa, and one or a handful from Australia. The dataset is quite impressive as it is, but it does not cover several parts of the world."*

**Response 1:** We agree that the geographic distribution of the dataset is uneven, and we have revised the manuscript to state this more explicitly. We have retained "global" in the title because the study integrates freshwater metagenomes from 362 freshwater bodies across 28 countries, seven continents, and major climatic zones, rather than representing a single regional lake survey. However, we now make clear that the FMA is a global-scale resource, not an exhaustive or proportional census of all lake regions.

The stronger representation of North America and Europe partly reflects the current availability of deeply sequenced freshwater ecosystems. It also occurs against the background of the natural distribution of lakes: published global lake inventories estimate that approximately 85% of lakes occur in the Northern Hemisphere, with particularly high lake densities across formerly glaciated regions of northern North America and Eurasia. Thus, a Northern Hemisphere bias is expected for any large freshwater lake synthesis, although we agree that the FMA remains more strongly represented in North America and Europe than in several tropical and Southern Hemisphere regions.

<u>We have revised the manuscript accordingly</u>. In the Results, we replaced "*geoclimatically balanced compendium*" with "*geographically and climatically broad compendium*". In the newly created Limitations section, we now state that geographic coverage remains uneven, that tropical and Southern Hemisphere systems are comparatively underrepresented, and that future expansion should prioritize these regions. At the same time, we note that the FMA does include tropical and Southern Hemisphere systems, including major African lakes such as Tanganyika and Kivu. We therefore retain "global" to denote the multi-continental scope of the atlas, while clarifying that the resource is not geographically exhaustive.

We have added the following paragraph in the Limitations section (Lines: 839-846): *"Geographic coverage remains uneven, reflecting both the availability of deeply sequenced freshwater metagenomes and the underlying distribution of lake systems. Global lake inventories show a strong Northern Hemisphere concentration, especially across formerly glaciated regions of North America and Eurasia[92]. The FMA should therefore be viewed as a global-scale but non-exhaustive resource: it includes tropical and Southern Hemisphere systems, while making clear that these regions remain priorities for future expansion."*

**Comment 2:** "*Heteroplasmy and microchimerism: I think microchimerism arising from metagenomic assembly should be discussed in depth, given how it could affect diversity metrics (richness, PD, etc.). Assuming heteroplasmy is occurring (i.e., slightly different mitochondrial genomes in the same cell), during assembly, a consensus or "chimeric" mitogenome could be generated. Something similar could happen with mitogenomes from closely related taxa. This is probably mitigated to some extent by the applied clustering, but I think it should be tested against mitogenomes produced from single cells, single mitochondria, etc. Connected to this, multiple assemblers have been used, even different versions of the same assembler. Given that the performance of some assemblers can differ (e.g., Spades vs. megahit), it should be discussed how this could affect the results.*"

**Response 2:**

**A.** Heteroplasmy and microchimerism

We agree that heteroplasmy should be considered explicitly, but its relevance to the FMA depends on scale. Heteroplasmy is widespread and can occur at several biological levels, from individual organelles to cells, tissues and populations[8]. However, much of the heteroplasmy detected by deep sequencing is low-level and SNP-scale, with many variants occurring at low allele fractions[9] rather than representing genome-wide divergence approaching species-level mitochondrial distances. For example, human mitochondrial DNA heteroplasmy has been described as involving numerous independent variants, each usually present in only ~1–2% of mitochondrial genomes, while mutation-accumulation work in *Caenorhabditis elegans* estimates mitochondrial mutation rates on the order of $10^{-7}$ substitutions per site per generation[10]. These values support the expectation that ordinary low-level heteroplasmy is unlikely, by itself, to move a mitochondrial sequence across the 98.1% whole-sequence identity threshold used for FMA species-level clustering.

Because bulk metagenomic data cannot distinguish true intracellular heteroplasmy from within-population mitochondrial polymorphism or closely related mitochondrial haplotypes mapping to the same representative, we quantified the combined signal as read-level mitochondrial microdiversity. We performed this analysis in Lake Stechlin, one of the most deeply sampled systems in the dataset. The 1,172 mitoMAGs detected in Lake Stechlin were clustered into 398 species-level representatives, and quality-filtered Illumina reads from 299 metagenomes were mapped back to these representatives. We then used inStrain v1.8.0 to quantify divergent sites directly from the metagenomic read data.

Across 2,690 retained mitoMAG profiles, the mean number of divergent sites was 2.82 ± 4.43 per Kbp, with a median of 1.07 per Kbp. This corresponds to approximately 0.282 ± 0.443% mean divergence and 0.107% median divergence. Importantly, 98.74% of profiles remained below 19 divergent sites/Kbp, the divergence equivalent of the 98.1% species-level threshold; only 1.26% exceeded this boundary. We therefore treat heteroplasmy-like mitochondrial variation as a real but bounded source of uncertainty. The read-level data indicate that, in a deeply sampled lake system, mitochondrial microdiversity is usually far below the level required to alter species-level clustering, although rare high-divergence cases may likely reflect the presence of closely related lineages.

We added the following section to the Supplementary Information:

"Read-level mitochondrial microdiversity analysis

To evaluate whether within-sample mitochondrial variation could influence mitoMAG interpretation, we performed a read-level microdiversity analysis on Lake Stechlin, Germany, one of the most deeply sampled systems in the dataset. This analysis was designed to quantify mitochondrial sequence variation directly from raw metagenomic reads, before consensus assembly. Because bulk metagenomic data cannot distinguish true intracellular heteroplasmy from within-population mitochondrial polymorphism or the co-occurrence of closely related mitochondrial haplotypes, we interpret this analysis as an upper-bound estimate of heteroplasmy-like mitochondrial microdiversity around recovered mitoMAGs.

Lake Stechlin contained 1,172 detected mitoMAGs across 299 Illumina shotgun metagenomes. To reduce redundancy and computational complexity, these mitoMAGs were clustered using the same species-level clustering procedure applied in the main analysis, yielding 398 representative mitoMAGs. Quality-filtered Illumina reads from the 299 metagenomes were mapped against these representative mitoMAGs using BBMap. The resulting sorted BAM files were analysed with inStrain v1.8.0 using default settings to quantify read-level nucleotide variation across the recovered mitochondrial representatives.

Profiles were retained only when the mitoMAG had at least 2× mean coverage and at least 50% breadth of coverage. The 50% breadth threshold follows inStrain recommendations for robust genome detection and reduces false-positive profiles caused by sparse or local read recruitment. For each retained mitoMAG profile, we extracted the number of divergent sites normalized per kilobase. These values were then compared with the divergence implied by the FMA species-level clustering boundary. Because the species-level cutoff is 98.1% sequence identity, the corresponding divergence boundary is 1.9%, equivalent to 19 divergent sites per kilobase.

The density of divergent sites was generally low. Across retained profiles, the mean was 2.82 ± 4.43 divergent sites per kilobase, with a median of 1.07 divergent sites per kilobase and an interquartile range of 0.22–3.56 divergent sites per kilobase. Expressed as approximate sequence divergence, this corresponds to a mean of 0.282 ± 0.443% and a median of 0.107%. Thus, most read-level mitochondrial variation was well below the 1.9%

divergence corresponding to the 98.1% species-level clustering threshold used in the FMA.

Consistent with this, 2,656 of 2,690 retained mitoMAG profiles, corresponding to 98.74%, remained below 19 divergent sites per kilobase. Only 34 profiles, or 1.26%, exceeded this boundary. These high-divergence cases are unlikely to represent ordinary low-level heteroplasmy alone. More plausibly, they reflect the presence of closely related mitochondrial lineages within the same sample. We therefore interpret them as rare cases of elevated mitochondrial microdiversity rather than as evidence that heteroplasmy broadly affects species-level clustering.

Overall, this read-level analysis indicates that mitochondrial microdiversity in Lake Stechlin is usually far below the sequence divergence required to cross the FMA species-level boundary. Heteroplasmy-like variation is therefore unlikely to be a major driver of the species-level richness or phylogenetic diversity patterns reported in the main manuscript."

**B.** Sequencing technology and assembler effects

To address the reviewer's concern regarding assembler-dependent effects, we added a proof-of-concept analysis using a ZymoBIOMICS mock community in which *Saccharomyces cerevisiae* represented the eukaryotic component within a bacteria-dominated background. The same mock community was sequenced using Illumina short reads and Oxford Nanopore long reads, and assemblies were generated independently with MEGAHIT and metaSPAdes for Illumina data, and metaFlye and Canu for Nanopore data. We then recovered the *S. cerevisiae* mitochondrial genome/genome fragments from each assembly and compared the recovered sequences pairwise using ANI.

The recovered mitochondrial sequences were highly concordant across sequencing platforms and assemblers. Pairwise identities ranged from 99.94% to 100%, with a median identity of 99.97%. These values are well above the 98.1% identity threshold used for species-level clustering in the FMA. Thus, in this controlled community, when the same mitochondrial unit was recovered, assembler and sequencing-platform differences did not move the sequence across the species-level boundary used in our analyses.

We now present this result as a focused control for sequence-identity stability, rather than as a general claim that assembler choice has no effect. Assembly strategy can still influence recovery sensitivity, contiguity and fragmentation, particularly in low-coverage or highly complex environmental samples, <u>and we now state this explicitly as a methodological caveat in the Limitations section of the manuscript</u>. However, the mock-community comparison supports the central point relevant to species delineation: assembler/platform variation among shared recoveries was much smaller than the 1.9% divergence allowed by the FMA clustering threshold. This is also consistent with previous mock-community metagenomic benchmarks showing that shotgun sequencing and assembly can recover expected genomes or contigs at very high sequence identity when coverage is sufficient[1]. We have added this analysis to the revised Supplementary Information under "Mock-community benchmarking of sequencing and assembly effects."

Reference:

1. Meyer, F. et al. Critical Assessment of Metagenome Interpretation: the second round of challenges. Nature methods 19, 429–440; 10.1038/s41592-022-01431-4 (2022).

**Comment 3**: "*Size fractions - It is mentioned that most samples originated from the 0.2-5um size fraction. This covers mostly the pico-plankton (0.2-3um), and a small portion of the nano-plankton (3-20um). It does not cover the micro-plankton (20-200um). Thus, the sentence in Line 554, indicating "...facilitating the recovery of mitochondrial genomes from a broad size spectrum of eukaryotic organisms …" does not seem correct, and should be rephrased, indicating that the dataset predominantly represents the signal recovered in the pico-plankton.*"

**Response 3:** We agree that the original wording was imprecise. The sentence was intended to describe the filtration fractions represented in the underlying datasets, but it could be read as implying uniform recovery across all planktonic size classes. We have revised the text to make the filtration scheme explicit.

The pore sizes and sampled fractions are reported in Supplementary Data S1/S2. In datasets where both fractions were collected, the 0.22-µm filters correspond to the 0.22–5 µm fraction, whereas the 5-µm filters correspond to the >5 µm fraction. Thus, the dataset includes both small-cell fractions and larger retained biomass where the >5 µm fraction was sequenced. We now describe the FMA as representing the mitochondrial signal retained by the sampled filtration fractions, rather than as an even survey of all planktonic size classes.

The recovered taxonomic record also shows that the FMA is not restricted to pico- and nanoplankton-derived signal, as it includes metazoan-affiliated mitoMAGs from rotifers, crustacean zooplankton, freshwater bivalves, chironomids and water mites. The full taxonomic record is provided in Supplementary Data S6.

We added the following clarification in the Methods section (Lines 1035-1048): "*Samples included different filtration schemes depending on the original study design, with pore sizes and sampled fractions reported in Supplementary Data S1/S2. Where both fractions were collected, 0.22-µm filters corresponded to the 0.22–5 µm fraction, whereas 5-µm filters corresponded to the >5 µm fraction. We therefore interpret the recovered mitoMAGs as the mitochondrial signal retained by the sampled filtration fractions, rather than as uniform coverage of all planktonic size classes*."

**Comment 4:** "*Some ecological questions addressed with the produced dataset (e.g., those relating mitogenomes with the trophic state of lakes) seem a bit shallow and too broad, leading to confirmatory results. I wonder whether the manuscript could focus more on the new insights derived from exploiting this large, new, dataset.*"

**Response 4:** We thank the reviewer for this comment. We understand the concern that some of the ecological questions addressed in the manuscript, particularly those relating mitoMAG recovery to lake trophic state, may appear broad or confirmatory when viewed from the perspective of established lake ecological theory. However, our aim was not only

to catalogue mitochondrial diversity, but also to establish and evaluate a framework through which metagenome-derived mitochondrial reconstructions can be used for species-resolved, phylogeny-aware assessments of freshwater microeukaryote diversity.

For this reason, we consider the ecological analyses an integral part of the manuscript. Their purpose is not to re-establish well-known ecological principles per se, but to test whether a newly generated, mitochondrial-genome-based resource can recover biologically coherent patterns across heterogeneous shotgun metagenomic datasets. The recovery of expected productivity-, season-, and depth-associated trends is therefore important because it provides an ecological validation of the framework. In this context, confirmatory results are informative: they show that the FMA does not only expand reference space, but can also be used to derive standardised and interpretable community-level metrics across lakes, trophic states, seasons, and water-column strata.

At the same time, we agree with the reviewer that the manuscript should place stronger emphasis on the new insights made possible by exploiting this large dataset. We have therefore expanded the Results and Discussion sections to better highlight the diversity uncovered by the FMA beyond the ecological validation analyses. In particular, we added a new phylogenomic analysis focused on the placement of FMA representatives (Fig. 4), including taxonomically unclassified or poorly resolved mitoMAGs, in relation to currently described mitochondrial diversity. This analysis allows us to move beyond reporting that many recovered sequences are distant from references and instead shows where these divergent lineages fall across the eukaryotic tree.

The new analysis reveals that the previously unclassified component of the FMA is not a homogeneous residual category, but is distributed across several phylogenetic contexts, including branches associated with Stramenopiles, Rhizaria/Cercozoa, Cryptophyceae, Choanoflagellata, Amoebozoa, Haptista, Fungi, Viridiplantae, and other poorly sampled eukaryotic lineages. This provides a more explicit view of the novel mitochondrial diversity recovered by the atlas and helps distinguish broad database incompleteness from lineage-specific expansions in poorly represented freshwater groups. We now discuss these placements as one of the main insights emerging from exploiting the scale of the FMA dataset.

We believe this revision better balances the two contributions of the manuscript. The ecological analyses remain important because they demonstrate that mitoMAG-based inventories recover interpretable and theory-consistent community patterns across heterogeneous freshwater metagenomes. The new phylogenomic analysis, however, more directly addresses the reviewer's request to emphasize novel insights from the dataset by showing how the FMA expands the phylogenetic landscape of freshwater mitochondrial diversity, especially for lineages that are currently poorly represented or unclassified in reference databases.

**Specific comments:**

**Comment 5:** *"L76 - Cyanobacteria also contribute to primary production in the ocean…"*

**Response 5:** We thank the reviewer for this comment. We agree that cyanobacteria are major contributors to primary production in the open ocean. The sentence in question was not intended as a complete inventory of marine primary producers, but as part of a paragraph introducing the ecological importance of microbial eukaryotes. In that context, the statement that microeukaryotes generate "*more than half of marine primary production*" is supported by the cited literature and highlights their major contribution to ocean productivity and biogeochemical cycling. This wording does not imply that microeukaryotes account for all primary production, nor does it diminish the well-established role of cyanobacteria. We have therefore preferred to maintain the sentence unchanged.

**Comment 6:** "*L78 - In freshwater AND marine ecosystems…*"

**Response 6:** We agree that these ecological roles of microbial eukaryotes are relevant in both freshwater and marine ecosystems. We have therefore made the suggested modification and revised the sentence accordingly.

**Comment 7:** "*Fig1 - I'd remove the word "Global" from the legend.*"

**Response 7:** We have removed the term "Global" from the legend of Fig. 1. The legend now reads: "*Fig. 1 | Coverage of the Freshwater Mitochondrial Atlas across Köppen–Geiger climate zones*."

**Comment 8:** "*Fig4 - What are the differences between the two figures in panel A)?*"

**Response 8:** We thank the reviewer for this comment and for the opportunity to clarify Fig. 4a. The two plots in panel A are not separate analyses, but two complementary Hill-number diversity orders calculated from the same trophic-state dataset at standardized sample coverage. The left plot shows Shannon diversity expressed as effective species numbers, corresponding to Hill diversity of order q = 1. The right plot shows inverse Simpson diversity, corresponding to Hill diversity of order q = 2.

We included both because they capture complementary aspects of the trophic-state diversity pattern. Shannon diversity expressed as Hill numbers (q = 1) estimates the effective number of common species-level mitoMAG units and is influenced by both richness and evenness across the broader assemblage, including moderately frequent lineages. Inverse Simpson diversity (q = 2) gives greater weight to the most frequently recovered species-level units and is therefore more sensitive to dominance structure and less influenced by rare lineages. Thus, comparing q = 1 and q = 2 allows us to distinguish whether the observed mesotrophic diversity peak reflects a broader increase in assemblage richness/evenness or is also apparent among the dominant, repeatedly recovered lineages.

In the revised figure, we have clarified this distinction by explicitly labelling the two plots as “Shannon diversity, q = 1” and “Inverse Simpson diversity, q = 2”, and by updating the Fig. 4 legend accordingly. This makes clear that panel A shows two complementary effective-diversity estimates, rather than duplicated or unrelated plots.

We added the following paragraph to the Fig. 4 legend (Lines 558-563): “*Left: Shannon diversity expressed as effective species numbers, corresponding to Hill diversity of order q = 1. Right: inverse Simpson diversity expressed as effective species numbers, corresponding to Hill diversity of order q = 2.*”

**Comment 9:** “*L406 - The information that can be conveyed by phylogenetic overclustering or overdispersion should be explained more here.*”

**Response 9:** We agree that the information conveyed by phylogenetic clustering and overdispersion should be made clearer in this section of the Discussion. Our intention was to emphasize that placing species-level mitoMAG units on a common phylogeny adds an additional layer of information beyond species counts alone.

We have added the following paragraph to the discussion section (Lines 615-627): “*In this framework, phylogenetic clustering and overdispersion describe the architecture of diversity on the tree. Clustering indicates that species-level units are concentrated within closely related lineages, whereas overdispersion indicates that they span more distant branches. This matters because richness can remain unchanged while the evolutionary composition of a community is reorganized: turnover may occur among near relatives within the same lineage pool, or through replacement and recruitment across deeper regions of the eukaryotic tree. The latter represents a deeper form of community change, because it alters not only how many species-level units are present, but which parts of eukaryotic evolutionary history are represented. The phylogenetic scaffold therefore allows the FMA to detect phylogenetic homogenization, expansion, or turnover that would be invisible from species counts alone.*”

**Comment 10:** “*L471 - Could this diversity be inflated due to microchimerism and heteroplasmy?*”

**Response 10:**

We agree that individual-level mitochondrial variation can complicate the interpretation of mitochondrial sequence diversity, and we have considered this possibility carefully. We distinguish here between microchimerism and heteroplasmy, because they have different implications for our dataset.

Microchimerism, strictly defined, refers to the presence of a small population of genetically distinct cells within an individual. Such cells carry mitochondrial genomes from their own cell lineage, but microchimerism is not itself a process that generates novel mitochondrial sequence divergence. In the context of bulk environmental metagenomes, mitochondrial DNA from such cells would enter the same mixed community DNA pool as

other biological material. Because the FMA does not assign mitoMAGs to individual host organisms, but instead reconstructs mitochondrial genomic units from environmental DNA, microchimerism is unlikely to inflate diversity in the way it might complicate individual-level mitochondrial genotyping.

Heteroplasmy is more directly relevant to mitochondrial sequence variation, because it reflects the coexistence of different mtDNA variants within an individual or cell lineage. However, heteroplasmic variation is expected primarily to affect fine-scale haplotypic diversity. The statement in the manuscript refers to a much broader signal: "only ~11.2% of recovered FMA COI sequences exceeded 85% identity to eKOI references". Divergence at this scale is unlikely to be explained by within-individual mitochondrial polymorphism, and is more consistent with undersampling of freshwater mitochondrial diversity in current reference databases. In addition, the FMA is based on genome-resolved mitochondrial reconstructions rather than amplicon-level variants. Low-frequency mitochondrial variants would generally be collapsed into consensus assemblies, remain unsupported, or be absorbed within the same species-level mitoMAG unit under the 98.1% genome-identity clustering framework. This makes heteroplasmy unlikely to be a dominant driver of the broad reference divergence reported here.

Moreover, several empirical features of the FMA support a biological rather than artefactual origin of the recovered diversity. We recovered identical or near-identical mitochondrial sequences from geographically separated ecosystems and from temporally distinct samples. Such reproducibility across independent metagenomes is not expected if stochastic individual-level heteroplasmy or low-frequency microchimeric signals were the dominant driver of the observed diversity. Instead, it indicates repeated recovery of the same mitochondrial genotypes from independent environmental samples. In addition, although only a minority of FMA records have close database matches, some sequences are 100% identical to mitochondrial sequences independently recovered from cultured organisms or environmental sources. This shows that the pipeline can recover known mitochondrial sequences when close references exist.

We therefore cannot exclude that heteroplasmy or rare individual-level cell-lineage mixtures contribute to fine-scale mitochondrial variation in some cases. However, these processes are unlikely to explain the large-scale divergence from reference databases reported here.

<u>We introduced the following paragraph into our Limitation chapter (Lines 960 -969)</u>:
"*Individual-level mitochondrial variation represents an additional caveat. Heteroplasmy, microchimerism, within-population polymorphism and the co-occurrence of closely related lineages can all introduce mitochondrial sequence heterogeneity into environmental assemblies. In the FMA, these signals are partly absorbed by consensus assembly and species-level clustering, and are therefore expected to affect mainly fine-scale haplotype structure rather than broad species-level or phylogenetic patterns. They remain relevant for interpreting rare high-divergence profiles and local sequence variation, but are unlikely to explain the large reference divergence or the major diversity gradients reported here.*"

**Comment 11:** "*L500 - I think this paragraph should discuss some points more specifically:*

*microchimerisms, the use of different assemblers and how this can affect results, the distribution of the samples (mostly N.America and Europe), as well as the size fractions from which metagenomes were generated (mostly picoplankton)."*

**Response 11:** We agree that these limitations should be discussed more explicitly. In response to this comment, and to related comments raised by the other reviewers, we have added a dedicated Limitations section in which we now address the main biological, technical, and sampling biases that may influence the FMA.

**Comment 12:** *"L534 - There are ca. 16 pages of methods. It'd be good to reduce this section."*

**Response 12:** We agree that the Methods section is extensive. We have retained it in the main manuscript because the study combines many datasets and includes multiple filtering, clustering, annotation and validation steps; and we wanted the workflow to remain transparent and reproducible for readers.

We also note that, for a related manuscript published in the same journal, we were asked to move methodological details from the Supplementary Information into the main Methods section. For this reason, we have kept the full core methodology in the main text. If the editor prefers a shorter Methods section, we would of course be happy to move selected technical details to the Supplementary Information.

**Comment 13:** *"L555 - Most metageomes represent the picoplankton and are probably enriched in picoeukaryotes. Discuss the signal of nanoeukaryotes and microeukaryotes and how this can be related to the sampling process, etc."*

**Response 13:** We agree that the relationship between filtration and the recovered eukaryotic signal needed clearer explanation. The manuscript has now been revised to specify the sampled fractions explicitly. The pore sizes and corresponding fractions are reported in Supplementary Data S6.1/S6.2. In datasets where both fractions were collected, the 0.22-μm filters correspond to the 0.22–5 μm fraction, whereas the 5-μm filters correspond to the >5 μm fraction. Thus, although many metagenomes include the 0.22–5 μm fraction and are expected to contain strong picoeukaryotic and small-nanoeukaryotic signal, the dataset also includes >5 μm fractions, which retain larger nanoeukaryotes, microeukaryotes, colonies, propagules, tissue fragments and particle-associated mitochondrial DNA.

The recovered taxonomy supports this broader interpretation. We do recover lineages compatible with picoeukaryotic diversity, including Mamiellophyceae and Chloropicophyceae. However, the FMA is not restricted to this component. It also includes lineages commonly represented among nanoeukaryotes or spanning nano- to microeukaryotic size ranges, including haptophytes, cryptophytes, katablepharids, choanoflagellates, ochrophytes and chlorophytes. In addition, the dataset contains clear microeukaryotic signal, most prominently ciliates, which account for 571 species-level representatives and 3,778 mitoMAGs. We also recovered mitochondrial signal from larger

eukaryotic lineages, including arthropods, rotifers/Gnathifera, freshwater bivalves, chironomids and water mites.

We therefore interpret the FMA as the mitochondrial signal recovered from the sampled 0.22–5 µm and >5 µm fractions, rather than as a survey restricted to picoplankton. At the same time, we avoid claiming size-unbiased coverage of all planktonic classes, because recovery depends on the filtration scheme, biomass retained, DNA release, sequencing depth and assembly success. The full taxonomic record is provided in Supplementary Data S6.

**Comment 14:** "*L558 - More information should be provided about the parameters used with the different assemblers, naming all those that have been used.*"

**Response 14:** We thank the reviewer for this comment. The assembler names, versions and dataset-specific parameters are provided in Supplementary Data S1 and, where applicable, also in the associated ENA project records. To make this information more accessible, we have now added a concise assembly-workflow summary to the Methods section, immediately after the dataset description. This paragraph lists the assemblers used, their version ranges, main k-mer ranges or options, polishing tools, read-mapping tools and preprocessing workflows.

We added the following paragraph to the Methods section (Lines 922-947): "*Briefly, reads were quality-filtered and trimmed using dataset-specific workflows, including Cutadapt v2.5, fastp v0.20.0, BBMap/BBNorm, Trimmomatic, PRINSEQ and Sickle. Processed reads were assembled with short- or long-read metagenomic assemblers depending on the sequencing platform and original dataset. Short-read assemblies used metaSPAdes v3.8.0–3.15 with k-mer sizes of 21–127, or MEGAHIT v1.1.3–1.2.9 with k-mer sizes of 29–149. Long-read assemblies used Flye v2.8 with --plasmids and --meta, or Raven v1.5.0. Where applicable, assemblies were polished with Racon v1.4.13, Medaka v1.0.3 and/or Pilon v1.23 after read mapping with Minimap2 v2.17, Bowtie2 v2.3.5.1 or BWA-MEM v0.7.12. Unless otherwise stated in Supplementary Data S1, default parameters were used.*"

## References


1. Luo, C., Tsementzi, D., Kyrpides, N. C. & Konstantinidis, K. T. Individual genome assembly from complex community short-read metagenomic datasets. *The ISME journal* **6,** 898–901; 10.1038/ismej.2011.147 (2012).

2. Serra Moncadas, L., Hofer, C., Bulzu, P.-A., Pernthaler, J. & Andrei, A.-S. Freshwater genome-reduced bacteria exhibit pervasive episodes of adaptive stasis. *Nature communications* **15,** 3421; 10.1038/s41467-024-47767-7 (2024).

3. Liu, L., Yang, Y., Deng, Y. & Zhang, T. Nanopore long-read-only metagenomics enables complete and high-quality genome reconstruction from mock and complex metagenomes. *Microbiome* **10,** 209; 10.1186/s40168-022-01415-8 (2022).

4. Nicholls, S. M., Quick, J. C., Tang, S. & Loman, N. J. Ultra-deep, long-read nanopore sequencing of mock microbial community standards. *GigaScience* **8**; 10.1093/gigascience/giz043 (2019).

5. Gray, M. W., Lang, B. F. & Burger, G. Mitochondria of protists. *Annual review of genetics* **38,** 477–524; 10.1146/annurev.genet.37.110801.142526 (2004).

6. van der Giezen, M. Hydrogenosomes and mitosomes: conservation and evolution of functions. *The Journal of eukaryotic microbiology* **56,** 221–231; 10.1111/j.1550-7408.2009.00407.x (2009).

7. Najer, T. *et al.* Mitochondrial genome fragmentation is correlated with increased rates of molecular evolution. *PLoS genetics* **20,** e1011266; 10.1371/journal.pgen.1011266 (2024).

8. Stewart, J. B. & Chinnery, P. F. The dynamics of mitochondrial DNA heteroplasmy: implications for human health and disease. *Nature reviews. Genetics* **16,** 530–542; 10.1038/nrg3966 (2015).

9. Payne, B. A. I. *et al.* Universal heteroplasmy of human mitochondrial DNA. *Human molecular genetics* **22,** 384–390; 10.1093/hmg/dds435 (2013).

10. Konrad, A. *et al.* Mitochondrial Mutation Rate, Spectrum and Heteroplasmy in Caenorhabditis elegans Spontaneous Mutation Accumulation Lines of Differing Population Size. *Molecular biology and evolution* **34,** 1319–1334; 10.1093/molbev/msx051 (2017).
